# A composite annual-resolution stalagmite record of North Atlantic climate over the last three millennia

**DOI:** 10.1038/srep10307

**Published:** 2015-06-11

**Authors:** Andy Baker, John C. Hellstrom, Bryce F. J. Kelly, Gregoire Mariethoz, Valerie Trouet

**Affiliations:** 1 Connected Waters Initiative Research Centre, UNSW Australia, Sydney, NSW 2052, Australia; 2 School of Earth Sciences, The University of Melbourne, Melbourne, VIC 3010, Australia; 3 University of Lausanne, Institute of Earth Surface Dynamics (IDYST), UNIL-Mouline, Geopolis, 1015 Lausanne, Switzerland; 4 Laboratory of Tree-Ring Research, University of Arizona, Tucson AZ 85721, USA

## Abstract

Annually laminated stalagmites can be used to construct a precise chronology, and variations in laminae thickness provide an annual growth-rate record that can be used as a proxy for past climate and environmental change. Here, we present and analyse the first composite speleothem annual growth-rate record based on five stalagmites from the same cave system in northwest Scotland, where precipitation is sensitive to North Atlantic climate variability and the winter North Atlantic Oscillation (NAO). Our 3000-year record confirms persistently low growth-rates, reflective of positive NAO states, during the Medieval Climate Anomaly (MCA). Another persistently low growth period occurring at 290-550 CE coincides with the European Migration Period, and a subsequent period of sustained fast growth-rate (negative NAO) from 600-900 AD provides the climate context for the Viking Age in northern and western Europe.

Stalagmites contain a variety of potential palæoclimate proxies, and the sensitivity of any individual stalagmite proxy to climate is determined by many factors, including its location within the cave, the cave morphology, the hydrological flow path, and the overlying soil and vegetation^1^. In NW Scotland, Uamh an Tartair (Roaring Cave) is overlain by blanket peat, which accumulated over the last ~4000 years^2^, and has a rainfall regime that is highly correlated with the winter NAO^3^. Because of the cave’s shallow depth, an overlying soil carbon and water store, and a blanket peatland that experiences substantial seasonal water table variations, stalagmites within Uamh an Tartair contain annual fluorescent laminae^4^ that are continuous for 10^3^-10^4^ yrs^5^,^6^. These annual laminae provide both an annually resolved chronology and a climate proxy, with the dominant control on annual growth rate being the water table in the overlying peat, which affects soil CO_2_ production and concentration, limestone dissolution, and subsequent stalagmite calcite precipitation. As a result, high (low) lamina growth rates typically reflect warm and/or dry (cool and moist) conditions^5^.

Using a combination of lamina counting and U-Th dating, stalagmite growth rate records from a region can be combined to produce long continuous paleoclimate records. One stalagmite from the site has been previously combined with tree-ring data in a 947-year annual resolution reconstruction of the NAO (NAOms), which suggested an unusual persistence of the positive phase of the NAO during the MCA (1050-1400 CE)^7^. Here, we produce a composite annual growth-rate record based on five stalagmites collected from the Uamh an Tartair cave. Four of the stalagmites have previously been reported^6^,^7^,^8^ and we add a new addition (SU963) to the series.

## Results

### Building a stalagmite growth-rate composite record

Stalagmite SU963 grew on the same flowstone shelf as SU961 and SU962, and contained a continuous lamina sequence for 1802 years with no hiatuses. The growth-rate was determined from multiple manual analyses of images collected using UV microscopy (an example is provided in Supplemental Figure S1). The annual nature of the laminae was confirmed^9^ using U-Th analyses (see Supplementary Table 1 and Methods). Five U-Th analyses constrained the timing of deposition of SU963, between ~1600 and ~400 years before present. Using a combination of the U-Th ages and cross correlation of annual lamina thickness with coeval stalagmites SU961, SU962, and SU967, the annual lamina sequence of 1802 laminae in SU963 was constrained to the period from 22 BCE to 1781 CE (see Methods). This produced a composite stalagmite record of annual growth-rate that has overlapping stalagmites for the period 1475 BCE-1996 CE. Limited replication of the record occurs in the early part of the record, and therefore we focus on the last 3000-yr composite growth-rate record (SU_comp_).

**Figure 1:**
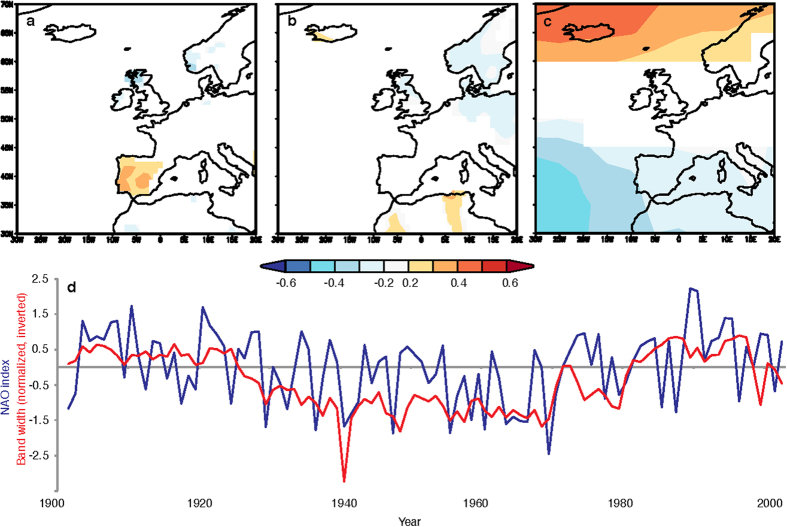
Pearson’s correlation map between SU_comp_ and average fields of Jan-Mar precipitation (**a**), temperature (**b**), and sea level pressure (SLP); **c**). Precipitation and temperature time series were derived from the CRU TS3.22 data set, SLP from HadSLP2r. All correlations were calculated over the period 1901-2004 and climate variable distributions are significant at the 95% confidence level. The SU_comp_ (inversed) and Jan-Mar NAO (Stykkishomur-Azores index) time series (1901-2004; r = −0.46, p < 0.01) are plotted in (**d**). Correlation maps were generated using Climate Explorer^32^.

For the instrumental period, SU_comp_ has the strongest annual correlation with the winter NAO. A positive winter NAO is characterised by increased precipitation in NW Europe, including NW Scotland, and drier conditions in the western Mediterranean. This pattern is observed in the spatial correlation between SU_comp_ and winter (January-March) precipitation, with correlation p < 5% using CRU TS3.22^10^ (Fig. 1a). Spatial correlations are weak with winter (January-March) temperature (Fig. 1b). Strong spatial correlations, with p < 5%, are observed with winter (January-March) sea level pressure (Fig. 1c). This observed pattern is typical of the strong correlations of opposite sign in the two NAO centers of action (the Icelandic Low and the Azores High).

Final evidence of a NAO signal contained within the SU_comp_ series is the temporal correlation with the winter (January-March) NAO index^11^ (Fig. 1d, Stykkishólmur-Azores, 1901-2004; r = −0.46, p < 0.01). The error on this correlation coefficient is determined to be normal with a standard deviation of σ = 0.08, based on a bootstrap analysis^12^, (see Methods). The winter season NAO signal therefore has the predominant correlation with SU_comp_ (climate correlations with other seasons are presented in Supplemental Figure S2). Despite this, an annual correlation of −0.46 indicates substantial noise will be present in this winter NAO signal. We suggest that this is due to several factors that include: (1) an observed weak, but significant, annual correlation between SU_comp_ and spring temperature (See Supplemental Figure S2), which is unrelated to the NAO. (2) Mixing of water in the karst unsaturated zone which smooths the annual climate signal. (3) Variability of the sensitivity of individual stalagmite growth-rate series to the winter NAO, which could be better constrained with a larger stalagmite composite record.

**Figure 2:**
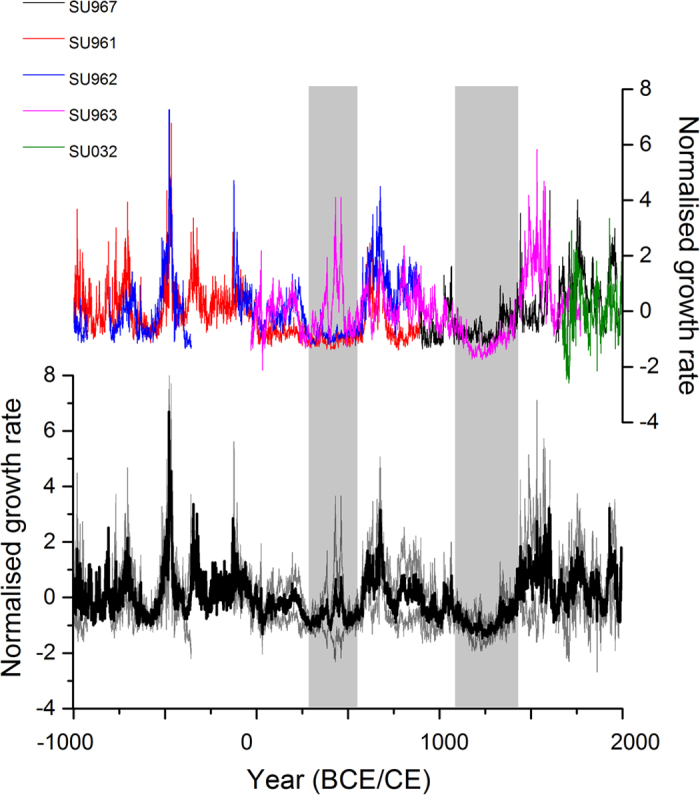
(**a**) Five standardised stalagmite growth-rate series. (**b**) Screened SU_comp_. Data are shown as mean (black) and 1σ variability (grey). Periods where there are low growth runs are shown by the vertical shading.

The 3000-year growth-rate record for the five stalagmites is presented in Fig. 2a. The availability of a composite stalagmite growth-rate record permits the screening of the dataset for climate complacency and non-climatic effects on stalagmite growth-rate. Many techniques have been developed for annual proxy series such as tree rings^13^, but methodologies appropriate to stalagmites – including variogram analysis and growth acceleration^14^ - are required here. Climatically sensitive stalagmites have growth-rates that flicker around a mean state representative of the volume and chemistry of the karst water store supplying the stalagmite. An absence of ‘flickering’ growth in SU967 at 1615-1645 CE demonstrated the dominance of non-linear karst hydrological responses (e.g. fracture storage being full and not climate sensitive) over this time period. Periods of no flickering were also identified in SU963 at 1428-1448 CE. These periods have been removed from the composite series. Similar hydrological effects have been previously noted^7^ when stalagmites ‘die’ due to a lack of stored water supply, with monotonous decreases or increases in growth-rate. With replicate samples available, these effects are clearly visible at the end of each deposition phase in stalagmites SU961 and SU962, and where observed in these specimens (the last 14 years of deposition) the data are removed from the lamina thickness record. Finally, variogram analysis of the information content of the stalagmite growth-rate series suggests that samples all had high information content (SU961, SU962, SU963 and SU967 have information contents of 75, 87, 93 and 70%, respectively, and variogram ranges values of 250, 200, 400 and 60 years, respectively). The new addition to SU_comp_, SU963, has the highest information content and longest range of all samples in the composite, indicating that it is more likely to preserve multi-decadal variability in lamina thickness. The fully screened SU_comp_ is in Fig. 2b (and available as Supplementary Data).

In general, where multiple stalagmites are deposited at the same time, their growth-rates are similar. Despite statistical screening of the data, there are some time periods where one stalagmite has a divergent growth rate. SU963 has faster growth-rate than other samples during periods of below average growth, and SU961 has a slower growth-rate during periods of above average growth. Between sample growth-rate variability should be anticipated due to known non-linear karst processes such as karst hydrology through changes in flow routing^14^ and the non-linear relationship between drip-rate and growth-rate^16^. Replicated, composite series such as SU_comp_ therefore provide the best approach to constraining speleothem growth-rate records with appropriate uncertainty bounds.

### Relating growth-rate persistence and periodicity to climate

**Figure 3:**
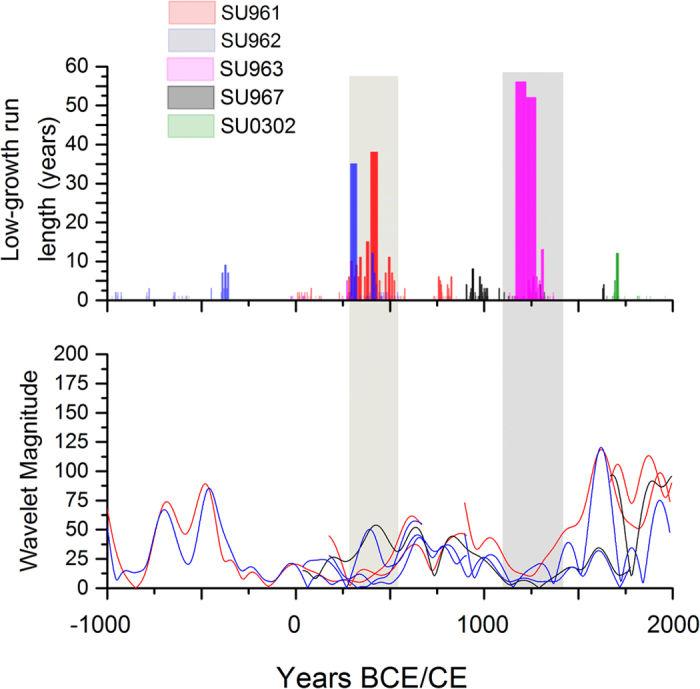
(**a**) Distribution of stalagmite continuous low-growth periods. Low-growth periods are defined as a growth rate below the 10^th^ percentile value of a given stalagmite. (**b**) Wavelet magnitude at periodicities of 64 (blue), 76 years (red) and 90 (black) years (red). Wavelet analysis was performed on all five stalagmites using a Morlet transform. Shading shows the two periods of persistently slow stalagmite growth and positive NAO.

Stalagmite SU967 has been previously utilised in the NAO_ms_ reconstruction, where a persistently positive NAO was identified in the MCA^1^. SU_comp_ (Fig. 2b) replicates the SU967 record over the past 1000 years, and confirms the persistently low growth-rates reflective of cool and moist conditions during the MCA. To investigate whether continuous low stalagmite growth during this period has occurred by chance, we analysed the annual growth-rate distribution of each stalagmite. Figure 3a highlights periods when annual growth-rate was persistently below the 10^th^ percentile. Our analysis confirms that persistently low growth is observed for multiple stalagmite samples during the MCA and it is also observed at 250-550 CE.

We performed wavelet analysis on the growth-rate series for all stalagmites. The global wavelet spectra (Supplementary Figure 3) demonstrate significant spectral power at periods greater than 40 years for all stalagmites. Wavelet analysis demonstrates a non-stationarity of spectral power over time, whose magnitude (Fig. 3b) is consistent between replicate stalagmite records. Specifically, multi-decadal to centennial-scale periodicity is present throughout all stalagmite records, but multi-decadal-scale periodicity decreases in magnitude from ~1100 to ~1450 CE (SU967 and SU963) and from ~0 to ~500 CE (SU961, SU962, and SU963). Other records from the North Atlantic region also show multi-decadal variability^17^,^18^, attributed to the Atlantic Multidecadal Oscillation (AMO). Comparison of the timing of persistent slow stalagmite growth (1080-1460 CE and 290-550 CE) and that of low magnitude of decadal to centennial-scale periodicity (~1100 to ~1450 CE and ~0 to ~500 CE) demonstrates that the two are coincident, with the opposite relationship in fast growth periods. Over the past 450 years, evidence has been presented that the AMO is externally forced and is probably related to the Atlantic Meridional Overturning Circulation (AMOC) strength, and also that this forcing is non-stationary over time^19^. If the decadal-scale periodicity observed in our composite stalagmite record is a response to the AMO, then a weakened AMO may have led to persistently positive NAO conditions not just after the Little Ice Age^19^, but also at 1080-1430 CE (the Medieval Climate Anomaly) and at 290-550 CE (the Dark Ages).

### Comparison with NAO proxy records

**Figure 4:**
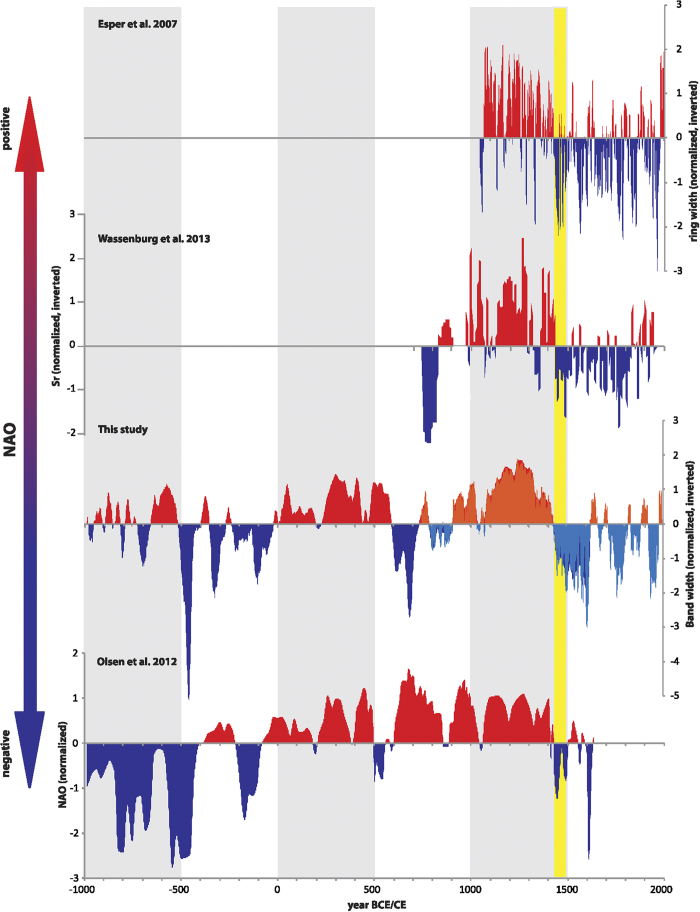
Comparison (991 BCE - 2004 CE) of SU_comp_ (this study) with other proxy records of past NAO variability: a drought-sensitive tree-ring record from Morocco^20^, a drought-sensitive Sr stalagmite record from Morocco^21^, and a West Greenland ice core record^17^. All records were normalised over their full period of record and all records but the ice core record were inverted so that positive values reflect positive NAO conditions. The Moroccan stalagmite and ice-core records are not continuous and these series were smoothed with a cubic spline that closely reflects their average sampling interval (5 years for stalagmite data, 20 years for ice-core data). SU_comp_ was smoothed with a 20-year cubic spline (red and dark blue colors) to match the average resolution of the ice-core record. The most recent portion of SU_comp_ (orange and light blue colors) as well as the tree-ring record were smoothed with a 5-year cubic spline to match the average resolution of the Moroccan stalagmite record. The transition period (1430-1500) between a positive NAO phase during the MCA and a negative NAO phase during the LIA is indicated by a yellow bar.

Given the previously published evidence for the winter NAO sensitivity of annual growth-rates at this site and the strong correlations between SU and winter NAO over the instrumental period (Fig. 1), we interpret SU_comp_ as one of North Atlantic climate and winter NAO. SU_comp_ shows good agreement with other published NAO-sensitive records (Fig. 4), including winter drought-sensitive tree-ring^20^ (1049-1995 CE; r = −0.40; n = 926; p < 0.001 at annual scale and r = −0.56; n = 31; p < 0.01 at tri-decadal scale) and stalagmite records^21^(748-1953 CE; r = 0.47; n = 197; p < 0.001; 5-yr resolution) from Morocco and a West Greenland lake sediment record^17^ (985 BCE-1634 CE; r = 0.34, n = 167, p < 0.001; 20-yr resolution). Comparison of the correlations between all records for a common period (1094-1634 CE) is presented in Supplemental Table 2.

The SU_comp_, Moroccan tree-ring, and Greenland lake sediment archives show a persistently positive winter NAO during the MCA (1080-1430 CE) that is unique within the last 1000 years (although we note that the Greenland lake sediment chronology was tuned to the NAOms series^7^, and would therefore also be expected to correlate with the SU_comp_ reconstruction from 1100 CE onwards). These three records do not observe a more variable NAO state in the MCA as observed in the trace element record of a Moroccan stalagmite^21^. The latter NAO reconstruction was based on Mg/Ca and Sr/Ca ratios in a stalagmite, and we note that these trace element proxies are poorly understood in semi-arid regions where wind-blown dust can complicate signal calibration^22^. A distinct shift from this positive NAO phase into a negative phase during the Little Ice Age occurs simultaneously in all records at ~1430 CE (Fig. 4). The timing of this climate dynamical MCA-LIA transition thus appears synchronous across the North Atlantic realm, although temporal offsets have been recorded in different sub-continental regions^23^.

Prior to the last 1000 years, we observe periods of good agreement in the reconstructed NAO between the two available records, the West Greenland lake sediment record and SU_comp_, for example between 200 BCE – 500 CE. However, there are also periods of difference, especially before 500 BCE. Reasons for the differences could include dating uncertainties in both series (the lake record is constrained by radiocarbon analyses and has a mean age model uncertainty of 54 years) or, more likely, limitations in the strength NAO – proxy calibration in the lake sediment record. There, the NAO-like pattern is found in the third principal component, which explains only 9.4% of the variability in the data. Only further long, high-resolution proxy records can help to develop improved NAO reconstructions.

## Discussion

Our 3000-year record confirms persistently low growth-rates, reflective of positive NAO states, during the MCA. Another persistently low growth period occurred at 290-550 CE, and wavelet analysis demonstrates that period of slow (or fast) growth are coincident with periods of relatively low (or high) magnitude of multi-decadal variability, indicating a potential relationship between the strength of the Atlantic Multidecadal Oscillation and the NAO at these times. The phase of persistent positive NAO that occurs at ~290-550 CE coincides with a period of increased climate variability and an unusually wet growing season (April-June) in central Europe as observed in tree rings^24^. We hypothesise that composite stalagmite and tree ring proxies are responsive to the same North Atlantic climate forcing, which leads to a persistently positive NAO.

Assuming that we can utilise the SU_comp_ record as a proxy for the NAO over the last 3000 years, this allows us to start building hypotheses relating to the impact of climate variability on environmental and societal change in NW Europe. For example, although we recognise that climatic changes alone are unlikely to be the sole driver for societal change^25^, it is noted that the positive NAO phase at ~290-550 CE is coincident with the decline of the Roman Empire and the European Migration period. This was followed by generally negative NAO conditions in the period 600-900 AD, which would provide suitable conditions for westward expansion in the north Atlantic region. In NW Europe, the timing coincides with the start of the Viking Age of expansion into northern and western Europe. The cause and precise timing of this is contested^26^,^27^, and our SU_comp_ record provides a useful climatic context of colder Scandinavian climate and weaker westerly circulation for this important phase of early European history.

## METHODS

### Lamina Counting

The fluorescent laminae for stalagmite SU963 were counted and their thicknesses measured in duplicate using techniques detailed previously^5^. No hiatuses were observed and a total of 1704 continuous laminae were counted, with a mean lamina thickness of 27.0 μm and a mean standard deviation between replicate measurements of 5.2 μm (n = 1704). The mean lamina thickness for all stalagmites reported in this paper is archived at the World Data Centre – Paleoclimatology.

### Timing of SU963 deposition

Stalagmite SU963 was dated with U-Th mass spectrometry using methods previously reported^28^. U-Th ages and isotope data are presented in Supplementary Table 1. The U-Th ages were used to constrain the floating lamina chronology to the U-Th ages. Following established methods^29^,^30^, the relative lamina number was subtracted from each U-Th age to obtain five determinations of the date of the end of deposition. This approach yielded a date for the end of deposition of 220 years before present (AD2010) (95% Confidence Interval: 143-315 years before present). We compared this date with that calculated from a linear regression of the U-Th age vs the relative lamina number (U-Th age = 288.5 + 0.9088 x lamina number; r = 0.99, n = 5). The slope of the regression (0.91, with a 95% confidence range of 0.71 to 1.10) is indistinguishable from zero, provided independent confirmation of the continuous, annual nature of lamina deposition. The intercept provided a date for the end of deposition of 289 years before present (95% Confidence Interval: 124-454 years before present). The U-Th based and lamina-based age-depth relationships are presented in Supplemental Figure S4.

### Correlation of growth-rate series

To further constrain the timing of deposition of SU963, the annual lamina thickness series was cross-correlated with that of stalagmites SU961, SU962 and SU967. Cross-correlations were calculated for theoretical end-of-deposition dates between 120 and 320 years before present. The strongest cross-correlations were observed when the period of divergent lamina thickness in SU963 of 150 years at ~1550 years before present was removed from the analysis: for SU961 with an end-of-deposition date of 210 year before present (r = 0.25); for SU962 with an end-of-deposition date of 229 years before present (r = 0.46); and for SU967 with an end-of-deposition date of 223 years before present (r = 0.34). The mean of these three approaches yields a date of growth cessation of 221 years before present. We have placed the SU963 lamina thickness series into the chronology with the end of deposition at 229 years before present, which is identical to the mean of the U-Th predicted date of the end of deposition and with the strongest cross-correlating stalagmite (SU962).

### Geostatistical methods

Bootstrapping of instrumental and composite series, variogram analysis, growth acceleration and wavelet analyses of the annual growth-rate series were performed in MATLAB, Wolfram Mathematica and Microcal Origin, and wavelet analyses visualised using the IDL Wavelet Toolkit^31^. Bootstrapping was carried out by generating 100,000 replicates of the data by sampling with replacement years of the instrumental period, each set of dates yielding a new time series for both NAO and SU_comp_. Correlation analyses were performed using Microcal Origin, SPSS, Excel and Climate Explorer^32^. To allow correlation analysis between proxy records of different temporal resolution, we smoothed the annual resolution SU_comp_ record using a cubic smoothing spline to the approximate resolution of the lower resolution proxies (5 years for the Morocco stalagmite^21^, 20 years for the West Greenland lake sediment record^17^, and 30 years for NAO_ms_^1^ and the Morocco tree-ring record^20^). To reduce the effects of autocorrelation, we sampled the smoothed time series every 30 years for NAO_ms_ and the Morocco tree-ring record^20^ or at the years when data points were recorded in the other proxies^17^,^21^. We then calculated Pearson’s correlation coefficients for time series consisting only of these sequential samples.

## Additional Information

**How to cite this article**: Baker, A. *et al*. A composite annual-resolution stalagmite record of North Atlantic climate over the last three millennia. *Sci. Rep.* **5**, 10307; doi: 10.1038/srep10307 (2015).

## Supplementary Material

Supplementary Information

## References

[b1] Fairchild I. J. & Baker A. Speleothem Science. Wiley, London (2012).

[b2] Charman D. J., Caseldine C. J., Baker A., Gearey B. & Hatton J. Palaeohydrological records from peat profiles and speleothems in Sutherland, NW Scotland. Quat. Res. 55, 223–234 (2001).

[b3] Hurrell J. W. Decadal trends in the North Atlantic Oscillation: regional temperatures and precipitation. Science 269, 676–679 (1995). 17758812 10.1126/science.269.5224.676

[b4] Baker A., Smart P. L., Edwards R. L. & Richards D. A. Annual banding in a cave stalagmite. Nature 364, 518–520 (1993).

[b5] Proctor C. J., Baker A., Barnes W. L. & Gilmour M. A. A thousand year speleothem proxy record of North Atlantic climate from Scotland. Clim. Dyn. 16, 815- 820 (2000).

[b6] Proctor C. J., Baker A. & Barnes W. L. A three thousand year record of north Atlantic climate. Clim. Dyn. 19, 449–454 (2002).

[b7] Trouet V. *et al.* Persistent positive North Atlantic Oscillation mode dominated the Medieval Climate Anomaly. Science 324, 78–80 (2009). 19342585 10.1126/science.1166349

[b8] Baker A., *et al.* High resolution δ^18^O and δ^13^C records from an annually laminated Scottish stalagmite and relationship with last millennium climate. Glob. Planet. Chang. 79, 303–311 (2011).

[b9] Shen C-C. *et al.* Testing the annual nature of speleothem banding. Sci. Rep. 3, 2633 (2013). 24037594 10.1038/srep02633 PMC3773624

[b10] Harris I., Jones P. D., Osborn T. J. & Lister D. H. Updated high-resolution grids of monthly climatic observations – the CRU TS3.10 Dataset. Int. J. Climatol. 34, 623–642 (2013).

[b11] Kalnay E. & coauthors, The NCEP/NCAR Reanalysis 40-year Project. B. Am. Meteorol. Soc. 77, 437–471 (1996).

[b12] Efron B. Bootstrap Methods: Another Look at the Jackknife. Ann. Stat. 7, 1–26 (1979).

[b13] Esper J., Cook E. R., Krusic P. J., Peters K. & Schweingruber F. H. Tests of the RCS method for preserving low-frequency variability in long tree-ring chronologies. Tree-Ring Res. 59, 81–98 (2003).

[b14] Mariethoz G., Kelly B. & Baker A. Quantifying the value of laminated stalagmites for paleoclimate reconstructions. Geophys. Res. Lett. 39, L05407 (2012).

[b15] Baker A., Bradley C. & Phipps S. J. Hydrological modelling of stalagmite δ^18^O response to glacial-interglacial transitions. Geophys. Res. Lett. 40, 1–6 (2013).

[b16] Dreybrodt W. Chemical kinetics, speleothem growth and climate. Boreas 28, 347- (1999).

[b17] Olsen J., Anderson N. J. & Knudsen M. F. Variability of the North Atlantic Oscillation over the past 5,200 years. Nat. Geosci. 5, 808–812 (2012).

[b18] Ólafsdóttir K., Geirsdottir A., Miller G. H., & Larsen D. J. Evolution of NAO and AMO strength and cyclicity from a 3-ka varve-thickness record from Iceland. Quat. Sci. Rev. 69, 142–154 (2013).

[b19] Knudsen M. F., Jacobsen B. H., Seidenkrantz M-S. & Olsen J. Evidence for external forcing of the Atlantic Multidecadal Oscillation since termination of the Little Ice Age. Nat. Commun. 5, 3323 (2014). 24567051 10.1038/ncomms4323 PMC3948066

[b20] Esper J., Frank D., Buntgen U., Verstege A. & Luterbacher J. Long-term drought severity variations in Morocco. Geophys. Res. Lett., 34, L17702 (2007).

[b21] Wassenburg J. A. *et al.* Moroccan Speleothem and tree ring records suggest a variable positive state of the North Atlantic Oscillation during the Medieval Warm Period. Earth Planet. Sci. Lett. 375, 291–302 (2013).

[b22] Rutlidge H. *et al.* Dripwater organic matter and trace element geochemistry in a semi-arid karst environment: implications for speleothem paleoclimatology. Geochim. Cosmochim. Acta 135, 217–230 (2014).

[b23] Ahmed M. *et al.* Continental-scale temperature variability during the past two millennia. Nat. Geosci. 6, 339–346 (2013).

[b24] Büntgen U *et al.* 2500 Years of European climate variability and human susceptibility. Science 331, 578–582 (2011). 21233349 10.1126/science.1197175

[b25] Diaz H. & Trouet V. Some perspectives on societal impacts of past climatic changes. History Compass 12, 160–177 (2014).

[b26] Barrett J. H. What caused the Viking Age? Antiquity 82, 671–685 (2008).

[b27] von Holstein I. C. C. *et al.* Searching for Scandinadians in pre-Viking Scotland: molecular fingerprinting of Early Medieval combs. J. Archaeol. Sci. 41, 1–6 (2014)

[b28] Hellstrom J. Rapid and accurate U/Th dating using parallel ion-counting multi-collector ICP-MS. J. Anal. At. Spectrom. 18, 1346–1351 (2003).

[b29] Asrat A., *et al.* A high-resolution multi-proxy stalagmite record from Mechara, Southeastern Ethiopia: palaeohydrological implications for speleothem palaeoclimate reconstruction. J. Quat. Sci. 22, 53–63 (2007).

[b30] Dominguez-Villar D., Baker A., Fairchild I. J. & Edwards R. L. A method to anchor floating chronologies in annually laminated speleothems with U-Th dates. Quat. Geochronol. 14, 57–66 (2012).

[b31] Torrence C. & Compo G. P. A practical guide to Wavelet Analysis. B. Am. Meteorol. Soc. 79, 61–78 (1998).

[b32] Trouet V. & Van Oldenborgh G. J. KNMI Climate Explorer: A web-based research tool for high-resolution paleoclimatology. Tree-Ring Res. 69, 3–13 (2013).

