# Supplementary material for: A composite annual-resolution stalagmite record of North Atlantic climate over the last three millennia
Source: Sci Rep. 2015 Jun 11;5:10307. doi: 10.1038/srep10307 (PMC4464153; doi:10.1038/srep10307)
Supplement: Supplementary Information [file srep10307-s1.pdf]

A composite annual-resolution stalagmite record of North Atlantic climate over the last three millennia

Andy Baker[1 *], Jon Hellstrom[2], Bryce F.J. Kelly[1], Gregoire Mariethoz[3], Valerie Trouet[4]

[1]Connected Waters Initiative Research Centre, UNSW Australia, Sydney, NSW 2052, Australia.

[2]School of Earth Sciences, The University of Melbourne, Melbourne, VIC 3010, Australia.

[3]University of Lausanne, Institute of Earth Surface Dynamics (IDYST), UNIL-Mouline, Geopolis, 1015 Lausanne, Switzerland.

[4]Laboratory of Tree-Ring Research, University of Arizona, Tucson AZ 85721, USA.

*Corresponding author: a.baker@unsw.edu.au

Supplementary Table 1 . U-Th data for stalagmite SU963. All uncertainties are quoted at 2 standard error. Corrected ages use a [^{230}Th/^{232}Th] of 0.6±0.5. All ages are reported as ka before present, where present is 2010 AD.

Sample ID	Distance from top (mm)	Lamina number (mean and range covered by U-Th sample)	Mass (g)	^{238}U (ng/g)	[^{230}Th] / [^{238}U]	[^{234}U] / [^{238}U]	Age Ka	[^{232}Th] / [^{238}U]	[^{230}Th] / [^{232}Th]	Corrected Age Ka	[^{234}U] / [^{238}U]i Corr
SU963-5	41.0	1513 (1429-1575)	0.0345	260±20	0.0205 ±0.0007	1.2689 ±0.0036	1.770 ±0.064	0.001484 ±0.000015	13.8	1.584 ±0.201	1.2702 ±0.0036
SU963-4	31.0	1142 (1093-1188)	0.0436	241±18	0.0152 ±0.0004	1.2460 ±0.0027	1.330 ±0.033	0.000271 ±0.00005	55.8	1.305 ±0.050	1.2469 ±0.0027
SU963-3	22.0	856 (800-901)	0.0414	232±40	0.0127 ±0.0007	1.2058 ±0.0031	1.151 ±0.062	0.000514 ±0.000107	24.7	1.085 ±0.095	1.2064 ±0.0031
SU963-2	14.0	379 (322-481)	0.0329	244±18	0.0064 ±0.0005	1.2231 ±0.0027	0.566 ±0.041	0.000278 ±.000006	22.9	0.535 ±0.059	1.2235 ±0.0027
SU963-1	3.0	104 (55-163)	0.0335	266±20	0.0052 ±0.0005	1.2560 ±0.0036	0.453 ±0.046	0.000362 ±0.000006	14.4	0.406 ±0.065	1.2563 ±0.0037

Supplemental Table 2. Comparison of reconstructed NAO series. Table presents pearson correlation coefficients over the period 1049-1634 CE for sequential non-overlapping segments of the SU_{comp} record (this paper), Moroccan tree rings [20], a Moroccan stalagmite trace element record [21], and West Greenland lake sediment record [17]. The Moroccan stalagmite [20] and Greenland lake [17] time series are not continuous and were smoothed using a cubic spline that reflected their average sampling interval (five years for ref [20], twenty years for ref [17]). The SU_{comp} and tree-ring [20] time series were smoothed with 5 and 20 year cubic splines for comparison to ref [21] and ref [17] time series. Note that the Greenland lake record chronology was constructed based on a tuning to the NAO_{ms} [1] which in part explains the strong correlation.

	smoothing	**SU_{comp}**		**Morocco tree ring**		**Morocco stalagmite**
smoothing		*5*	*20*	*5*	*20*	
**Morocco Tree ring**	*5*	0.48				
	20		0.55			
**Morocco stalagmite**		0.65		0.28		
**Greenland lake**			-0.75		-0.55	-0.57

Supplementary Figure 1. SU963 fluorescent lamina. The figure presents three overlapping microscope images taken using a Zeiss Axiotech microscope with ‘blue’ filter set and x10 magnification. The location of annual fluorescence laminae are hand drawn, and the lamina year is from the chronology described in Methods. The images show the last ~46 years of stalagmite deposition. Each image has dimensions of 527 x 703 μm, with a pixel resolution of 0.34 μm.

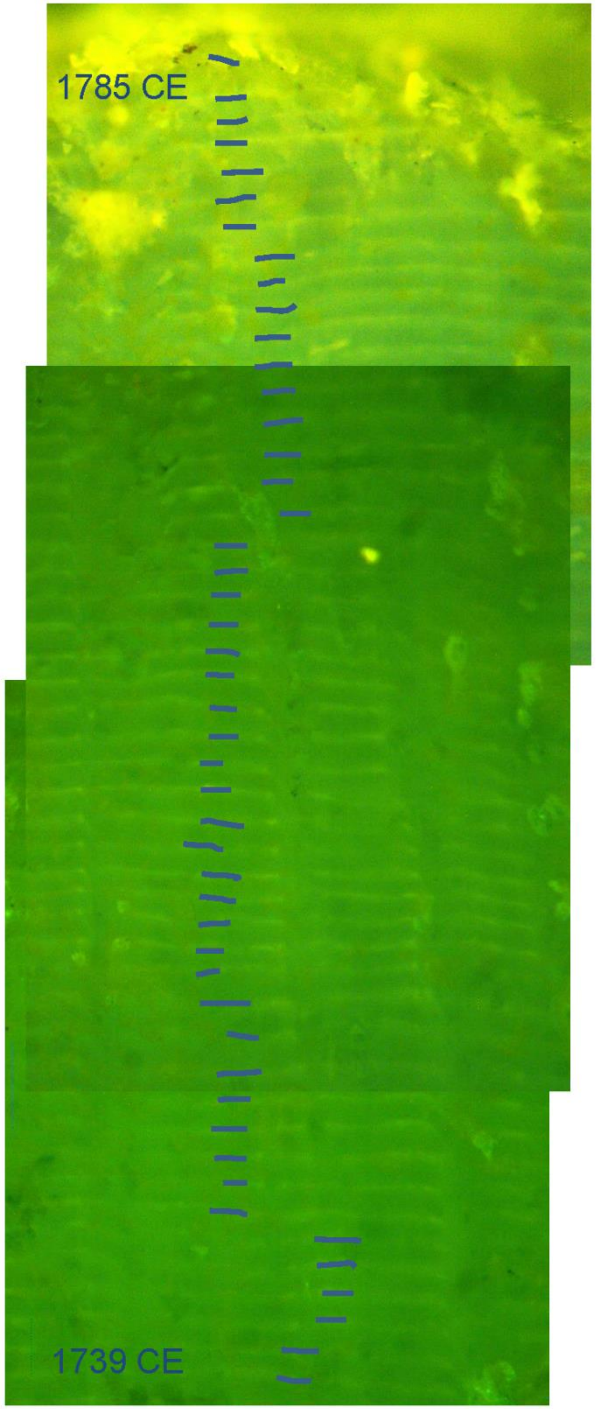

Supplementary Figure 2. Pearson correlation coefficients between SU_{comp} series and seasonal precipitation (P), temperature (T), and the Stykkisholmur-Azores NAO time series. P and T were calculated from the CRU TS3.22 data set as averages over the area 55-60 °N and 0-10 °W. All correlation coefficients were calculated over the period 1901-2004. Seasons were defined as January-March (JFM), April-June (AMJ), July-September (JAS), and October-December (OND).

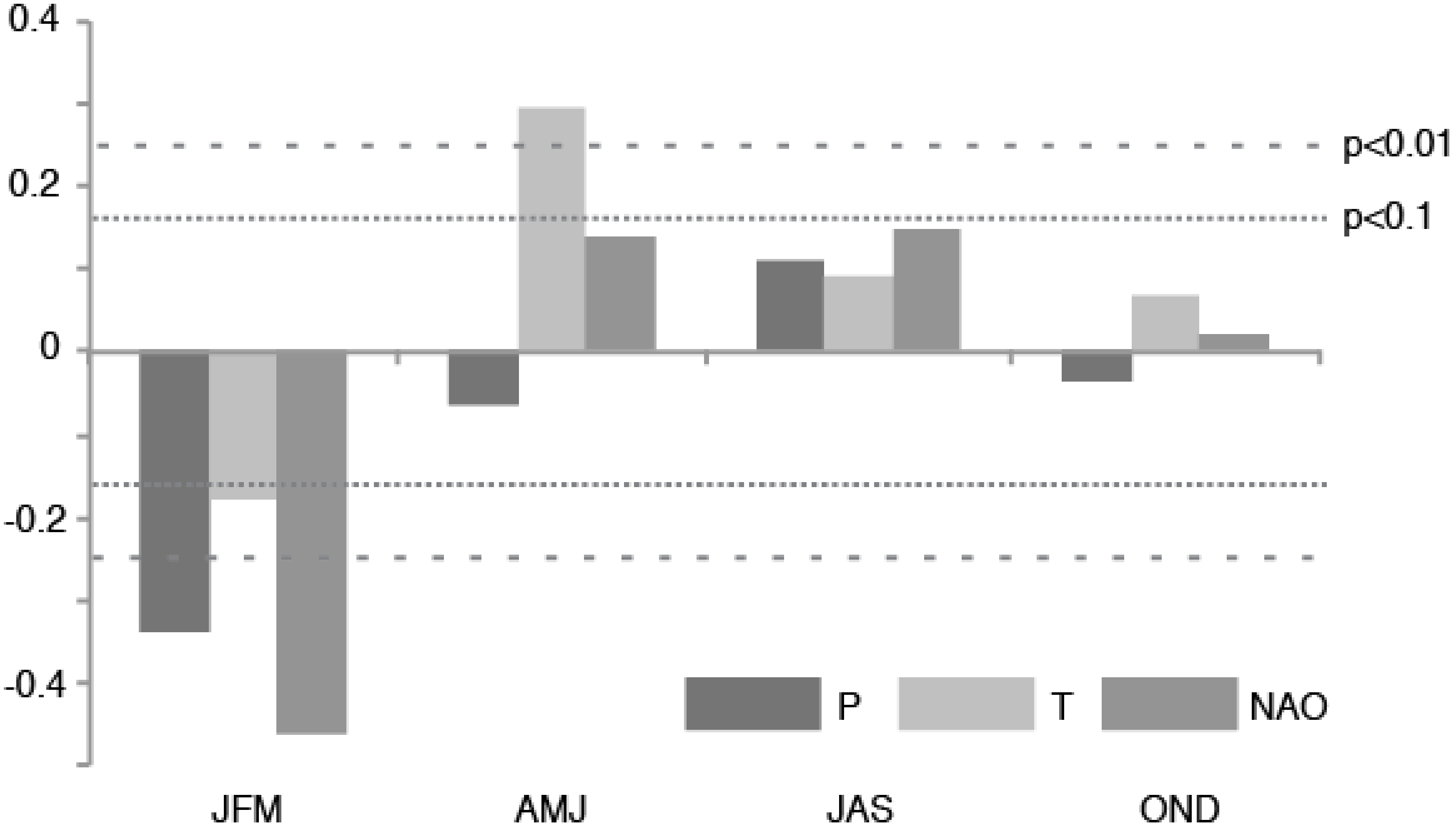

Supplementary Figure 3. Wavelet analysis of the five stalagmite growth rate series. For each stalagmite are shown (a) Normalised growth rate data. (b) The wavelet power spectrum. The *x*-axis shows the time in years before present, where present is 2010 AD, and the top of the sample (youngest) is to the left. The contour levels are chosen so that 75%, 50%, 25%, and 5% of the wavelet power is above each level, respectively. The solid black line is the 99% significance level when compared to white noise. The cone of influence is shown by hashed lines. (c) The global wavelet power spectrum. A Morlet filter is used in all cases, and missing data are replaced with zeros. The wavelet software was provided by C. Torrence and G. Compo, and is available at http://paos.colorado.edu/research/wavelets

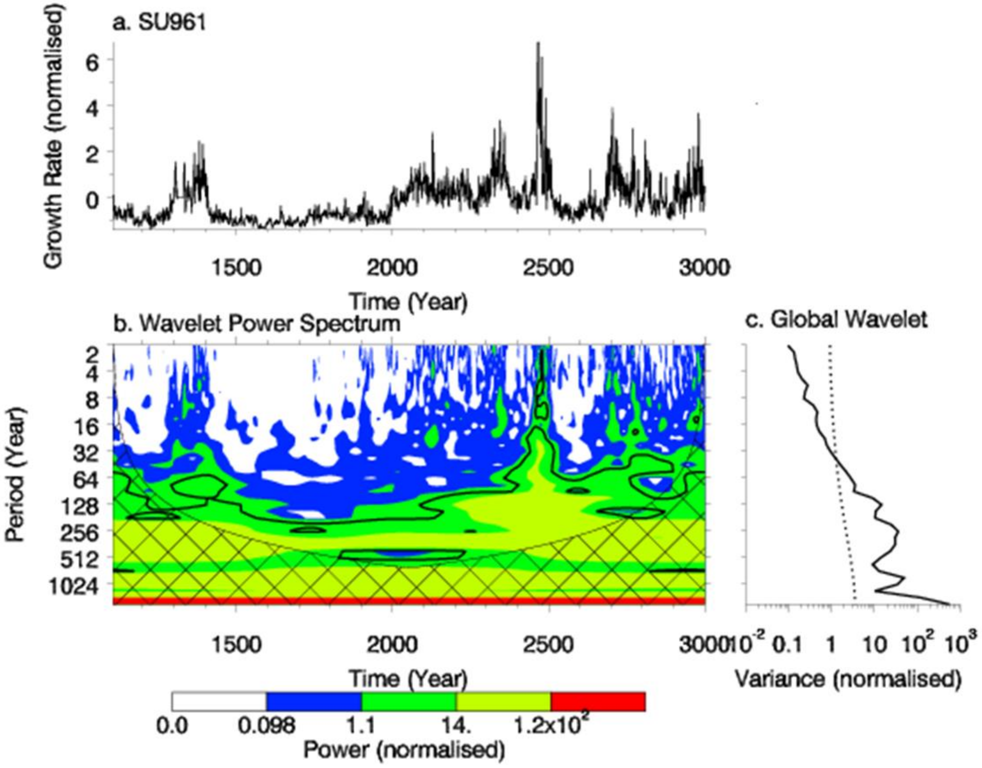
a. SU961
Growth Rate (normalised)
Time (Year)
b. Wavelet Power Spectrum
Period (Year)
c. Global Wavelet
Variance (normalised)
0.0 0.098 1.1 14. 1.2x10²
Power (normalised)

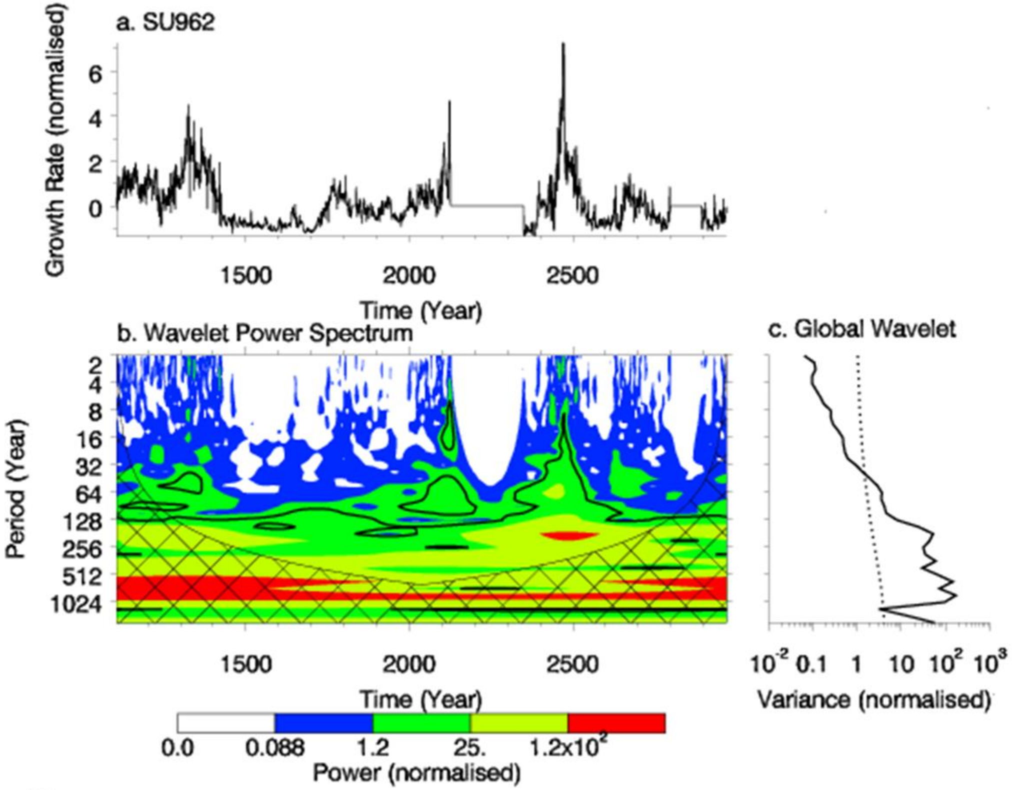
a. SU962
Growth Rate (normalised)
Time (Year)
b. Wavelet Power Spectrum
Period (Year)
c. Global Wavelet
Variance (normalised)
0.0 0.088 1.2 25. 1.2x10²
Power (normalised)

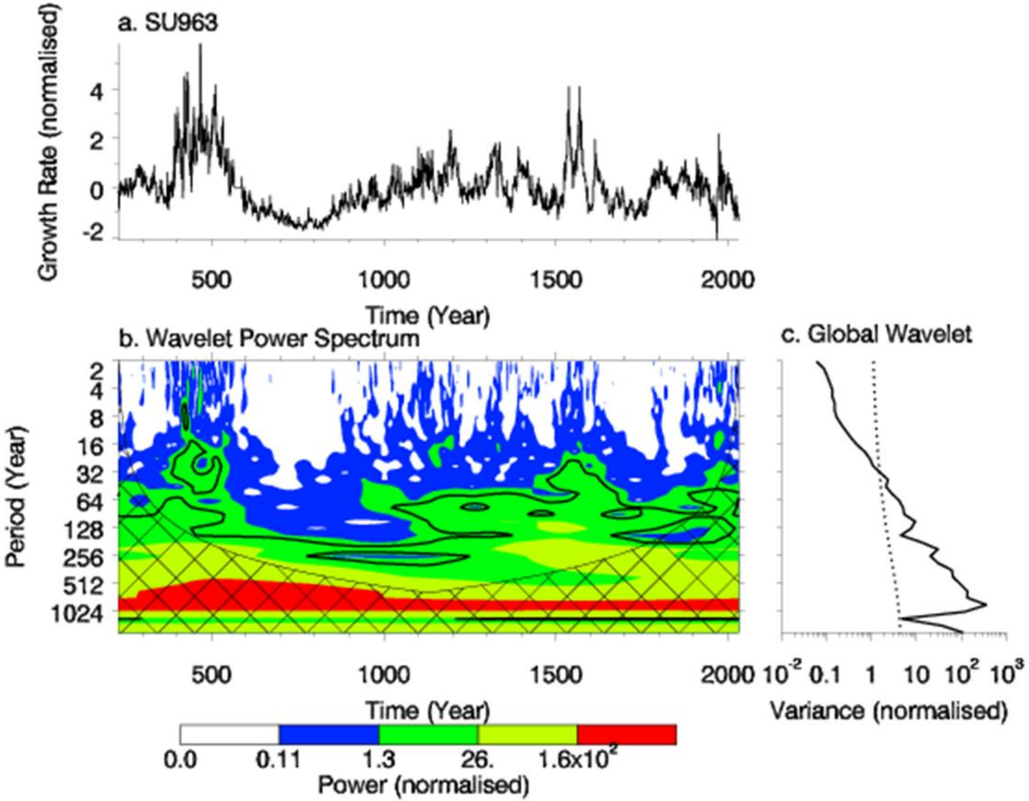
a. SU963
Growth Rate (normalised)
Time (Year)
b. Wavelet Power Spectrum
Period (Year)
c. Global Wavelet
Variance (normalised)
0.0 0.11 1.3 26. 1.6x10²
Power (normalised)

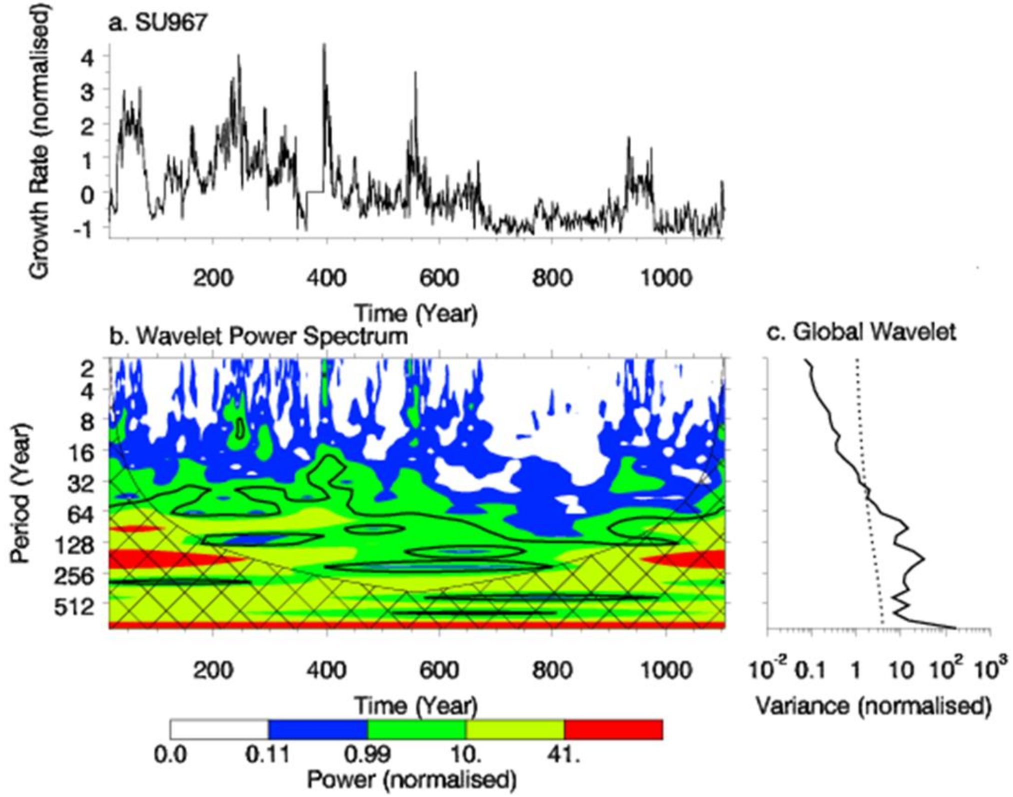
a. SU967
Growth Rate (normalised)
Time (Year)
b. Wavelet Power Spectrum
Period (Year)
c. Global Wavelet
Variance (normalised)
0.0 0.11 0.99 10. 41.
Power (normalised)

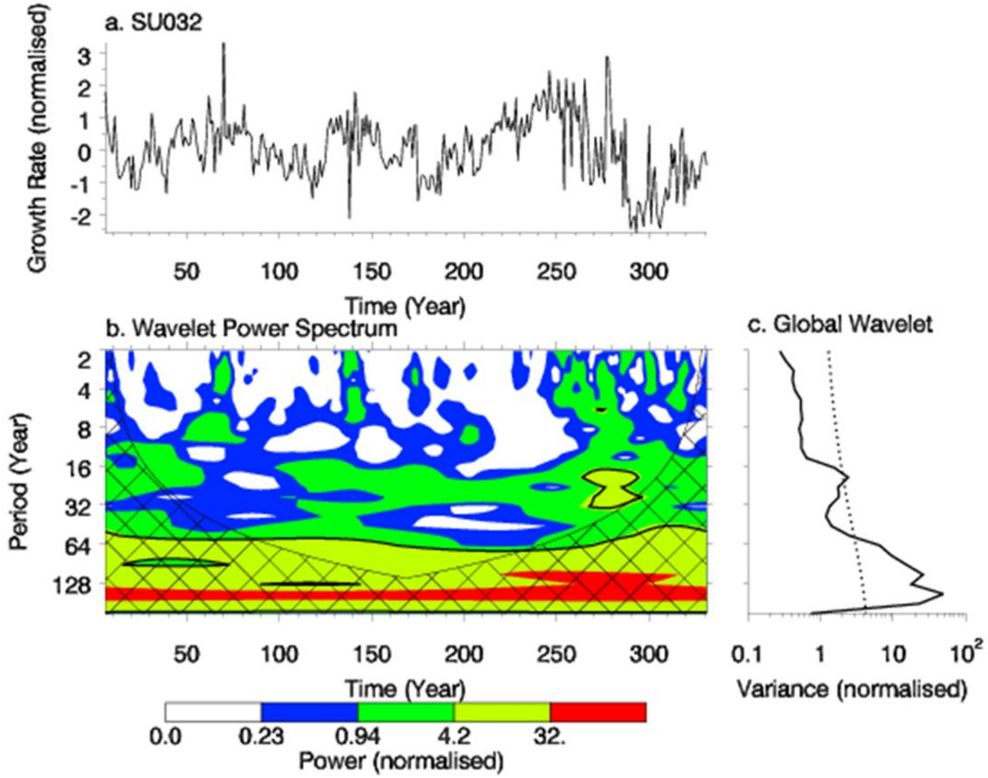
a. SU032
Growth Rate (normalised)
3
2
1
0
-1
-2
50
100
150
200
250
300
Time (Year)
b. Wavelet Power Spectrum
Period (Year)
2
4
8
16
32
64
128
50
100
150
200
250
300
Time (Year)
0.0
0.23
0.94
4.2
32.
Power (normalised)
c. Global Wavelet
0.1
1
10
10^2
Variance (normalised)

Supplemental Figure 4. Comparison of age-depth relationships. Red squares are U-Th ages (from Supplemental Table 1) with uncertainties derived from analytical uncertainties (x-axis) and thickness of stalagmite sampled (y-axis). Solid line is the annual lamina chronology, using a final year of deposition of 229 year BP (see Methods for further details).

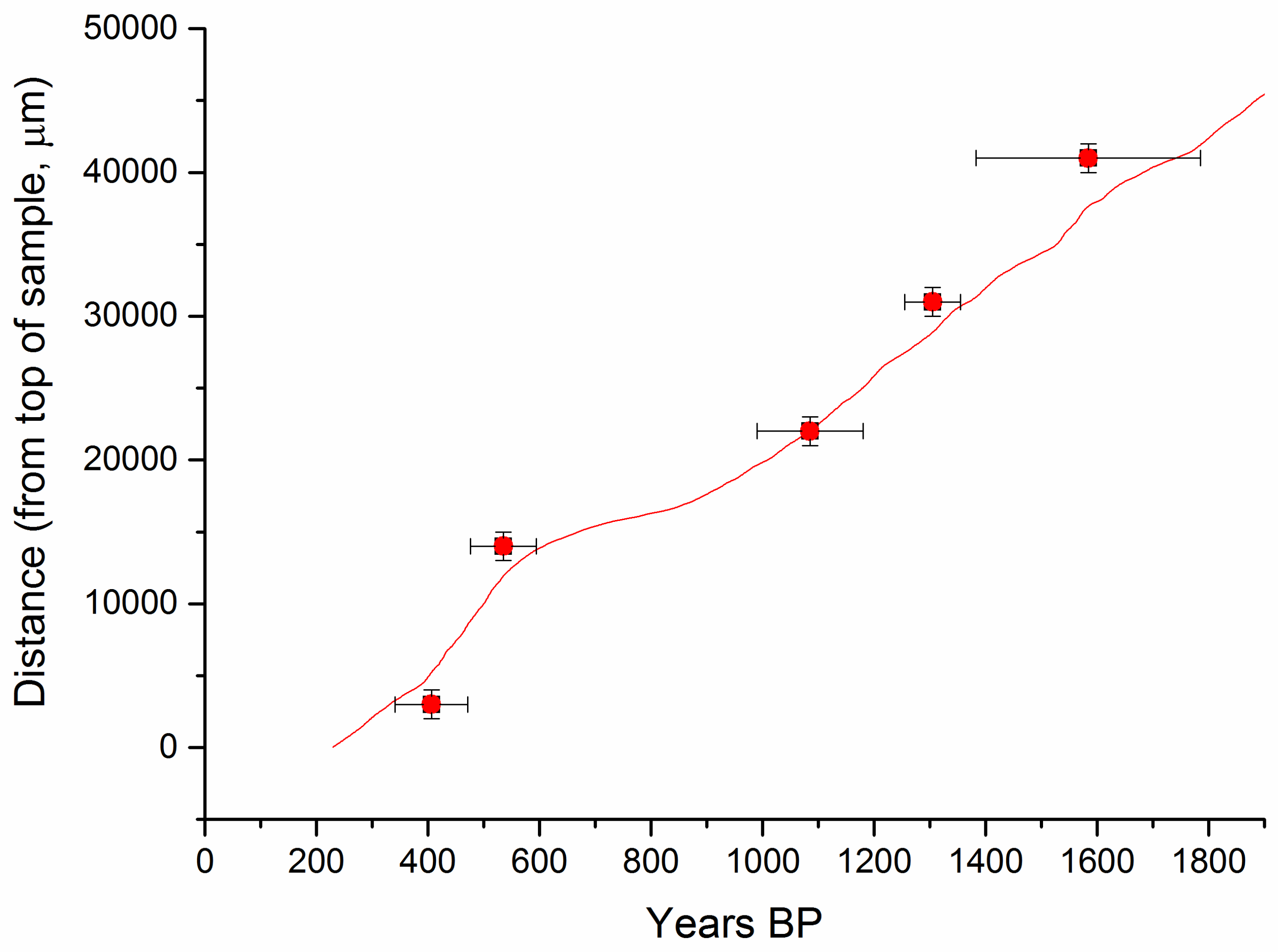

Supplemental Data.

Normalised growth rates are each stalagmite, and the averaged composite growth rate record.

Date		Individual Stalagmite Growth Rate Series	Individual Stalagmite Growth Rate Series	Individual Stalagmite Growth Rate Series	Individual Stalagmite Growth Rate Series	Individual Stalagmite Growth Rate Series	SU Composite Growth Rate series
		SU967	SU961	SU962	SU963	SU032	
Years before 2010 CE	CE / BCE	normalised	normalised	normalised	normalised	normalised	
5	2005						
6	2004					1.8	1.8
7	2003					0.76	0.76
8	2002					0.46	0.46
9	2001					0.06	0.06
10	2000					-0.11	-0.11
11	1999					1.06	1.06
12	1998					0.03	0.03
13	1997					-0.84	-0.84
14	1996					-0.89	-0.89
15	1995	-0.81				-0.78	-0.8
16	1994	-0.87				-0.62	-0.74
17	1993	-0.32				-0.39	-0.36
18	1992	-0.38				-0.3	-0.34
19	1991	-0.11				-0.21	-0.16
20	1990	0.11				-1.2	-0.54
21	1989	-0.38				-0.18	-0.28
22	1988	-0.38				-1.23	-0.8
23	1987	-0.49				-1.23	-0.86
24	1986	-0.43				-1.2	-0.81
25	1985	-0.65				-0.63	-0.64
26	1984	-0.54				-0.5	-0.52
27	1983	-0.49				-0.18	-0.33
28	1982	-0.59				0.29	-0.15

29	1981	-0.54	0.15	-0.19
30	1980	0.87	-0.4	0.23
31	1979	1.2	1.15	1.17
32	1978	1.47	0.75	1.11
33	1977	1.69	-0.1	0.79
34	1976	1.74	-0.5	0.62
35	1975	1.41	0.12	0.77
36	1974	2.12	-0.26	0.93
37	1973	1.58	-0.77	0.4
38	1972	0.71	-0.79	-0.04
39	1971	1.31	-1.36	-0.03
40	1970	1.31	-0.17	0.57
41	1969	2.77	0.13	1.45
42	1968	2.99	0.37	1.68
43	1967	2.39	0.04	1.21
44	1966	2.34	0.54	1.44
45	1965	1.96	0.73	1.35
46	1964	1.63	0.8	1.21
47	1963	1.85	0.99	1.42
48	1962	2.12	0.12	1.12
49	1961	2.39	0.41	1.4
50	1960	2.17	0.28	1.23
51	1959	1.63	0.15	0.89
52	1958	1.69	0.22	0.95
53	1957	2.12	0.98	1.55
54	1956	1.69	0.82	1.25
55	1955	2.66	0.36	1.51
56	1954	2.23	-0.04	1.09
57	1953	1.96	-0.34	0.81
58	1952	2.07	-0.13	0.97
59	1951	2.45	-0.71	0.87
60	1950	1.85	-0.29	0.78
61	1949	1.52	0.76	1.14
62	1948	1.96	1.69	1.82

63	1947	1.96	0.82	1.39
64	1946	1.69	0.92	1.3
65	1945	2.12	-0.71	0.71
66	1944	1.52	0.5	1.01
67	1943	1.09	0.71	0.9
68	1942	2.34	-0.15	1.1
69	1941	2.39	0.49	1.44
70	1940	3.1	3.35	3.22
71	1939	1.9	0.41	1.16
72	1938	1.41	0.34	0.88
73	1937	1.9	0.82	1.36
74	1936	1.74	0.27	1
75	1935	1.52	0.15	0.84
76	1934	1.25	0.87	1.06
77	1933	0.76	0.47	0.62
78	1932	0.49	0.78	0.64
79	1931	0.55	0.49	0.52
80	1930	0.65	0.64	0.65
81	1929	0.65	1.42	1.04
82	1928	0.44	0.46	0.45
83	1927	0.06	0.57	0.31
84	1926	0	0.5	0.25
85	1925	-0.43	0.41	-0.01
86	1924	-0.27	-0.78	-0.52
87	1923	-0.49	-0.3	-0.39
88	1922	-0.65	-0.36	-0.5
89	1921	-0.59	-0.47	-0.53
90	1920	-0.65	0.4	-0.12
91	1919	-0.65	0.5	-0.07
92	1918	-0.81	0.08	-0.36
93	1917	-0.7	0.05	-0.33
94	1916	-0.65	-0.65	-0.65
95	1915	-0.65	0.05	-0.3
96	1914	-0.76	0.05	-0.35

97	1913	-0.65	0.21	-0.22
98	1912	-0.65	-0.24	-0.44
99	1911	-0.49	-0.17	-0.33
100	1910	-0.21	-0.46	-0.34
101	1909	-0.16	0.02	-0.07
102	1908	-0.21	-0.45	-0.33
103	1907	-0.16	-0.85	-0.5
104	1906	-0.27	-0.94	-0.6
105	1905	-0.49	-0.78	-0.63
106	1904	-0.43	-0.4	-0.42
107	1903	-0.49	-0.69	-0.59
108	1902	-0.49	0.11	-0.19
109	1901	-0.38	0.19	-0.1
110	1900	-0.65	-0.34	-0.49
111	1899	-0.54	-0.79	-0.66
112	1898	-0.32	-0.9	-0.61
113	1897	-0.16	-0.25	-0.2
114	1896	-0.21	0.2	-0.01
115	1895	0.55	-0.63	-0.04
116	1894	0.44	-0.82	-0.19
117	1893	0.33	-1.01	-0.34
118	1892	0.44	-1.52	-0.54
119	1891	0.49	-0.49	0
120	1890	1.09	-1.17	-0.04
121	1889	0.87	-0.98	-0.05
122	1888	0.6	-0.31	0.14
123	1887	0.76	-0.85	-0.04
124	1886	0.38	-0.53	-0.08
125	1885	0.38	-0.34	0.02
126	1884	0.17	0.58	0.37
127	1883	-0.05	0.8	0.37
128	1882	0.27	0.99	0.63
129	1881	0.49	0.51	0.5
130	1880	1.03	0.56	0.8

131	1879	0.76	1.02	0.89
132	1878	0.49	0.58	0.54
133	1877	0.6	1.09	0.84
134	1876	0.44	0.74	0.59
135	1875	0.76	0.18	0.47
136	1874	0	0.24	0.12
137	1873	0.38	1.26	0.82
138	1872	0.44	-2.15	-0.86
139	1871	0.49	0.9	0.69
140	1870	0.44	-0.03	0.21
141	1869	0.22	1.82	1.02
142	1868	0.11	1.25	0.68
143	1867	0.93	-0.5	0.21
144	1866	0.33	0.04	0.18
145	1865	-0.81	0.99	0.09
146	1864	-0.21	0.42	0.1
147	1863	-0.27	1.1	0.42
148	1862	-0.16	0.48	0.16
149	1861	0.11	0.93	0.52
150	1860	-0.05	0.23	0.09
151	1859	0.06	0.13	0.1
152	1858	0.11	0.23	0.17
153	1857	0.38	0.87	0.63
154	1856	0.17	0.55	0.36
155	1855	0.17	-0.21	-0.02
156	1854	0.38	-0.29	0.04
157	1853	0.71	-0.47	0.12
158	1852	0.98	-0.6	0.19
159	1851	1.03	-0.44	0.3
160	1850	1.63	-0.25	0.69
161	1849	1.85	-0.44	0.7
162	1848	1.96	-0.15	0.9
163	1847	0.82	-0.21	0.3
164	1846	1.9	-0.25	0.83

165	1845	1.63	-0.5	0.56
166	1844	0.71	-0.5	0.1
167	1843	0.6	0.43	0.51
168	1842	0.87	0.22	0.54
169	1841	0.76	0.1	0.43
170	1840	1.2	0.6	0.9
171	1839	0.71	0.71	0.71
172	1838	0.44	-0.25	0.09
173	1837	0.65	0.77	0.71
174	1836	0.49	0.77	0.63
175	1835	0.22	-1.6	-0.69
176	1834	0.38	-1.18	-0.4
177	1833	0.33	-0.8	-0.23
178	1832	0.27	-0.8	-0.26
179	1831	-0.05	-0.89	-0.47
180	1830	0.17	-1.18	-0.51
181	1829	0.27	-1.25	-0.49
182	1828	0.33	-0.8	-0.24
183	1827	-0.05	-0.82	-0.43
184	1826	0.44	-1.14	-0.35
185	1825	0.17	-1.36	-0.6
186	1824	-0.05	-0.36	-0.21
187	1823	0.22	-1.41	-0.6
188	1822	0.17	-0.46	-0.14
189	1821	0.17	0.5	0.33
190	1820	0.27	-0.17	0.05
191	1819	0.6	-0.61	-0.01
192	1818	0.49	0.06	0.27
193	1817	0.65	-0.29	0.18
194	1816	0.93	0.06	0.49
195	1815	0.49	0.22	0.35
196	1814	0.87	-0.13	0.37
197	1813	0.65	-0.8	-0.07
198	1812	0.27	-0.46	-0.09

199	1811	0.17		-0.72	-0.28
200	1810	0.06		0.1	0.08
201	1809	0.33		0.77	0.55
202	1808	0.55		0.36	0.45
203	1807	1.36		-0.06	0.65
204	1806	0.82		0.48	0.65
205	1805	0.65		-0.79	-0.07
206	1804	1.69		-0.76	0.46
207	1803	1.96		-0.56	0.7
208	1802	1.52		-0.63	0.45
209	1801	1.63		-0.39	0.62
210	1800	1.69		0.39	1.04
211	1799	1.31		0.17	0.74
212	1798	1.41		0.48	0.95
213	1797	1.31		0.39	0.85
214	1796	1.14		-0.15	0.5
215	1795	1.69		0.67	1.18
216	1794	1.74		0.52	1.13
217	1793	1.96		0.85	1.4
218	1792	2.01		0.77	1.39
219	1791	1.31		0.9	1.1
220	1790	1.31		0.61	0.96
221	1789	0.65		1.12	0.89
222	1788	1.79		1.5	1.65
223	1787	2.07		0.68	1.37
224	1786	2.17		1.03	1.6
225	1785	1.31		0.58	0.94
226	1784	0.98		0.9	0.94
227	1783	0.87		0.61	0.74
228	1782	1.25		1.63	1.44
229	1781	1.63	-0.12	-0.34	0.39
230	1780	2.28	-0.32	0.58	0.85
231	1779	3.26	-0.9	1.01	1.12
232	1778	2.94	-0.4	0.43	0.99

233	1777	1.79	0.15	0.42	0.79
234	1776	1.74	0.15	1.12	1.01
235	1775	1.36	-0.04	0.54	0.62
236	1774	3.37	0.27	0.78	1.47
237	1773	2.17	-0.32	1.45	1.1
238	1772	2.72	-0.28	1.38	1.28
239	1771	1.31	0.15	1.73	1.06
240	1770	1.31	-0.02	1.25	0.85
241	1769	1.52	0.09	1.22	0.95
242	1768	1.69	-0.16	1.58	1.03
243	1767	2.01	0.02	1.89	1.31
244	1766	2.61	-0.08	1.63	1.38
245	1765	4.02	0.04	0.91	1.66
246	1764	3.21	-0.32	2.48	1.79
247	1763	3.64	0	1.74	1.79
248	1762	2.99	-0.12	1.2	1.36
249	1761	1.31	0.31	1.17	0.93
250	1760	0.49	-0.3	1.39	0.53
251	1759	0.06	0.39	2.19	0.88
252	1758	1.85	0.23	0.96	1.01
253	1757	2.5	0.11	1.32	1.31
254	1756	1.9	-0.16	-1.25	0.16
255	1755	1.63	0.15	2.24	1.34
256	1754	1.96	0	1.33	1.09
257	1753	1.47	-0.16	1.56	0.96
258	1752	2.07	-0.36	-0.52	0.4
259	1751	0.55	-0.38	2.11	0.76
260	1750	1.52	-0.08	1.29	0.91
261	1749	0.33	-0.16	0.88	0.35
262	1748	0.65	0.39	1.4	0.82
263	1747	0.55	0.31	0.2	0.35
264	1746	0.44	0.35	-0.98	-0.06
265	1745	0.55	0.39	2.22	1.05
266	1744	1.03	-0.08	1.3	0.75

267	1743	0.71	0.04	0.55	0.43
268	1742	0.71	0	-1.09	-0.13
269	1741	0.82	-0.43	-1.09	-0.23
270	1740	0.38	0.04	-0.86	-0.15
271	1739	0.87	0.21	0.42	0.5
272	1738	1.2	-0.04	-0.28	0.29
273	1737	1.36	0.17	-0.86	0.22
274	1736	0.6	0.02	0.42	0.35
275	1735	0.71	-0.08	-0.83	-0.07
276	1734	0.65	0.31	-1.05	-0.03
277	1733	1.2	0.19	2.92	1.44
278	1732	0.71	0.07	2.86	1.21
279	1731	1.25	0.04	1.7	1
280	1730	1.52	0.5	-0.41	0.54
281	1729	1.41	0.19	0.65	0.75
282	1728	0.55	0.78	-0.45	0.29
283	1727	0.71	0.62	-0.16	0.39
284	1726	0.44	0.78	-0.36	0.29
285	1725	0.44	0.43	0.57	0.48
286	1724	1.09	0.66	-1.78	-0.01
287	1723	1.25	0.54	0.99	0.93
288	1722	1.09	0.95	-1.64	0.14
289	1721	1.31	0.39	-1.51	0.06
290	1720	2.5	0.54	-2.47	0.19
291	1719	1.69	0.41	-2.18	-0.03
292	1718	2.45	0.9	-2.05	0.43
293	1717	1.58	0.78	-2.59	-0.08
294	1716	1.14	0.74	-1.6	0.09
295	1715	0.49	0.62	-1.7	-0.2
296	1714	-0.54	0.5	-2.05	-0.7
297	1713	0.82	0.04	-2.4	-0.52
298	1712	0.22	0.82	-1.7	-0.22
299	1711	0.17	0.41	-1.54	-0.32
300	1710	0.33	0.52	0.76	0.54

301	1709	0.65	0.17	-2.3	-0.49
302	1708	0.17	0	-1.03	-0.29
303	1707	0.33	0.66	-0.52	0.16
304	1706	0.22	0.64	-1.89	-0.34
305	1705	0.49	0.21	-2.26	-0.52
306	1704	0.44	0.43	-2.45	-0.53
307	1703	0.44	0.31	-1.84	-0.37
308	1702	0.27	0.11	-1.68	-0.43
309	1701	0.33	0.11	-1.17	-0.24
310	1700	0.71	0.47	-1.57	-0.13
311	1699	0.17	-0.16	-1.41	-0.47
312	1698	0.22	0.04	0.19	0.15
313	1697	0.71	0.11	-0.58	0.08
314	1696	0.22	-0.12	0	0.03
315	1695	0.27	0.05	-1.67	-0.45
316	1694	0.65	-0.18	-1.09	-0.21
317	1693	1.14	-0.08	0.35	0.47
318	1692	0.76	-0.3	0.69	0.38
319	1691	1.63	-0.08	-1.74	-0.06
320	1690	0.27	-0.28	-0.14	-0.05
321	1689	0.76	0.04	-1.24	-0.15
322	1688	0.76	0.11	-1.03	-0.05
323	1687	1.63	-0.08	-0.33	0.41
324	1686	1.25	0.43	-0.58	0.37
325	1685	0.87	0.15	-1.22	-0.07
326	1684	0.71	0.19	-0.77	0.04
327	1683	1.96	0.39	-0.96	0.46
328	1682	0.98	-0.08	-0.39	0.17
329	1681	1.31	0.43	-0.13	0.53
330	1680	0.98	0.64	-0.07	0.52
331	1679	1.09	0.23	-0.48	0.28
332	1678	1.25	0.11		0.68
333	1677	1.14	0.9		1.02
334	1676	0.27	0.27		0.27

335	1675	0.98	0.23	0.61
336	1674	0.65	0.27	0.46
337	1673	0.82	-0.04	0.39
338	1672	1.03	0.25	0.64
339	1671	0.82	-0.08	0.37
340	1670	0.6	-0.24	0.18
341	1669	0.44	-0.63	-0.1
342	1668	1.14	-0.51	0.32
343	1667	-0.21	-0.36	-0.29
344	1666	0.55	-0.51	0.02
345	1665	1.63	-0.36	0.64
346	1664	0.6	-0.63	-0.01
347	1663	0.17	-0.59	-0.21
348	1662	-0.05	0.04	-0.01
349	1661	-1.08	-0.08	-0.58
350	1660	-0.05	0.07	0.01
351	1659	0	-0.02	-0.01
352	1658	-0.38	0.31	-0.03
353	1657	-0.49	0.31	-0.09
354	1656	-0.32	0.27	-0.03
355	1655	-0.76	-0.28	-0.52
356	1654	-0.43	-0.04	-0.24
357	1653	-0.65	-0.04	-0.35
358	1652	-0.76	-0.2	-0.48
359	1651	-0.54	-0.4	-0.47
360	1650	-0.65	-0.67	-0.66
361	1649	-0.81	-0.34	-0.57
362	1648	-0.87	-0.32	-0.59
363	1647	-0.97	-0.47	-0.72
364	1646	-1.14	-0.47	-0.81
365	1645	--	-0.63	-0.63
366	1644	--	-0.38	-0.38
367	1643	--	-0.45	-0.45
368	1642	--	-0.51	-0.51

369	1641	--		-0.41	-0.41
370	1640	--		-0.83	-0.83
371	1639	--		0.62	0.62
372	1638	--		-0.59	-0.59
373	1637	--		-0.71	-0.71
374	1636	--		-0.79	-0.79
375	1635	--		-0.9	-0.9
376	1634	--		0.09	0.09
377	1633	--		-0.4	-0.4
378	1632	--		-0.55	-0.55
379	1631	--		-0.28	-0.28
380	1630	--		0.27	0.27
381	1629	--		-0.16	-0.16
382	1628	--		0.11	0.11
383	1627	--		-0.1	-0.1
384	1626	--		0.07	0.07
385	1625	--		-0.4	-0.4
386	1624	--		0.23	0.23
387	1623	--		-0.02	-0.02
388	1622	--		-0.36	-0.36
389	1621	--		0.66	0.66
390	1620	--		-0.12	-0.12
391	1619	--		1.68	1.68
392	1618	--		0.02	0.02
393	1617	--		0.97	0.97
394	1616	--		0.35	0.35
395	1615	--		2.99	2.99
396	1614		4.35	1.42	2.89
397	1613		0.76	1.91	1.34
398	1612		0.87	1.66	1.27
399	1611		2.99	1.76	2.37
400	1610		2.77	0.99	1.88
401	1609		3.15	3.28	3.22
402	1608		2.12	1.68	1.9

403	1607	2.66	2.68	2.67
404	1606	1.36	1.6	1.48
405	1605	1.31	1.76	1.53
406	1604	2.23	2.38	2.31
407	1603	0.65	2.3	1.48
408	1602	1.9	1.76	1.83
409	1601	0.22	1.84	1.03
410	1600	0.87	1.03	0.95
411	1599	-0.11	0.88	0.39
412	1598	0.27	0.33	0.3
413	1597	-0.11	1.03	0.46
414	1596	-0.16	0.86	0.35
415	1595	-0.16	1.99	0.92
416	1594	-0.05	0.66	0.31
417	1593	0.06	-0.45	-0.2
418	1592	0.38	0.7	0.54
419	1591	0.71	2.13	1.42
420	1590	0.38	2.36	1.37
421	1589	0.33	4.5	2.41
422	1588	1.09	4.14	2.62
423	1587	0.33	1.68	1
424	1586	0.55	1.6	1.07
425	1585	0.22	1.37	0.79
426	1584	0.49	1.7	1.09
427	1583	-0.21	2.85	1.32
428	1582	-0.11	4.26	2.08
429	1581	-0.27	4.69	2.21
430	1580	-0.49	2.62	1.07
431	1579	-0.38	3.15	1.38
432	1578	-0.49	4.34	1.93
433	1577	-0.32	2.97	1.32
434	1576	-0.16	1.33	0.58
435	1575	-0.16	0.94	0.39
436	1574	-0.38	0.88	0.25

437	1573	-0.32	0.78	0.23
438	1572	-0.54	0.49	-0.03
439	1571	-0.05	0.49	0.22
440	1570	-0.16	1.52	0.68
441	1569	-0.43	0.11	-0.16
442	1568	0.06	-0.2	-0.07
443	1567	0.22	0.58	0.4
444	1566	-0.05	2.38	1.17
445	1565	0.06	1.15	0.6
446	1564	0	1.56	0.78
447	1563	0.11	2.99	1.55
448	1562	0.44	0.97	0.71
449	1561	1.03	3.28	2.16
450	1560	0.71	2.6	1.65
451	1559	1.03	1.62	1.33
452	1558	0.11	1.84	0.97
453	1557	0.22	2.11	1.16
454	1556	0.06	0.9	0.48
455	1555	0.27	1.29	0.78
456	1554	0.22	1.13	0.68
457	1553	-0.32	1.48	0.58
458	1552	-0.16	1.05	0.45
459	1551	-0.65	2.05	0.7
460	1550	-0.76	2.05	0.65
461	1549	-0.7	2.83	1.07
462	1548	-0.65	1.87	0.61
463	1547	-0.65	2.03	0.69
464	1546	-0.21	1.91	0.85
465	1545	-0.65	2.21	0.78
466	1544	-0.54	3.5	1.48
467	1543	-0.32	5.83	2.75
468	1542	-0.65	3.05	1.2
469	1541	-0.7	1.4	0.35
470	1540	-0.76	3.4	1.32

471	1539	-0.59	2.74	1.07
472	1538	-0.21	3.03	1.41
473	1537	-0.11	2.25	1.07
474	1536	-0.54	1.42	0.44
475	1535	0.17	1.95	1.06
476	1534	0.6	2.46	1.53
477	1533	0.27	1.44	0.86
478	1532	-0.27	2.34	1.04
479	1531	-0.43	1.6	0.58
480	1530	0.17	2.38	1.27
481	1529	0.17	1.68	0.92
482	1528	0.11	1.29	0.7
483	1527	0.38	1.8	1.09
484	1526	0.33	1.52	0.93
485	1525	0.06	1.95	1
486	1524	-0.32	2.93	1.3
487	1523	-0.43	1.87	0.72
488	1522	-0.05	1.91	0.93
489	1521	-0.32	2.23	0.95
490	1520	-0.27	1.95	0.84
491	1519	-0.43	2.09	0.83
492	1518	-0.7	0.54	-0.08
493	1517	0.33	0.9	0.61
494	1516	-0.11	1.29	0.59
495	1515	-0.27	1.13	0.43
496	1514	-0.16	1.95	0.9
497	1513	-0.11	1.29	0.59
498	1512	-0.32	1.29	0.48
499	1511	-0.54	2.23	0.84
500	1510	-0.27	2.38	1.06
501	1509	-0.32	2.62	1.15
502	1508	-0.38	1.78	0.7
503	1507	-0.16	3.22	1.53
504	1506	-0.54	2.38	0.92

505	1505	0.27	3.28	1.78
506	1504	-0.49	3.01	1.26
507	1503	0.17	2.97	1.57
508	1502	-0.49	3.77	1.64
509	1501	-0.43	3.28	1.43
510	1500	-0.65	2.89	1.12
511	1499	0.11	3.01	1.56
512	1498	-0.43	4.18	1.88
513	1497	-0.27	3.07	1.4
514	1496	-0.32	1.7	0.69
515	1495	-0.38	1.97	0.8
516	1494	-0.49	2.15	0.83
517	1493	-0.32	2.23	0.95
518	1492	-0.54	1.23	0.34
519	1491	-0.49	1.25	0.38
520	1490	-0.49	1.64	0.58
521	1489	-0.38	1.91	0.77
522	1488	-0.32	1.56	0.62
523	1487	0.06	0.9	0.48
524	1486	0.11	1.7	0.9
525	1485	-0.32	0.95	0.32
526	1484	0.22	1.64	0.93
527	1483	0	1.17	0.59
528	1482	0.27	0.78	0.53
529	1481	0.06	1.46	0.76
530	1480	0.38	2.48	1.43
531	1479	0	2.38	1.19
532	1478	-0.16	1.99	0.92
533	1477	-0.32	2.89	1.28
534	1476	-0.49	1.82	0.66
535	1475	-0.32	1.48	0.58
536	1474	-0.43	0.94	0.25
537	1473	-0.43	1.29	0.43
538	1472	-0.16	0.66	0.25

539	1471	-0.76	0.47	-0.15
540	1470	-0.27	1.25	0.49
541	1469	-0.27	0.94	0.33
542	1468	-0.43	1.56	0.56
543	1467	1.25	0.62	0.94
544	1466	0.65	0.99	0.82
545	1465	1.52	0.78	1.15
546	1464	-0.76	0.62	-0.07
547	1463	1.09	1.6	1.34
548	1462	0.22	1.05	0.64
549	1461	0.44	1.01	0.73
550	1460	2.12	0.07	1.1
551	1459	0.98	0.11	0.55
552	1458	0.6	-0.36	0.12
553	1457	0.6	-0.02	0.29
554	1456	0.93	-0.28	0.32
555	1455	0.44	-0.16	0.14
556	1454	0.49	1.05	0.77
557	1453	1.74	0.19	0.97
558	1452	3.53	0.15	1.84
559	1451	1.58	0.82	1.2
560	1450	1.41	0.15	0.78
561	1449	-0.21	0.07	-0.07
562	1448	1.03	1.13	1.08
563	1447	1.03	0.23	0.63
564	1446	0.06	0.86	0.46
565	1445	0.27	0.23	0.25
566	1444	0.49	--	0.49
567	1443	0.55	--	0.55
568	1442	0.33	--	0.33
569	1441	0.11	--	0.11
570	1440	0.27	--	0.27
571	1439	-0.05	--	-0.05
572	1438	0.82	--	0.82

573	1437	-0.43	--	-0.43
574	1436	0.17	--	0.17
575	1435	1.03	--	1.03
576	1434	0.22	--	0.22
577	1433	-0.65	--	-0.65
578	1432	-0.16	--	-0.16
579	1431	-0.43	--	-0.43
580	1430	-0.05	--	-0.05
581	1429	-0.54	--	-0.54
582	1428	0.44	--	0.44
583	1427	0.38	--	0.38
584	1426	0	--	0
585	1425	-0.16	--	-0.16
586	1424	-0.59	--	-0.59
587	1423	-0.59	0.07	-0.26
588	1422	-0.97	0.43	-0.27
589	1421	-0.32	-0.41	-0.37
590	1420	-0.59	-0.12	-0.36
591	1419	-0.32	-0.51	-0.42
592	1418	-0.27	-0.98	-0.63
593	1417	-0.38	-0.63	-0.5
594	1416	-0.32	0.04	-0.14
595	1415	-0.49	-0.67	-0.58
596	1414	-0.32	-0.43	-0.38
597	1413	-0.05	-1.06	-0.56
598	1412	-0.43	-0.71	-0.57
599	1411	0	-0.12	-0.06
600	1410	-0.11	-0.63	-0.37
601	1409	-0.65	-0.51	-0.58
602	1408	-0.7	-0.83	-0.76
603	1407	-0.7	-0.9	-0.8
604	1406	-0.49	-0.69	-0.59
605	1405	0.06	-0.57	-0.26
606	1404	0	-0.61	-0.3

607	1403	-0.38	-0.61	-0.49
608	1402	-0.32	-0.32	-0.32
609	1401	-0.27	-0.51	-0.39
610	1400	-0.43	-0.12	-0.28
611	1399	-0.38	-0.12	-0.25
612	1398	-0.81	-0.43	-0.62
613	1397	0.27	-0.67	-0.2
614	1396	0.49	-0.63	-0.07
615	1395	-0.27	-0.59	-0.43
616	1394	-0.27	-0.47	-0.37
617	1393	-0.54	-0.53	-0.54
618	1392	-0.7	-1.04	-0.87
619	1391	-0.43	-0.73	-0.58
620	1390	-0.38	-1.14	-0.76
621	1389	-0.32	-0.94	-0.63
622	1388	-0.16	-0.83	-0.49
623	1387	-0.87	-0.94	-0.9
624	1386	-0.7	-1.02	-0.86
625	1385	-0.49	-0.75	-0.62
626	1384	-0.11	-0.59	-0.35
627	1383	0.06	-0.83	-0.38
628	1382	-0.32	-0.94	-0.63
629	1381	-0.38	-1.02	-0.7
630	1380	-0.05	-0.75	-0.4
631	1379	-0.05	-0.98	-0.52
632	1378	0.06	-1.14	-0.54
633	1377	0.27	-0.94	-0.33
634	1376	0.11	-1.33	-0.61
635	1375	-0.21	-1.02	-0.62
636	1374	-0.38	-1.33	-0.86
637	1373	-0.43	-1.37	-0.9
638	1372	-0.32	-1.14	-0.73
639	1371	-0.54	-1.26	-0.9
640	1370	-0.05	-1.06	-0.56

641	1369	-0.16	-0.75	-0.45
642	1368	0	-0.79	-0.39
643	1367	0.06	-0.79	-0.36
644	1366	-0.16	-0.83	-0.49
645	1365	0.22	-0.98	-0.38
646	1364	0.22	-0.83	-0.3
647	1363	0.27	-0.75	-0.24
648	1362	-0.49	-0.86	-0.68
649	1361	-0.49	-0.43	-0.46
650	1360	-0.11	-1.02	-0.56
651	1359	0.55	-0.86	-0.16
652	1358	0.49	-0.75	-0.13
653	1357	0.65	-0.98	-0.16
654	1356	-0.11	-1.1	-0.6
655	1355	-0.21	-1.18	-0.7
656	1354	-0.59	-1.06	-0.83
657	1353	0.27	-1.22	-0.47
658	1352	0.27	-0.79	-0.26
659	1351	0.11	-1.08	-0.48
660	1350	-0.11	-1.14	-0.62
661	1349	-1.03	-1.14	-1.08
662	1348	-1.08	-0.9	-0.99
663	1347	0.11	-1.1	-0.49
664	1346	0.17	-0.98	-0.41
665	1345	0	-0.86	-0.43
666	1344	-0.32	-0.71	-0.52
667	1343	0.11	-0.9	-0.4
668	1342	0.93	-0.9	0.01
669	1341	0.71	-1.14	-0.21
670	1340	-0.65	-0.32	-0.48
671	1339	0.27	-0.86	-0.3
672	1338	-0.49	-0.83	-0.66
673	1337	-0.54	-0.63	-0.59
674	1336	-0.27	-0.71	-0.49

675	1335	-0.76	-1.02	-0.89
676	1334	-0.97	-1.02	-1
677	1333	-0.38	-1.02	-0.7
678	1332	-0.43	-0.9	-0.67
679	1331	-0.59	-1.02	-0.81
680	1330	-0.76	-1.18	-0.97
681	1329	-1.08	-1.12	-1.1
682	1328	-1.08	-0.98	-1.03
683	1327	-0.97	-1.3	-1.13
684	1326	-1.14	-1.08	-1.11
685	1325	-1.03	-1.26	-1.14
686	1324	-0.7	-1.14	-0.92
687	1323	-0.97	-1.08	-1.03
688	1322	-0.87	-0.98	-0.92
689	1321	-0.38	-1.2	-0.79
690	1320	-0.76	-1.18	-0.97
691	1319	-0.87	-1.26	-1.06
692	1318	-0.97	-1.2	-1.09
693	1317	-0.97	-1.33	-1.15
694	1316	-1.08	-1.16	-1.12
695	1315	-1.03	-1.49	-1.26
696	1314	-1.14	-1.16	-1.15
697	1313	-0.92	-1.26	-1.09
698	1312	-1.03	-1.22	-1.12
699	1311	-0.97	-1.37	-1.17
700	1310	-1.25	-1.33	-1.29
701	1309	-1.08	-1.26	-1.17
702	1308	-1.19	-0.86	-1.03
703	1307	-1.14	-1.18	-1.16
704	1306	-1.03	-1.1	-1.06
705	1305	-1.03	-1.1	-1.06
706	1304	-1.19	-0.94	-1.07
707	1303	-0.76	-1.45	-1.1
708	1302	-1.03	-1.37	-1.2

709	1301	-0.81	-1.3	-1.05
710	1300	-0.81	-1.53	-1.17
711	1299	-0.87	-1.37	-1.12
712	1298	-0.87	-1.14	-1
713	1297	-0.81	-1.18	-0.99
714	1296	-0.81	-1.3	-1.05
715	1295	-0.87	-1.26	-1.06
716	1294	-0.97	-1.26	-1.12
717	1293	-0.7	-1.1	-0.9
718	1292	-0.65	-1.33	-0.99
719	1291	-0.92	-1.57	-1.24
720	1290	-0.81	-1.45	-1.13
721	1289	-0.97	-1.02	-1
722	1288	-1.25	-1.41	-1.33
723	1287	-1.14	-1.31	-1.23
724	1286	-0.7	-1.37	-1.04
725	1285	-0.92	-1.57	-1.24
726	1284	-1.14	-1.45	-1.29
727	1283	-1.19	-1.18	-1.18
728	1282	-0.97	-1.14	-1.06
729	1281	-0.97	-1.37	-1.17
730	1280	-1.14	-1.45	-1.29
731	1279	-0.7	-1.3	-1
732	1278	-0.81	-1.49	-1.15
733	1277	-0.97	-1.49	-1.23
734	1276	-0.81	-1.49	-1.15
735	1275	-0.92	-1.33	-1.13
736	1274	-1.08	-1.45	-1.27
737	1273	-1.19	-1.61	-1.4
738	1272	-1.3	-1.53	-1.42
739	1271	-1.08	-1.61	-1.35
740	1270	-1.14	-1.37	-1.26
741	1269	-1.14	-1.61	-1.37
742	1268	-0.97	-1.49	-1.23

743	1267	-1.03	-1.49	-1.26
744	1266	-1.08	-1.63	-1.36
745	1265	-0.97	-1.69	-1.33
746	1264	-0.81	-1.57	-1.19
747	1263	-0.97	-1.49	-1.23
748	1262	-0.97	-1.65	-1.31
749	1261	-1.08	-1.49	-1.29
750	1260	-1.19	-1.57	-1.38
751	1259	-0.97	-1.49	-1.23
752	1258	-1.14	-1.18	-1.16
753	1257	-1.03	-1.33	-1.18
754	1256	-0.76	-1.54	-1.15
755	1255	-0.92	-1.54	-1.23
756	1254	-0.87	-1.46	-1.16
757	1253	-0.92	-1.51	-1.22
758	1252	-1.14	-1.48	-1.31
759	1251	-1.14	-1.56	-1.35
760	1250	-1.08	-1.58	-1.33
761	1249	-0.92	-1.57	-1.24
762	1248	-1.19	-1.46	-1.33
763	1247	-1.19	-1.57	-1.38
764	1246	-1.14	-1.6	-1.37
765	1245	-1.25	-1.71	-1.48
766	1244	-1.25	-1.71	-1.48
767	1243	-0.87	-1.75	-1.31
768	1242	-0.7	-1.57	-1.14
769	1241	-0.38	-1.61	-0.99
770	1240	-0.65	-1.53	-1.09
771	1239	-0.54	-1.61	-1.07
772	1238	-0.76	-1.35	-1.06
773	1237	-0.7	-1.63	-1.17
774	1236	-0.38	-1.65	-1.01
775	1235	-0.38	-1.67	-1.02
776	1234	-0.43	-1.51	-0.97

777	1233	-0.16	-1.55	-0.85
778	1232	-0.32	-1.39	-0.86
779	1231	-0.27	-1.45	-0.86
780	1230	-0.21	-1.22	-0.72
781	1229	-0.27	-0.8	-0.53
782	1228	-0.49	-1.21	-0.85
783	1227	-0.81	-1.31	-1.06
784	1226	-0.76	-1.42	-1.09
785	1225	-0.49	-1.19	-0.84
786	1224	-0.7	-1.31	-1
787	1223	-0.76	-1.24	-1
788	1222	-0.59	-1.28	-0.93
789	1221	-1.08	-1.36	-1.22
790	1220	-0.87	-1.31	-1.09
791	1219	-0.92	-1.25	-1.08
792	1218	-1.14	-1.27	-1.2
793	1217	-0.7	-1.38	-1.04
794	1216	-0.81	-1.37	-1.09
795	1215	-0.7	-1.41	-1.06
796	1214	-0.7	-1.45	-1.08
797	1213	-0.97	-1.65	-1.31
798	1212	-1.14	-1.57	-1.35
799	1211	-0.76	-1.53	-1.14
800	1210	-0.27	-1.61	-0.94
801	1209	-0.65	-1.57	-1.11
802	1208	-0.76	-1.33	-1.05
803	1207	-0.76	-1.26	-1.01
804	1206	-0.81	-1.45	-1.13
805	1205	-0.49	-1.57	-1.03
806	1204	-0.7	-1.61	-1.16
807	1203	-0.49	-1.69	-1.09
808	1202	-0.43	-1.57	-1
809	1201	-0.32	-1.57	-0.95
810	1200	-0.65	-1.65	-1.15

811	1199	-0.65	-1.65	-1.15
812	1198	-0.7	-1.57	-1.14
813	1197	-0.76	-1.49	-1.12
814	1196	-0.7	-1.45	-1.08
815	1195	-1.03	-1.35	-1.19
816	1194	-0.81	-1.41	-1.11
817	1193	-0.87	-1.61	-1.24
818	1192	-0.97	-1.57	-1.27
819	1191	-0.97	-1.41	-1.19
820	1190	-0.81	-1.49	-1.15
821	1189	-0.7	-1.61	-1.16
822	1188	-0.76	-1.73	-1.24
823	1187	-0.81	-1.45	-1.13
824	1186	-1.03	-1.26	-1.14
825	1185	-1.14	-1.45	-1.29
826	1184	-0.87	-1.3	-1.08
827	1183	-0.59	-1.41	-1
828	1182	-0.87	-1.41	-1.14
829	1181	-0.7	-1.18	-0.94
830	1180	-1.03	-1.41	-1.22
831	1179	-1.14	-1.37	-1.26
832	1178	-0.87	-1.26	-1.06
833	1177	-0.87	-1.26	-1.06
834	1176	-0.81	-1.26	-1.03
835	1175	-0.92	-1.37	-1.15
836	1174	-0.81	-1.57	-1.19
837	1173	-1.03	-1.3	-1.16
838	1172	-1.08	-1.12	-1.1
839	1171	-0.65	-1.45	-1.05
840	1170	-0.54	-1.14	-0.84
841	1169	-0.81	-1.14	-0.98
842	1168	-0.81	-1.26	-1.03
843	1167	-0.81	-1.14	-0.98
844	1166	-0.97	-1.18	-1.08

845	1165	-0.87	-1.08	-0.97
846	1164	-0.76	-1.1	-0.93
847	1163	-0.7	-1.3	-1
848	1162	-0.65	-0.9	-0.78
849	1161	-0.87	-0.86	-0.87
850	1160	-1.14	-0.94	-1.04
851	1159	-0.92	-1.1	-1.01
852	1158	-0.81	-0.9	-0.86
853	1157	-0.7	-0.3	-0.5
854	1156	-0.81	-1.14	-0.98
855	1155	-0.81	-1.02	-0.92
856	1154	-0.81	-0.69	-0.75
857	1153	-0.7	-0.75	-0.73
858	1152	-0.92	-0.86	-0.89
859	1151	-0.76	-0.98	-0.87
860	1150	-0.81	-0.98	-0.9
861	1149	-0.65	-0.9	-0.78
862	1148	-0.97	-1.18	-1.08
863	1147	-0.59	-1.14	-0.87
864	1146	-0.54	-1.1	-0.82
865	1145	-0.97	-1.1	-1.04
866	1144	-0.49	-1.14	-0.81
867	1143	-0.81	-0.98	-0.9
868	1142	-0.97	-1.22	-1.1
869	1141	-1.08	-1.33	-1.21
870	1140	-0.76	-1.06	-0.91
871	1139	-0.87	-0.86	-0.87
872	1138	-1.14	-0.51	-0.82
873	1137	-0.81	-0.92	-0.87
874	1136	-0.81	-0.83	-0.82
875	1135	-0.92	-1.06	-0.99
876	1134	-0.81	-0.75	-0.78
877	1133	-0.7	-0.47	-0.59
878	1132	-0.92	-0.55	-0.74

879	1131	-0.87	-0.75	-0.81
880	1130	-1.03	-0.4	-0.71
881	1129	-0.97	-0.2	-0.59
882	1128	-0.76	-0.51	-0.63
883	1127	-0.7	-0.67	-0.69
884	1126	-0.54	-0.75	-0.64
885	1125	-0.65	-0.86	-0.76
886	1124	-0.7	-0.9	-0.8
887	1123	-1.03	-0.51	-0.77
888	1122	-0.59	-0.63	-0.61
889	1121	-0.38	-0.16	-0.27
890	1120	-0.49	-0.32	-0.4
891	1119	-0.81	-0.57	-0.69
892	1118	-0.76	-0.67	-0.71
893	1117	-1.08	-0.57	-0.83
894	1116	-1.19	-0.63	-0.91
895	1115	-0.54	-0.79	-0.66
896	1114	-0.59	-0.79	-0.69
897	1113	-0.54	-0.63	-0.59
898	1112	-0.38	-1.02	-0.7
899	1111	-0.49	-0.9	-0.69
900	1110	0.11	-0.43	-0.16
901	1109	-0.32	-0.43	-0.38
902	1108	-0.32	-0.16	-0.24
903	1107	-0.7	0.15	-0.28
904	1106	-0.7	-0.04	-0.37
905	1105	-0.92	-0.24	-0.58
906	1104	-1.03	-0.53	-0.78
907	1103	-0.7	-0.53	-0.62
908	1102	-0.54	-0.77	-0.65
909	1101	-0.87	-0.63	-0.75
910	1100	-0.27	-0.32	-0.29
911	1099	-0.49	-0.71	-0.6
912	1098	-0.38	-0.63	-0.5

913	1097	-0.7	-0.71	-0.71
914	1096	-0.38	-0.79	-0.58
915	1095	-0.11	-0.86	-0.49
916	1094	-0.59	-0.86	-0.73
917	1093	-0.87	-0.51	-0.69
918	1092	-0.49	-0.86	-0.68
919	1091	-1.19	-0.55	-0.87
920	1090	-1.14	-0.59	-0.86
921	1089	-1.08	-0.24	-0.66
922	1088	-0.76	-0.32	-0.54
923	1087	-0.7	-0.4	-0.55
924	1086	-0.76	-0.24	-0.5
925	1085	-0.43	-0.32	-0.37
926	1084	-0.43	-0.28	-0.35
927	1083	-0.43	-0.71	-0.57
928	1082	-0.49	0.35	-0.07
929	1081	-0.38	-0.16	-0.27
930	1080	0.49	0.05	0.27
931	1079	-0.59	-0.28	-0.44
932	1078	0	0.23	0.12
933	1077	0.33	-0.24	0.05
934	1076	1.63	-0.04	0.79
935	1075	0.98	-0.41	0.28
936	1074	1.58	-0.45	0.56
937	1073	0.06	-0.83	-0.38
938	1072	0.11	-0.73	-0.31
939	1071	0	-0.61	-0.3
940	1070	0.17	-0.85	-0.34
941	1069	1.03	-0.9	0.07
942	1068	-0.21	-0.32	-0.27
943	1067	0.44	-0.47	-0.02
944	1066	0.22	-0.69	-0.23
945	1065	0.22	-1	-0.39
946	1064	0	-0.9	-0.45

947	1063	0.22	-0.53	-0.16
948	1062	0.22	-0.63	-0.2
949	1061	-0.38	-0.88	-0.63
950	1060	-0.21	-0.55	-0.38
951	1059	0.49	-0.4	0.05
952	1058	0.49	-0.47	0.01
953	1057	0.49	-0.47	0.01
954	1056	0.22	-0.67	-0.22
955	1055	0.33	-0.59	-0.13
956	1054	0.49	-0.55	-0.03
957	1053	0.38	0.19	0.29
958	1052	0.22	0.07	0.15
959	1051	0.49	0.27	0.38
960	1050	-0.16	-0.32	-0.24
961	1049	0.11	0.35	0.23
962	1048	0.06	0.15	0.1
963	1047	0.22	-0.32	-0.05
964	1046	0.33	0.39	0.36
965	1045	0.49	0.43	0.46
966	1044	0.22	-0.16	0.03
967	1043	-0.49	-0.59	-0.54
968	1042	0.93	-0.24	0.34
969	1041	0.38	-0.04	0.17
970	1040	0	0.11	0.06
971	1039	0.33	0.43	0.38
972	1038	-0.11	0	-0.05
973	1037	0.27	0	0.14
974	1036	-0.27	-0.55	-0.41
975	1035	1.31	-0.24	0.53
976	1034	-0.32	0.54	0.11
977	1033	0	-0.38	-0.19
978	1032	-0.38	0.23	-0.07
979	1031	-0.27	0.19	-0.04
980	1030	-1.14	-0.38	-0.76

981	1029	-1.03	-0.4	-0.71
982	1028	-1.14	-0.3	-0.72
983	1027	-1.08	-0.12	-0.6
984	1026	-1.08	-0.38	-0.73
985	1025	-0.92	-0.38	-0.65
986	1024	-0.87	-0.32	-0.59
987	1023	-1.14	-0.75	-0.94
988	1022	-1.03	-0.86	-0.95
989	1021	-1.14	-0.55	-0.84
990	1020	-1.19	-0.98	-1.09
991	1019	-1.08	-0.51	-0.8
992	1018	-0.49	-0.83	-0.66
993	1017	-0.76	-0.2	-0.48
994	1016	-1.25	-0.59	-0.92
995	1015	-1.08	-0.79	-0.93
996	1014	-0.97	-0.71	-0.84
997	1013	-1.03	-0.47	-0.75
998	1012	-1.03	-0.79	-0.91
999	1011	-1.08	-0.57	-0.83
1000	1010	-1.14	-0.51	-0.82
1001	1009	-1.19	-0.55	-0.87
1002	1008	-0.54	-0.49	-0.52
1003	1007	-0.76	-0.59	-0.67
1004	1006	-1.03	-0.69	-0.86
1005	1005	-1.03	-0.77	-0.9
1006	1004	-0.92	-0.41	-0.67
1007	1003	-1.14	-0.96	-1.05
1008	1002	-1.03	-0.59	-0.81
1009	1001	-1.08	-0.55	-0.82
1010	1000	-1.14	-0.63	-0.88
1011	999	-0.97	-0.12	-0.55
1012	998	-1.14	-0.04	-0.59
1013	997	-1.14	0.07	-0.53
1014	996	-1.19	-0.55	-0.87

1015	995	-1.08	-0.36	-0.72
1016	994	-0.97	-0.71	-0.84
1017	993	-0.43	-0.36	-0.39
1018	992	-0.97	-0.08	-0.53
1019	991	-0.49	-0.16	-0.32
1020	990	-1.08	0.27	-0.41
1021	989	-1.19	-0.47	-0.83
1022	988	-1.19	0.45	-0.37
1023	987	-1.19	0.23	-0.48
1024	986	-1.14	0.04	-0.55
1025	985	-1.19	0.43	-0.38
1026	984	-0.49	0.82	0.17
1027	983	-1.14	0.97	-0.08
1028	982	-1.03	0.31	-0.36
1029	981	-0.76	0.47	-0.15
1030	980	-1.08	0.23	-0.43
1031	979	-0.87	0.19	-0.34
1032	978	-1.14	-0.26	-0.7
1033	977	-0.65	0.11	-0.27
1034	976	-0.38	0.02	-0.18
1035	975	-0.49	-0.3	-0.39
1036	974	-0.76	0.23	-0.26
1037	973	-0.59	0.17	-0.21
1038	972	-0.76	0.9	0.07
1039	971	-0.49	0.66	0.09
1040	970	-0.27	0.35	0.04
1041	969	-0.49	0.15	-0.17
1042	968	-0.54	0.31	-0.12
1043	967	-0.81	-0.12	-0.47
1044	966	-1.14	-0.43	-0.79
1045	965	-0.76	-0.43	-0.6
1046	964	-0.97	-0.18	-0.58
1047	963	-0.97	-0.2	-0.59
1048	962	-1.19	0.84	-0.18

1049	961	-1.3	0.15	-0.57
1050	960	-0.81	0.19	-0.31
1051	959	-0.65	-0.47	-0.56
1052	958	-0.49	-0.36	-0.42
1053	957	-0.54	-0.59	-0.57
1054	956	-0.7	-0.51	-0.61
1055	955	-0.76	-0.4	-0.58
1056	954	-0.7	-0.83	-0.76
1057	953	-1.25	-0.55	-0.9
1058	952	-1.25	-0.08	-0.66
1059	951	-1.08	-0.08	-0.58
1060	950	-1.14	0.27	-0.43
1061	949	-1.3	-0.16	-0.73
1062	948	-1.14	-0.2	-0.67
1063	947	-1.14	0.11	-0.51
1064	946	-1.14	0.07	-0.53
1065	945	-0.81	-0.1	-0.46
1066	944	-0.87	-0.14	-0.5
1067	943	-0.65	-0.04	-0.35
1068	942	-1.03	-0.18	-0.6
1069	941	-1.3	-0.38	-0.84
1070	940	-1.25	-0.1	-0.67
1071	939	-1.3	0.19	-0.55
1072	938	-0.97	0.23	-0.37
1073	937	-1.19	-0.04	-0.62
1074	936	-1.14	-0.28	-0.71
1075	935	-0.97	-0.08	-0.53
1076	934	-0.76	-0.28	-0.52
1077	933	-0.81	0.07	-0.37
1078	932	-0.7	-0.14	-0.42
1079	931	-1.03	-0.12	-0.57
1080	930	-1.25	0.04	-0.61
1081	929	-0.59	-0.51	-0.55
1082	928	-0.43	-0.43	-0.43

1083	927	-0.7			-0.32	-0.51
1084	926	-0.65			0.39	-0.13
1085	925	-0.76			0.86	0.05
1086	924	-0.81			0.23	-0.29
1087	923	-1.03			-0.12	-0.57
1088	922	-0.59			-0.12	-0.36
1089	921	-0.32			0.5	0.09
1090	920	-0.76			0.27	-0.24
1091	919	-0.97			0	-0.49
1092	918	-0.92	--	--	-0.04	-0.48
1093	917	-1.35	--	--	0.07	-0.64
1094	916	-1.14	--	--	0.39	-0.37
1095	915	-1.25	--	--	0	-0.62
1096	914	-1.08	--	--	0.04	-0.52
1097	913	-1.03	--	--	0.23	-0.4
1098	912	-0.16	--	--	0.78	0.31
1099	911	0.33	--	--	0.62	0.48
1100	910	0.33	--	--	-0.04	0.14
1101	909	0.06	--	--	0.07	0.07
1102	908	-0.81	--	--	1.64	0.41
1103	907	-0.43	--	--	0.19	-0.12
1104	906	-0.54	--	--	0.35	-0.1
1105	905		0.06	0.01	0.72	0.26
1106	904		0.11	0.68	0.02	0.27
1107	903		-0.62	-0.2	0.33	-0.16
1108	902		-0.4	0.74	0.25	0.2
1109	901		-0.7	1.34	0.7	0.45
1110	900		-0.98	0.56	0.21	-0.07
1111	899		-0.57	0.3	1.37	0.37
1112	898		-0.35	0.85	0.27	0.26
1113	897		-0.79	0.62	-0.47	-0.21
1114	896		-0.7	0.22	-0.28	-0.26
1115	895		-0.6	1.29	1.23	0.64
1116	894		-0.67	1.08	0.84	0.41

1117	893	-0.62	0.88	0.62	0.29
1118	892	-0.74	0.85	0.78	0.29
1119	891	-0.98	0.56	1.17	0.25
1120	890	-0.6	0.73	0.58	0.24
1121	889	-0.84	0.94	0.97	0.36
1122	888	-0.51	0.94	0.74	0.39
1123	887	-0.4	0.73	0.23	0.19
1124	886	-0.62	1.43	0.95	0.59
1125	885	-0.52	1.37	0.86	0.57
1126	884	-0.78	1.69	0.19	0.37
1127	883	-0.68	0.36	0.04	-0.1
1128	882	-0.91	0.47	0.97	0.18
1129	881	-0.74	1.78	1.48	0.84
1130	880	-0.51	1.43	0.74	0.55
1131	879	-0.85	1.37	0.15	0.23
1132	878	-0.84	1.55	-0.12	0.2
1133	877	-0.97	1.31	0.86	0.4
1134	876	-0.86	0.99	0.31	0.15
1135	875	-0.84	0.33	0.27	-0.08
1136	874	-0.87	1.29	0.43	0.28
1137	873	-0.81	0.88	0.58	0.22
1138	872	-0.81	1.63	1.17	0.66
1139	871	-1.01	1.05	0.35	0.13
1140	870	-0.5	0.77	1.52	0.59
1141	869	-0.63	1.58	1.48	0.81
1142	868	-0.78	1.26	0.5	0.33
1143	867	-0.86	0.99	0.8	0.31
1144	866	-0.83	0.79	0.5	0.16
1145	865	-0.6	0.71	-0.18	-0.03
1146	864	-0.56	0.62	-0.2	-0.05
1147	863	-0.88	1.41	0.04	0.19
1148	862	-1.07	1.37	-0.32	-0.01
1149	861	-0.68	0.44	-0.24	-0.16
1150	860	-0.51	1.49	-0.41	0.19

1151	859	-0.6	0.88	-0.65	-0.12
1152	858	-0.57	0.77	-0.55	-0.12
1153	857	-0.38	0.97	-0.53	0.02
1154	856	-0.24	1.26	-0.53	0.16
1155	855	-0.32	1	-0.2	0.16
1156	854	-0.85	1.58	-0.28	0.15
1157	853	-0.5	0.73	-0.18	0.02
1158	852	-0.66	1.7	0.07	0.37
1159	851	-0.76	1.58	0.7	0.5
1160	850	-0.93	1.63	0.43	0.38
1161	849	-0.45	1.26	0.47	0.42
1162	848	-0.59	1.58	0.5	0.5
1163	847	-0.46	1.95	0.58	0.69
1164	846	-0.9	1	0.05	0.05
1165	845	-0.83	1.14	0.43	0.24
1166	844	-0.26	1.61	0.5	0.62
1167	843	-0.41	1.6	-0.04	0.38
1168	842	-0.86	1.49	-0.36	0.09
1169	841	-0.75	1.2	0.54	0.33
1170	840	-0.81	0.82	0.54	0.19
1171	839	-0.79	0.48	0.11	-0.07
1172	838	-1.17	0.79	-0.2	-0.19
1173	837	-1.18	0.33	0.11	-0.25
1174	836	-1.23	0.71	0.5	-0.01
1175	835	-1.05	0.85	0.94	0.24
1176	834	-1.21	1	0.74	0.18
1177	833	-1.11	0.73	0.78	0.13
1178	832	-0.91	0.1	1.01	0.07
1179	831	-1.02	0.56	0.47	0
1180	830	-1.02	0.48	0.19	-0.12
1181	829	-1.14	0.99	0.5	0.12
1182	828	-1	0.33	0.54	-0.04
1183	827	-1.04	0.19	0.6	-0.08
1184	826	-0.81	0.91	0.5	0.2

1185	825	-1.06	0.97	0.04	-0.02
1186	824	-0.93	0.91	0.86	0.28
1187	823	-1.28	0.53	1.09	0.11
1188	822	-1.26	0.8	1.4	0.31
1189	821	-1.08	0.39	1.37	0.23
1190	820	-1.01	1.29	1.84	0.71
1191	819	-1.09	0.97	0.86	0.25
1192	818	-1.01	0.5	1.68	0.39
1193	817	-1.04	1.11	2.36	0.81
1194	816	-1.12	0.68	1.64	0.4
1195	815	-1.2	0.04	2.34	0.4
1196	814	-1.24	1.26	1.64	0.55
1197	813	-1.02	0.91	1.21	0.37
1198	812	-1.08	1.23	0.58	0.25
1199	811	-1.02	1.41	0.31	0.23
1200	810	-0.89	1.4	0.74	0.42
1201	809	-0.81	1.14	1.37	0.57
1202	808	-0.59	1.86	0.76	0.68
1203	807	-0.92	0.71	1.11	0.3
1204	806	-0.73	1.63	1.48	0.79
1205	805	-0.92	1.02	1.64	0.58
1206	804	-1.05	0.85	1.4	0.4
1207	803	-0.89	0.94	1.33	0.46
1208	802	-0.73	1.26	1.29	0.6
1209	801	-0.88	1.2	1.62	0.65
1210	800	-0.85	1.11	0.9	0.39
1211	799	-0.56	0.94	0.9	0.42
1212	798	-0.58	1.2	0.7	0.44
1213	797	-0.95	0.88	0.82	0.25
1214	796	-0.91	0.22	0.39	-0.1
1215	795	-1.09	0.62	0.62	0.05
1216	794	-1.01	0.85	1.01	0.29
1217	793	-0.98	0.48	0.27	-0.08
1218	792	-1.03	0.5	0.41	-0.04

1219	791	-0.36	1.14	0.11	0.3
1220	790	-1.19	0.77	0.31	-0.04
1221	789	-0.72	1.4	-0.08	0.2
1222	788	-0.86	1.35	-0.4	0.03
1223	787	-0.96	1.72	-0.28	0.16
1224	786	-1.35	1.17	-0.04	-0.08
1225	785	-1.4	1.37	-0.12	-0.05
1226	784	-0.81	1.31	0.19	0.23
1227	783	-1.07	0.25	-0.08	-0.3
1228	782	-1.04	1.62	-0.67	-0.03
1229	781	-1.22	0.44	-0.28	-0.35
1230	780	-1.17	0.53	-0.16	-0.27
1231	779	-0.99	0.19	-0.04	-0.28
1232	778	-1.28	-0.25	-0.32	-0.62
1233	777	-1.23	0.07	-0.14	-0.43
1234	776	-1.32	0.41	0.04	-0.29
1235	775	-1.22	-0.48	-0.28	-0.66
1236	774	-1.29	-0.05	0.05	-0.43
1237	773	-0.91	0.77	-0.24	-0.13
1238	772	-0.97	-0.22	-0.2	-0.46
1239	771	-1.25	-0.94	-0.51	-0.9
1240	770	-1.18	0.18	-0.24	-0.41
1241	769	-1.1	-0.77	-0.36	-0.74
1242	768	-1.11	-0.57	-0.47	-0.72
1243	767	-1.11	-0.83	-0.32	-0.75
1244	766	-1.21	-0.22	-0.12	-0.52
1245	765	-1.02	-0.2	-0.67	-0.63
1246	764	-0.74	0.71	-0.34	-0.13
1247	763	-1	0.07	0.23	-0.23
1248	762	-0.7	0.79	-0.32	-0.08
1249	761	-0.82	0.74	-0.36	-0.15
1250	760	-0.8	0.12	-0.08	-0.25
1251	759	-0.95	0.54	-0.28	-0.23
1252	758	-0.91	0.53	-0.08	-0.15

1253	757	-0.98	0.1	-0.28	-0.39
1254	756	-0.81	-0.22	-0.08	-0.37
1255	755	-1.08	-0.19	-0.43	-0.57
1256	754	-0.81	-0.02	-0.32	-0.38
1257	753	-0.62	0.27	0	-0.12
1258	752	-0.75	0.48	-0.36	-0.21
1259	751	-0.86	0.01	-0.2	-0.35
1260	750	-0.78	0.12	-0.51	-0.39
1261	749	-0.85	0.07	0.04	-0.25
1262	748	-0.86	0.01	-0.32	-0.39
1263	747	-1.05	0.77	-0.2	-0.16
1264	746	-0.78	0.82	-0.2	-0.05
1265	745	-0.47	0.01	0.39	-0.03
1266	744	-1.19	0.27	0.19	-0.24
1267	743	-0.83	0.45	0.39	0
1268	742	-1.05	0.71	0.58	0.08
1269	741	-0.59	0.59	0.43	0.14
1270	740	-0.89	1.46	0.39	0.32
1271	739	-0.71	-0.31	-0.08	-0.37
1272	738	-0.81	0.94	-0.22	-0.03
1273	737	-0.99	0.67	0.04	-0.09
1274	736	-0.75	0.16	-0.2	-0.26
1275	735	-0.67	0.56	-0.51	-0.21
1276	734	-0.45	0.62	0.07	0.08
1277	733	-0.78	1.49	-0.08	0.21
1278	732	-0.68	1.08	-0.16	0.08
1279	731	-0.64	0.68	-0.36	-0.1
1280	730	-0.41	0.04	-0.28	-0.22
1281	729	-1.03	0.68	0.43	0.03
1282	728	-0.97	1.17	0.47	0.22
1283	727	-0.6	1.66	0.35	0.47
1284	726	-0.87	-0.16	0.43	-0.2
1285	725	-0.82	1.81	0.5	0.5
1286	724	-0.95	1.46	0.5	0.34

1287	723	-0.56	1.86	0.31	0.54
1288	722	-0.61	1.52	0.19	0.37
1289	721	0.17	1.09	-0.02	0.41
1290	720	0.01	0.82	-0.28	0.19
1291	719	-0.22	1.05	-0.2	0.21
1292	718	-0.5	0.99	-0.04	0.15
1293	717	-0.69	1.37	0	0.23
1294	716	-0.46	1.2	-0.32	0.14
1295	715	-0.14	1.11	0	0.32
1296	714	-0.2	0.74	-0.04	0.16
1297	713	-0.11	0.97	0.31	0.39
1298	712	-0.27	1.26	0	0.33
1299	711	-0.24	0.82	-0.04	0.18
1300	710	0.03	0.77	0.7	0.5
1301	709	0.43	0.94	0.47	0.61
1302	708	0.13	0.86	0.86	0.61
1303	707	0.01	1.28	0.66	0.65
1304	706	1.14	1.55	0.54	1.08
1305	705	1.56	2.18	0.09	1.28
1306	704	1.33	1.23	0.21	0.92
1307	703	0.61	1.52	0.31	0.81
1308	702	0.73	1.63	0.58	0.98
1309	701	0.18	0.99	1.4	0.86
1310	700	0.39	1.11	1.01	0.84
1311	699	-0.07	1.52	0.66	0.7
1312	698	--	1.98	0.9	1.44
1313	697	--	1.63	0.97	1.3
1314	696	--	2.79	1.01	1.9
1315	695	--	1.81	1.11	1.46
1316	694	--	3.03	0.78	1.9
1317	693	--	1.92	1.33	1.62
1318	692	--	2.22	1.56	1.89
1319	691	--	2.68	1.33	2
1320	690	--	2.77	1.29	2.03

1321	689	--	3.11	1.68	2.4
1322	688	--	4.1	1.31	2.7
1323	687	--	3.17	1.25	2.21
1324	686	--	4.51	1.8	3.15
1325	685	--	2.68	1.76	2.22
1326	684	--	1.98	1.25	1.61
1327	683	--	3.98	0.74	2.36
1328	682	--	0.36	0.54	0.45
1329	681	--	2.18	0.39	1.29
1330	680	--	2.33	-0.4	0.97
1331	679	--	2.97	0.86	1.91
1332	678	0.69	3.06	1.01	1.59
1333	677	1.49	1.98	1.52	1.67
1334	676	1.52	2.82	1.13	1.83
1335	675	0.81	2.39	1.05	1.42
1336	674	-0.67	2.47	1.37	1.06
1337	673	-0.53	2.68	1.84	1.33
1338	672	-0.59	2.28	0.5	0.73
1339	671	-0.29	2.39	0.86	0.98
1340	670	-0.01	1.95	0.64	0.86
1341	669	0.4	3.78	1.05	1.74
1342	668	-0.04	1.26	0.78	0.67
1343	667	0.09	0.65	0.23	0.32
1344	666	0.62	2.88	0.47	1.32
1345	665	0.73	1.26	-0.04	0.65
1346	664	0.39	1.93	-0.16	0.72
1347	663	0.04	1.84	-0.12	0.59
1348	662	-0.16	1.34	0.04	0.41
1349	661	-0.02	1.23	-0.38	0.28
1350	660	0.07	1.31	0.07	0.49
1351	659	0.11	1.95	-0.08	0.66
1352	658	0.25	1.26	0.02	0.51
1353	657	0.33	1.31	-0.32	0.44
1354	656	0.48	1.66	-0.2	0.65

1355	655	-0.02	1.43	-0.28	0.38
1356	654	-0.04	1.35	-0.32	0.33
1357	653	-0.7	0.76	-0.28	-0.07
1358	652	-0.34	1.6	-0.43	0.28
1359	651	-0.34	1.43	0.31	0.47
1360	650	0.02	1.41	-0.79	0.21
1361	649	-0.04	1.9	-0.67	0.4
1362	648	0.63	1.03	-0.36	0.43
1363	647	0.66	1.66	-0.55	0.59
1364	646	1.45	3.49	-0.4	1.52
1365	645	0.82	2.65	-0.69	0.93
1366	644	1.94	1.92	-0.65	1.07
1367	643	-0.27	2.42	-0.86	0.43
1368	642	0.06	2.05	-0.45	0.55
1369	641	0.88	2.18	-1.02	0.68
1370	640	0.85	1.78	-0.61	0.67
1371	639	0.72	2.65	-0.36	1.01
1372	638	0.66	2.09	-0.83	0.64
1373	637	0.64	1.99	-0.4	0.74
1374	636	0.63	2.22	-0.63	0.74
1375	635	0.99	1.4	0.11	0.83
1376	634	1.42	1.63	0.04	1.03
1377	633	0.31	2.19	0.15	0.88
1378	632	0.36	1.46	0	0.61
1379	631	0.28	1.61	0.35	0.75
1380	630	-0.16	0.91	0.07	0.27
1381	629	2.46	1.63	0.47	1.52
1382	628	1.21	1.31	0	0.84
1383	627	0.47	-0.13	0.23	0.19
1384	626	0.47	2.07	0.27	0.94
1385	625	-0.21	1.03	-0.16	0.22
1386	624	0.2	2.13	0.47	0.93
1387	623	1.21	1.95	0.04	1.07
1388	622	0.83	1.69	0.35	0.96

1389	621	0.5	1.75	0.74	0.99
1390	620	1.09	1.84	0.9	1.28
1391	619	0.39	2.3	1.54	1.41
1392	618	1.21	1.46	1.66	1.44
1393	617	2.33	1.67	1.05	1.68
1394	616	1.24	1.2	0.62	1.02
1395	615	0.52	0.65	1.13	0.77
1396	614	1.04	0.88	0.74	0.89
1397	613	1.7	0.59	0.66	0.98
1398	612	1.13	1.55	0.66	1.11
1399	611	0.42	1.37	0.66	0.82
1400	610	0.55	0.73	0.74	0.67
1401	609	0.03	-0.31	0.97	0.23
1402	608	0.99	1.69	0.86	1.18
1403	607	1.04	0.48	0.35	0.62
1404	606	0.85	1.4	0.47	0.9
1405	605	0.03	0.91	0.52	0.49
1406	604	-0.46	0.68	0.94	0.39
1407	603	0.25	0.36	0.74	0.45
1408	602	0.52	-0.37	0.5	0.22
1409	601	0.06	0.33	0.78	0.39
1410	600	-0.46	0.74	0.82	0.37
1411	599	-0.59	0.79	0.58	0.26
1412	598	-0.68	0.18	0.35	-0.05
1413	597	-0.62	0.36	0.74	0.16
1414	596	-0.81	0.21	0.66	0.02
1415	595	-0.76	0.24	0.41	-0.04
1416	594	-0.79	0.24	0.62	0.02
1417	593	-0.46	-0.25	1.09	0.13
1418	592	-0.7	-0.28	1.01	0.01
1419	591	-0.62	1.95	0.86	0.73
1420	590	-0.91	0.82	0.45	0.12
1421	589	-1.14	-0.86	0	-0.67
1422	588	-1.05	0.6	0.5	0.02

1423	587	-1.1	-0.05	-0.2	-0.45
1424	586	-0.8	0.56	-0.32	-0.18
1425	585	-0.72	0.23	-0.24	-0.24
1426	584	-0.79	-0.92	-0.12	-0.61
1427	583	-0.62	-0.5	-0.08	-0.4
1428	582	-1.11	-0.43	-0.12	-0.55
1429	581	-0.72	-0.58	-0.36	-0.55
1430	580	-0.75	-0.44	-0.24	-0.48
1431	579	-1.08	-0.62	0.5	-0.4
1432	578	-0.62	-0.63	-0.36	-0.54
1433	577	-1.01	-0.39	-0.34	-0.58
1434	576	-0.8	-0.75	-0.24	-0.6
1435	575	-0.31	-0.42	-0.73	-0.49
1436	574	-0.73	-0.5	-0.51	-0.58
1437	573	-0.73	-0.78	-0.51	-0.67
1438	572	-0.91	-0.86	-0.67	-0.81
1439	571	-0.9	-0.72	-0.67	-0.76
1440	570	-0.93	-0.75	-0.71	-0.8
1441	569	-0.53	-0.8	-0.04	-0.46
1442	568	-1.08	-0.92	-0.4	-0.8
1443	567	-0.89	-0.78	-0.71	-0.79
1444	566	-0.89	-0.72	-0.59	-0.73
1445	565	-1.16	-0.6	-0.04	-0.6
1446	564	-0.8	-0.52	0.19	-0.37
1447	563	-0.73	-0.67	-0.47	-0.62
1448	562	-0.51	-0.5	-0.08	-0.37
1449	561	-0.73	-0.52	0.39	-0.29
1450	560	-0.93	-0.6	-0.16	-0.56
1451	559	-0.89	-0.75	-0.16	-0.6
1452	558	-0.83	-0.58	-0.08	-0.5
1453	557	-0.96	-0.69	0.11	-0.51
1454	556	-1.07	-0.85	-0.12	-0.68
1455	555	-0.69	-0.87	-0.28	-0.61
1456	554	-0.67	-0.83	-0.55	-0.68

1457	553	-0.8	-0.47	0	-0.43
1458	552	-0.91	-0.53	-0.63	-0.69
1459	551	-1.11	-1.2	-0.16	-0.82
1460	550	-0.71	-1.24	-0.47	-0.81
1461	549	-0.78	-0.68	-0.86	-0.77
1462	548	-0.65	-0.66	-0.86	-0.73
1463	547	-1	-0.36	-0.4	-0.58
1464	546	-0.75	-0.33	-0.92	-0.67
1465	545	-0.7	-0.45	-0.71	-0.62
1466	544	-0.93	-0.48	-0.75	-0.72
1467	543	-0.83	-0.69	-0.65	-0.72
1468	542	-0.95	-0.74	-0.85	-0.85
1469	541	-0.82	-0.73	-0.4	-0.65
1470	540	-0.89	-0.78	-0.9	-0.86
1471	539	-1.1	-0.83	-0.83	-0.92
1472	538	-0.96	-0.9	-0.94	-0.93
1473	537	-1.02	-0.89	-0.2	-0.7
1474	536	-0.9	-0.86	-0.67	-0.81
1475	535	-1.12	-0.95	-0.83	-0.96
1476	534	-1.19	-0.9	-0.79	-0.96
1477	533	-1.16	-0.95	-0.9	-1.01
1478	532	-1.14	-0.74	-1.06	-0.98
1479	531	-1.1	-0.74	-1.1	-0.98
1480	530	-0.59	-0.52	-0.73	-0.61
1481	529	-0.67	-0.58	-0.86	-0.7
1482	528	-0.52	-0.48	-0.59	-0.53
1483	527	-0.98	-0.79	-0.47	-0.75
1484	526	-0.91	-0.81	-0.75	-0.82
1485	525	-0.92	-0.69	-0.9	-0.84
1486	524	-1.15	-0.86	-0.55	-0.85
1487	523	-1.11	-0.82	-0.34	-0.75
1488	522	-1.27	-0.67	-0.83	-0.92
1489	521	-1.14	-0.73	-0.04	-0.64
1490	520	-1.1	-0.77	-0.55	-0.81

1491	519	-1.28	-0.97	-0.75	-1
1492	518	-1.04	-0.84	0.15	-0.57
1493	517	-1.02	-0.69	-0.32	-0.68
1494	516	-0.95	-0.53	-0.12	-0.53
1495	515	-0.91	-0.77	-0.12	-0.6
1496	514	-1.01	-0.56	-0.67	-0.75
1497	513	-1.13	-0.71	-0.71	-0.85
1498	512	-0.84	-0.65	-0.36	-0.62
1499	511	-1.09	-0.76	-0.28	-0.71
1500	510	-1.2	-0.93	0.04	-0.7
1501	509	-1.25	-0.88	-0.55	-0.9
1502	508	-1.2	-0.89	-0.24	-0.78
1503	507	-1.13	-0.8	-0.71	-0.88
1504	506	-1.21	-0.87	-0.75	-0.94
1505	505	-1.1	-0.72	-1.06	-0.96
1506	504	-1.13	-0.93	-1.3	-1.12
1507	503	-1.09	-0.85	-0.67	-0.87
1508	502	-1.04	-0.88	-1.1	-1.01
1509	501	-1.09	-0.89	-0.83	-0.94
1510	500	-0.92	-0.8	-0.83	-0.85
1511	499	-0.93	-0.87	-0.63	-0.81
1512	498	-1.02	-0.72	-0.51	-0.75
1513	497	-1.23	-0.93	-0.36	-0.84
1514	496	-1.05	-0.85	-0.57	-0.82
1515	495	-1	-0.89	-0.45	-0.78
1516	494	-0.46	-0.19	-0.75	-0.47
1517	493	-0.8	-0.56	-0.4	-0.58
1518	492	-0.95	-0.68	-0.24	-0.62
1519	491	-0.96	-0.74	-0.28	-0.66
1520	490	-1.07	-0.97	-0.47	-0.84
1521	489	-1.12	-1	-0.83	-0.98
1522	488	-1.17	-0.86	-0.63	-0.89
1523	487	-1.14	-0.93	-0.08	-0.72
1524	486	-1.15	-0.97	0.76	-0.45

1525	485	-1.22	-0.92	1.01	-0.37
1526	484	-1.23	-0.94	0.5	-0.55
1527	483	-0.97	-0.82	0.47	-0.44
1528	482	-0.98	-0.89	0.27	-0.53
1529	481	-0.97	-0.84	0.97	-0.28
1530	480	-0.89	-0.9	1.29	-0.17
1531	479	-0.96	-0.86	1.17	-0.22
1532	478	-0.81	-0.79	1.33	-0.09
1533	477	-0.76	-0.96	1.29	-0.15
1534	476	-0.79	-0.97	2.38	0.21
1535	475	-0.85	-0.95	3.13	0.44
1536	474	-0.79	-0.89	2.62	0.31
1537	473	-0.93	-0.68	2.15	0.18
1538	472	-1.1	-0.79	4.1	0.74
1539	471	-0.81	-0.79	1.91	0.11
1540	470	-0.86	-0.84	1.97	0.09
1541	469	-1.01	-0.92	2.46	0.18
1542	468	-0.87	-0.96	1.84	0
1543	467	-0.8	-0.81	1.64	0.01
1544	466	-1.13	-0.92	1.05	-0.33
1545	465	-1.03	-0.99	1.4	-0.2
1546	464	-1.04	-0.84	1.64	-0.08
1547	463	-0.9	-1	1.15	-0.25
1548	462	-1.01	-1.01	0.78	-0.41
1549	461	-0.9	-0.92	-0.04	-0.62
1550	460	-0.98	-1.04	0	-0.67
1551	459	-0.78	-0.87	0.82	-0.28
1552	458	-0.75	-0.89	0.76	-0.3
1553	457	-1.31	-0.96	1.09	-0.39
1554	456	-1.18	-0.89	0.97	-0.37
1555	455	-1.22	-0.85	0.58	-0.49
1556	454	-1.12	-0.87	0.47	-0.51
1557	453	-1.06	-0.88	1.54	-0.13
1558	452	-0.92	-0.62	0.97	-0.19

1559	451	-1.06	-0.47	1.4	-0.04
1560	450	-1.11	-0.63	0.82	-0.31
1561	449	-0.78	-0.64	0.68	-0.25
1562	448	-0.86	-0.58	1.44	0
1563	447	-0.98	-0.57	1.6	0.02
1564	446	-0.91	-0.8	2.11	0.13
1565	445	-0.87	-0.68	1.68	0.04
1566	444	-1.09	-0.77	2.7	0.28
1567	443	-1.14	-0.82	2.68	0.24
1568	442	-1.19	-0.93	4.1	0.66
1569	441	-1.2	-1.05	2.83	0.19
1570	440	-1.34	-0.94	3.24	0.32
1571	439	-1.24	-0.94	2.19	0
1572	438	-1.28	-1.02	1.52	-0.26
1573	437	-1.37	-1.05	2.11	-0.1
1574	436	-1.31	-1.07	2.11	-0.09
1575	435	-1.36	-1.11	2.7	0.07
1576	434	-1.24	-0.9	0.94	-0.4
1577	433	-1.14	-0.8	1.56	-0.13
1578	432	-1.22	-0.89	0.82	-0.43
1579	431	-1.34	-1.14	0.99	-0.5
1580	430	-1.32	-1.08	0.5	-0.63
1581	429	-1.25	-1.08	0.5	-0.61
1582	428	-1.35	-1.08	0.43	-0.67
1583	427	-1.29	-1.09	0.64	-0.58
1584	426	-1.29	-1.09	0.47	-0.64
1585	425	-1.29	-0.98	0.23	-0.68
1586	424	-1.29	-0.88	0.31	-0.62
1587	423	-1.32	-1.05	0.35	-0.68
1588	422	-1.29	-1.05	0	-0.78
1589	421	-1.3	-1.03	-0.2	-0.84
1590	420	-1.28	-1.09	-0.43	-0.93
1591	419	-1.28	-1.12	-0.57	-0.99
1592	418	-1.28	-1.11	0	-0.8

1593	417	-1.38	-1.12	-0.67	-1.06
1594	416	-1.3	-1.15	-1.06	-1.17
1595	415	-1.16	-1.01	-0.94	-1.04
1596	414	-1.18	-1.07	-0.63	-0.96
1597	413	-1.11	-1	-0.75	-0.95
1598	412	-1.15	-1.07	-0.9	-1.04
1599	411	-1.07	-0.95	-0.79	-0.93
1600	410	-1.06	-0.82	-0.9	-0.93
1601	409	-1.21	-0.64	-0.9	-0.92
1602	408	-1.14	-0.74	-0.94	-0.94
1603	407	-1.16	-0.75	-0.75	-0.88
1604	406	-1	-0.52	-0.94	-0.82
1605	405	-0.89	-0.92	-0.67	-0.82
1606	404	-0.92	-0.84	-0.67	-0.81
1607	403	-1.04	-0.95	-0.79	-0.93
1608	402	-1.02	-1.19	-0.67	-0.96
1609	401	-1.03	-0.86	0.11	-0.59
1610	400	-1.04	-0.67	0.33	-0.46
1611	399	-0.91	-0.95	0.07	-0.59
1612	398	-1.08	-0.81	0.35	-0.51
1613	397	-1.15	-1	0.78	-0.46
1614	396	-1.27	-0.91	1.95	-0.07
1615	395	-1.25	-0.88	0.9	-0.41
1616	394	-1.37	-0.83	1.4	-0.26
1617	393	-1.22	-0.81	1.25	-0.26
1618	392	-1.23	-0.97	0.94	-0.42
1619	391	-1.2	-0.76	0.43	-0.51
1620	390	-1.19	-0.77	0.9	-0.36
1621	389	-1.13	-0.78	0.27	-0.55
1622	388	-1.05	-1.01	0.74	-0.44
1623	387	-1.11	-0.95	0.74	-0.44
1624	386	-1.17	-1	0.35	-0.61
1625	385	-1.17	-0.93	0.27	-0.61
1626	384	-1.05	-0.89	0.21	-0.58

1627	383	-0.95	-0.84	0.27	-0.51
1628	382	-1.11	-0.88	0.35	-0.55
1629	381	-1.03	-0.86	0.66	-0.41
1630	380	-0.98	-0.75	0.39	-0.45
1631	379	-1.16	-0.83	0.23	-0.59
1632	378	-1.06	-0.86	0.11	-0.6
1633	377	-1.09	-0.83	-0.06	-0.66
1634	376	-1.15	-0.79	-0.24	-0.73
1635	375	-1.13	-0.94	-0.28	-0.78
1636	374	-1.11	-0.92	-0.2	-0.74
1637	373	-1.03	-0.94	0	-0.66
1638	372	-0.98	-0.75	-0.12	-0.62
1639	371	-1.13	-0.71	0.31	-0.51
1640	370	-0.87	-0.26	0.19	-0.31
1641	369	-0.85	-0.36	-0.28	-0.49
1642	368	-0.36	-0.09	-0.43	-0.29
1643	367	-0.63	-0.3	-0.67	-0.53
1644	366	-0.64	-0.05	-0.36	-0.35
1645	365	-0.72	-0.16	-0.16	-0.35
1646	364	-0.59	-0.48	-0.4	-0.49
1647	363	-0.89	-0.34	-0.32	-0.52
1648	362	-0.9	-0.48	-0.08	-0.49
1649	361	-0.82	-0.52	-0.24	-0.52
1650	360	-0.75	-0.55	-0.04	-0.45
1651	359	-1	0.07	-0.24	-0.39
1652	358	-1.12	-0.57	-0.55	-0.75
1653	357	-1.12	-0.7	-0.71	-0.84
1654	356	-1.15	-0.96	-1.1	-1.07
1655	355	-1.16	-0.49	-0.98	-0.88
1656	354	-1.06	-0.64	-0.92	-0.88
1657	353	-1.06	-0.72	-1	-0.93
1658	352	-1.27	-0.79	-1.06	-1.04
1659	351	-1.26	-0.83	-1.02	-1.04
1660	350	-1.18	-0.99	-0.86	-1.01

1661	349	-1.17	-0.95	-0.71	-0.94
1662	348	-1.08	-0.87	-0.79	-0.91
1663	347	-0.98	-0.76	-0.79	-0.84
1664	346	-1.14	-0.4	-0.43	-0.66
1665	345	-1.19	-0.46	-0.79	-0.81
1666	344	-1.06	-0.82	-1.22	-1.03
1667	343	-1.11	-0.63	-0.79	-0.84
1668	342	-1.04	-0.68	-0.79	-0.84
1669	341	-1.28	-0.68	-0.71	-0.89
1670	340	-0.84	-0.73	-0.9	-0.82
1671	339	-0.79	-0.79	-0.55	-0.71
1672	338	-0.99	-0.7	-0.4	-0.7
1673	337	-0.87	-0.72	-0.4	-0.66
1674	336	-1.24	-0.78	-0.36	-0.79
1675	335	-1.14	-0.94	-0.2	-0.76
1676	334	-1.15	-1.07	-0.36	-0.86
1677	333	-1.26	-1.12	-0.24	-0.87
1678	332	-1.13	-1.08	-0.12	-0.78
1679	331	-1.09	-1.17	0	-0.76
1680	330	-1.27	-1.17	-0.36	-0.93
1681	329	-1.26	-1.14	-0.43	-0.94
1682	328	-1.15	-1.18	-0.73	-1.02
1683	327	-0.99	-1.2	-0.32	-0.84
1684	326	-1.13	-1.15	-0.86	-1.05
1685	325	-0.97	-1.12	-0.86	-0.99
1686	324	-1.11	-1.14	-0.79	-1.01
1687	323	-1.09	-0.97	-0.59	-0.88
1688	322	-1.15	-1.16	-0.47	-0.93
1689	321	-1.09	-1	-0.43	-0.84
1690	320	-1.09	-0.99	-0.79	-0.96
1691	319	-1.14	-1.05	-0.28	-0.82
1692	318	-0.94	-1.09	0.04	-0.66
1693	317	-1.06	-1.07	-0.08	-0.74
1694	316	-1.03	-1.03	-0.08	-0.71

1695	315	-1.05	-1.1	-0.24	-0.8
1696	314	-0.96	-1.09	-0.28	-0.78
1697	313	-0.82	-1.18	-0.59	-0.86
1698	312	-1.06	-1.19	-0.51	-0.92
1699	311	-1.02	-1.13	-0.51	-0.89
1700	310	-1.08	-1.08	-0.63	-0.93
1701	309	-1.1	-1.16	-0.94	-1.07
1702	308	-1.18	-1.05	-1.33	-1.19
1703	307	-1.27	-1.17	-0.79	-1.08
1704	306	-1.27	-1.16	-0.83	-1.09
1705	305	-1.11	-1.13	-1.02	-1.09
1706	304	-1.17	-1.12	-0.94	-1.08
1707	303	-1.19	-1.16	-1.1	-1.15
1708	302	-1.3	-1.05	-0.79	-1.05
1709	301	-1.19	-1.07	-1.18	-1.15
1710	300	-1	-1.1	-0.86	-0.99
1711	299	-1.02	-0.94	-1.1	-1.02
1712	298	-1.13	-0.97	-0.79	-0.96
1713	297	-1.05	-1.03	-0.86	-0.98
1714	296	-0.98	-0.87	-0.67	-0.84
1715	295	-1.12	-0.99	-0.83	-0.98
1716	294	-1.14	-0.84	-0.71	-0.9
1717	293	-1.24	-0.93	-0.47	-0.88
1718	292	-1.17	-0.77	-0.47	-0.8
1719	291	-1.24	-0.65	-0.63	-0.84
1720	290	-1.28	-0.75	-0.67	-0.9
1721	289	-1.03	-0.56	-0.51	-0.7
1722	288	-1.11	-0.54	-0.94	-0.86
1723	287	-1.13	-0.63	-0.59	-0.79
1724	286	-0.79	-0.74	-0.83	-0.79
1725	285	-0.79	-0.87	-0.96	-0.87
1726	284	-1.13	-0.94	-0.94	-1
1727	283	-0.84	-0.75	-0.98	-0.86
1728	282	-1.07	-0.6	-1.26	-0.97

1729	281	-0.92	-0.37	-0.9	-0.73
1730	280	-0.88	-0.24	-1.26	-0.79
1731	279	-0.83	-0.76	-1.3	-0.96
1732	278	-0.67	-0.46	-1.33	-0.82
1733	277	-0.52	-0.86	-1.22	-0.87
1734	276	-0.79	-0.25	-1.22	-0.75
1735	275	-0.78	-0.49	-0.9	-0.72
1736	274	-0.49	-0.08	-1.14	-0.57
1737	273	-0.71	-0.24	-1.14	-0.7
1738	272	-0.81	-0.31	-0.94	-0.69
1739	271	-0.8	-0.45	-0.9	-0.72
1740	270	-0.82	-0.11	-0.79	-0.57
1741	269	-0.84	-0.58	-0.94	-0.79
1742	268	-0.72	-0.29	-1.08	-0.7
1743	267	-0.82	-0.18	-0.45	-0.48
1744	266	-0.84	0.03	-0.75	-0.52
1745	265	-0.74	0.2	-0.96	-0.5
1746	264	-0.99	-0.26	-0.92	-0.72
1747	263	-0.87	-0.06	-0.59	-0.51
1748	262	-0.54	-0.12	-0.9	-0.52
1749	261	-0.99	0.11	-0.41	-0.43
1750	260	-1.24	0.2	-0.98	-0.67
1751	259	-0.63	-0.34	-0.59	-0.52
1752	258	-0.31	-0.77	-1.06	-0.71
1753	257	-0.67	0.08	-1.14	-0.58
1754	256	-0.81	0.38	-0.98	-0.47
1755	255	-0.81	0.03	-0.94	-0.57
1756	254	-0.9	0.17	-1.14	-0.62
1757	253	-0.98	0.48	-1.18	-0.56
1758	252	-0.98	0.38	-1.14	-0.58
1759	251	-0.78	0.46	-0.51	-0.28
1760	250	-0.65	0.22	-0.9	-0.45
1761	249	-0.38	0.89	-0.67	-0.05
1762	248	-0.86	0.59	-1.14	-0.47

1763	247	-0.9	0.68	-0.9	-0.38
1764	246	-0.31	0.59	-0.86	-0.19
1765	245	-0.74	0.91	-0.55	-0.13
1766	244	-0.82	0.82	-0.9	-0.3
1767	243	-1.03	0.67	-0.16	-0.17
1768	242	-1.15	0.65	-0.51	-0.34
1769	241	-1.08	1.23	-0.47	-0.11
1770	240	-0.85	0.85	-0.47	-0.16
1771	239	-0.76	1	0	0.08
1772	238	-1.03	0.41	-0.12	-0.25
1773	237	-0.77	0.77	-0.08	-0.03
1774	236	-0.78	0.59	-0.36	-0.18
1775	235	-0.7	0.56	0	-0.05
1776	234	-0.83	0.59	-0.26	-0.17
1777	233	-0.78	0.53	0.31	0.02
1778	232	-0.63	0.39	0.5	0.09
1779	231	-0.76	-0.16	0.39	-0.18
1780	230	-0.78	0.39	0	-0.13
1781	229	-0.54	0.19	0.43	0.03
1782	228	-0.69	0.09	0.27	-0.11
1783	227	-0.65	0.54	0.35	0.08
1784	226	-0.56	0.77	0.02	0.07
1785	225	-0.84	0.85	0.39	0.13
1786	224	-0.71	0.91	0.78	0.33
1787	223	-0.71	0.35	0.35	0
1788	222	-0.55	1.14	0.27	0.29
1789	221	-0.83	0.56	-0.08	-0.12
1790	220	-0.82	0.56	0.11	-0.05
1791	219	-0.94	0.18	0.39	-0.12
1792	218	-0.82	0.54	0.72	0.14
1793	217	-0.82	0.53	0.27	-0.01
1794	216	-0.71	0.42	0.58	0.1
1795	215	-0.66	0.56	0.31	0.07
1796	214	-0.22	0.33	0.66	0.26

1797	213	-0.28	0.33	0.47	0.17
1798	212	-0.75	0.59	0.39	0.08
1799	211	-0.65	0.35	-0.04	-0.11
1800	210	-0.7	0.24	0.39	-0.03
1801	209	-0.66	0.19	0.15	-0.11
1802	208	-0.74	0.27	0.86	0.13
1803	207	-0.88	0.1	1.13	0.12
1804	206	-0.76	1.35	0.94	0.51
1805	205	-0.83	0.68	1.05	0.3
1806	204	-0.71	0.59	0.5	0.13
1807	203	-0.61	0.59	0.5	0.16
1808	202	-0.66	0.18	1.05	0.19
1809	201	-0.54	0.01	0.5	-0.01
1810	200	-0.77	0.18	0.7	0.04
1811	199	-0.68	0.04	0.58	-0.02
1812	198	-0.88	-0.02	0.58	-0.11
1813	197	-0.66	-0.25	0.35	-0.19
1814	196	-0.82	-0.37	0.19	-0.33
1815	195	-0.82	-0.48	0.23	-0.36
1816	194	-0.82	-0.28	0.54	-0.18
1817	193	-0.74	0.18	0.74	0.06
1818	192	-0.56	0.33	0.11	-0.04
1819	191	-0.59	-0.02	0.35	-0.09
1820	190	-0.98	-0.28	0.27	-0.33
1821	189	-0.66	-0.37	0.86	-0.06
1822	188	-0.87	-0.54	0.31	-0.37
1823	187	-0.41	-0.16	0.15	-0.14
1824	186	-0.66	-0.37	0.31	-0.24
1825	185	-0.64	--	0.35	-0.15
1826	184	-0.66	--	0.47	-0.1
1827	183	-0.67	--	0.15	-0.26
1828	182	-0.73	--	0	-0.37
1829	181	-0.7	--	0.35	-0.18
1830	180	-0.7	--	-0.04	-0.37

1831	179	-0.87	--	0.43	-0.22
1832	178	-0.98	--	0.04	-0.47
1833	177	-0.77	--	-0.08	-0.42
1834	176	-0.68	--	0.21	-0.23
1835	175	-0.81	--	-0.08	-0.44
1836	174	-0.94	--	-0.2	-0.57
1837	173	-0.8	0.85	-0.32	-0.09
1838	172	-0.83	-0.13	-0.08	-0.35
1839	171	-0.84	0.12	0.05	-0.22
1840	170	-0.95	-0.51	-0.32	-0.59
1841	169	-1.03	-0.1	-0.2	-0.44
1842	168	-0.9	-0.16	0.11	-0.32
1843	167	-0.65	0.1	-0.36	-0.3
1844	166	-0.77	-0.37	0.15	-0.33
1845	165	-0.89	0.09	-0.24	-0.35
1846	164	-0.36	-0.34	-0.16	-0.29
1847	163	-0.7	0.12	0.15	-0.14
1848	162	-0.03	0.33	0.04	0.11
1849	161	-0.12	0.21	-0.47	-0.13
1850	160	-0.58	-0.05	-0.2	-0.28
1851	159	-0.76	0.24	-0.2	-0.24
1852	158	-0.77	-0.39	0.19	-0.32
1853	157	-0.87	-0.54	-0.16	-0.52
1854	156	-0.61	-0.5	0	-0.37
1855	155	-0.76	-0.45	0	-0.41
1856	154	-0.73	-0.79	0.19	-0.44
1857	153	-0.46	-0.17	0.31	-0.11
1858	152	-0.8	-0.36	0.41	-0.25
1859	151	-0.44	-0.32	0.43	-0.11
1860	150	-0.76	0.17	0.45	-0.05
1861	149	-0.76	0.11	0.58	-0.02
1862	148	-0.89	0.35	0.23	-0.1
1863	147	-0.98	0.04	0.43	-0.17
1864	146	-1.13	-0.4	0.66	-0.29

1865	145	-1.03	-0.1	0.47	-0.22
1866	144	-0.95	-0.2	0.82	-0.11
1867	143	-1.08	-0.08	0.62	-0.18
1868	142	-0.96	-0.37	0.86	-0.16
1869	141	-0.63	-0.71	0.47	-0.29
1870	140	-0.7	-0.25	0.58	-0.12
1871	139	-0.71	0.03	0.72	0.01
1872	138	-0.85	-0.18	0.15	-0.29
1873	137	-1.04	-0.22	0.41	-0.29
1874	136	-0.94	-0.08	0.23	-0.26
1875	135	-1	-0.58	0.23	-0.45
1876	134	-1.09	-0.13	1.05	-0.06
1877	133	-0.93	-0.25	1.13	-0.02
1878	132	-0.98	-0.09	0.66	-0.13
1879	131	-0.73	-0.06	0.39	-0.14
1880	130	-0.85	-0.05	0.54	-0.12
1881	129	-0.85	0.12	0.5	-0.07
1882	128	-0.69	0.09	0.43	-0.06
1883	127	-0.76	-0.31	0.07	-0.33
1884	126	-0.69	-0.34	-0.16	-0.4
1885	125	-0.7	-0.49	0.47	-0.24
1886	124	-0.74	-0.4	0.43	-0.24
1887	123	-0.93	-0.47	0.5	-0.3
1888	122	-0.92	0	0.05	-0.29
1889	121	-0.89	-0.21	0.25	-0.28
1890	120	-0.35	-0.47	0.11	-0.24
1891	119	-0.6	-0.25	0.11	-0.24
1892	118	-0.74	-0.3	0.19	-0.28
1893	117	-0.66	-0.31	-0.2	-0.39
1894	116	-1.04	-0.53	-0.16	-0.58
1895	115	-0.42	-0.5	-0.16	-0.36
1896	114	-0.3	-0.58	-0.08	-0.32
1897	113	-0.85	-0.64	-0.55	-0.68
1898	112	-0.3	-0.46	-0.36	-0.37

1899	111	-0.84	-0.66	0.04	-0.49
1900	110	-0.3	-0.63	0.11	-0.27
1901	109	-0.72	-0.57	0.04	-0.42
1902	108	-0.68	-0.47	0.19	-0.32
1903	107	-0.37	-0.65	0.05	-0.32
1904	106	-0.71	-0.34	0.23	-0.27
1905	105	-0.87	-0.1	0.27	-0.23
1906	104	-0.26	-0.31	-0.51	-0.36
1907	103	0	-0.06	-0.16	-0.07
1908	102	-0.04	-0.53	0.11	-0.15
1909	101	-0.35	-0.71	1.21	0.05
1910	100	0.26	-0.42	0.47	0.1
1911	99	-0.73	-0.11	-0.04	-0.29
1912	98	-0.91	-0.25	0.47	-0.23
1913	97	-0.75	-0.09	0.97	0.04
1914	96	-0.93	0.06	0.37	-0.17
1915	95	-1	-0.4	0.04	-0.45
1916	94	-0.82	0.16	0.11	-0.18
1917	93	-1.16	0.24	0.74	-0.06
1918	92	-1.27	-0.37	-0.75	-0.8
1919	91	-1.15	-0.33	0.43	-0.35
1920	90	-0.4	-0.41	-0.16	-0.33
1921	89	-0.15	-0.23	-0.32	-0.23
1922	88	-1.2	-0.14	0.47	-0.29
1923	87	-0.88	0.06	0.39	-0.14
1924	86	-0.56	0.1	0.23	-0.07
1925	85	-0.57	0.22	0.6	0.08
1926	84	-0.8	-0.09	0.43	-0.16
1927	83	-0.79	0.19	-0.41	-0.34
1928	82	-0.57	0.17	0.04	-0.12
1929	81	-0.61	0	0	-0.21
1930	80	-1.03	-0.09	-0.04	-0.39
1931	79	-0.59	-0.01	0.11	-0.16
1932	78	-1.19	-0.13	-0.36	-0.56

1933	77	-0.48	0.34	0.39	0.08
1934	76	-0.35	0.43	0.43	0.17
1935	75	-1.01	0.16	0.23	-0.21
1936	74	-0.89	0.05	0.07	-0.25
1937	73	-0.78	0.09	0	-0.23
1938	72	-0.61	0.05	0.39	-0.06
1939	71	-1.18	0.21	0.39	-0.19
1940	70	-1.12	0.39	-0.08	-0.27
1941	69	-0.83	-0.08	0.15	-0.25
1942	68	-0.92	-0.14	-0.16	-0.41
1943	67	-0.89	0.29	-0.04	-0.21
1944	66	-0.86	-0.25	-0.32	-0.48
1945	65	-0.71	-0.43	-0.63	-0.59
1946	64	-0.88	-0.48	-0.08	-0.48
1947	63	-0.75	-0.6	0.15	-0.4
1948	62	-0.98	-0.58	-0.71	-0.76
1949	61	-0.84	-0.49	-0.47	-0.6
1950	60	-0.98	-0.4	-0.94	-0.77
1951	59	-1.03	-0.33	-0.47	-0.61
1952	58	-1.05	-0.4	-0.71	-0.72
1953	57	-1.16	-0.47	-1.02	-0.89
1954	56	-0.92	-0.44	-1.1	-0.82
1955	55	-1	-0.6	-1.33	-0.98
1956	54	-0.96	-0.3	-0.63	-0.63
1957	53	-0.98	-0.49	-0.63	-0.7
1958	52	-0.85	-0.48	-1.18	-0.83
1959	51	-1.12	-0.4	-0.32	-0.61
1960	50	-0.54	-0.65	-0.71	-0.63
1961	49	-0.56	-0.54	-1.18	-0.76
1962	48	-1	-0.68	-0.71	-0.8
1963	47	-0.72	-0.48	-0.79	-0.66
1964	46	-0.89	-0.5	-1.26	-0.88
1965	45	-0.75	-0.68	-1.02	-0.82
1966	44	-0.6	-0.61	-1.41	-0.87

1967	43	-0.83	-0.7	-1.02	-0.85
1968	42	-1.12	-0.73	-2.12	-1.32
1969	41	-1.07	-0.75	-0.86	-0.9
1970	40	-0.79	-0.61	0	-0.47
1971	39	-0.8	-0.68	-0.47	-0.65
1972	38	-0.99	-0.32	-0.24	-0.52
1973	37	-0.76	-0.48	0.31	-0.31
1974	36	-0.63	-0.56	2.19	0.33
1975	35	-0.95	-0.93	0.31	-0.52
1976	34	-0.59	-0.48	0.07	-0.33
1977	33	-1	-0.3	0.62	-0.23
1978	32	-1.08	-0.65	1.48	-0.08
1979	31	-0.91	-0.48	0.39	-0.33
1980	30	-1.04	-0.67	-0.55	-0.75
1981	29	-1.16	-0.54	-0.16	-0.62
1982	28	-0.75	-0.46	0.94	-0.09
1983	27	-1.19	-0.59	-0.94	-0.91
1984	26	-0.29	-0.49	0.7	-0.03
1985	25	-0.85	-0.18	0.54	-0.16
1986	24	-0.86	-0.34	0.15	-0.35
1987	23	-0.63	-0.49	0.39	-0.25
1988	22	-0.67	-0.27	0.7	-0.08
1989	21	-1.12	-0.24	0.23	-0.38
1990	20	-1.17	-0.22	0.54	-0.28
1991	19	-0.7	-0.19	0.54	-0.12
1992	18	-0.42	-0.16	-0.67	-0.42
1993	17	-0.57	-0.02	0.15	-0.15
1994	16	0.08	-0.1	-0.47	-0.16
1995	15	-0.5	-0.06	-0.71	-0.43
1996	14	-0.84	-0.22	0	-0.35
1997	13	0.19	-0.15	-0.08	-0.01
1998	12	-0.62	-0.16	0.39	-0.13
1999	11	-0.08	0.66	0.62	0.4
2000	10	-0.1	0.6	0.23	0.24

2001	9	0.26	0.05	-0.24	0.02
2002	8	0.28	-0.07	0	0.07
2003	7	0.23	0.79	0.39	0.47
2004	6	0.35	0.24	0.39	0.33
2005	5	0.52	0.26	-0.08	0.23
2006	4	0.14	0.18	0.15	0.16
2007	3	-0.42	-0.08	-0.16	-0.22
2008	2	-0.32	0.12	-0.04	-0.08
2009	1	-0.25	0.06	-0.32	-0.17
2010	-1	0.03	-0.08	-0.63	-0.23
2011	-2	-0.22	0.33	-0.08	0.01
2012	-3	-0.17	-0.14	0.15	-0.05
2013	-4	0.42	0	-0.71	-0.1
2014	-5	-0.39	0.17	-0.47	-0.23
2015	-6	-0.27	-0.25	-0.94	-0.49
2016	-7	-0.08	0.04	-0.47	-0.17
2017	-8	-0.32	-0.12	-0.71	-0.38
2018	-9	-0.6	-0.49	-1.02	-0.7
2019	-10	-0.46	-0.13	-1.1	-0.56
2020	-11	-0.24	-0.05	-1.33	-0.54
2021	-12	-0.46	-0.12	-0.63	-0.4
2022	-13	-0.17	-0.29	-0.63	-0.37
2023	-14	-0.33	0.01	-1.18	-0.5
2024	-15	-0.22	-0.37	-0.32	-0.3
2025	-16	0.1	0.62	-0.71	0
2026	-17	0.3	0.98	-1.18	0.03
2027	-18	-0.2	0.55	-0.71	-0.12
2028	-19	0.25	0.36	-0.79	-0.06
2029	-20	-0.13	0.56	-1.26	-0.27
2030	-21	0.04	0.56	-1.02	-0.14
2031	-22	-0.15	0.63	-1.41	-0.31
2032	-23	-0.21	0.04		-0.09
2033	-24	0.02	0.85		0.44
2034	-25	0.09	0.75		0.42

2035	-26	0.29	0.65	0.47
2036	-27	-0.27	0.97	0.35
2037	-28	0.24	0.95	0.59
2038	-29	0.09	0.29	0.19
2039	-30	-0.06	0.27	0.11
2040	-31	0.15	0.74	0.44
2041	-32	-0.22	0.29	0.03
2042	-33	-0.38	0.71	0.16
2043	-34	-0.01	0.74	0.36
2044	-35	-0.1	0.07	-0.02
2045	-36	-0.05	0.4	0.18
2046	-37	0.56	0.36	0.46
2047	-38	0.28	-0.05	0.12
2048	-39	0.03	0.26	0.14
2049	-40	-0.18	0.43	0.12
2050	-41	-0.2	0.54	0.17
2051	-42	0.43	1.02	0.73
2052	-43	0.23	-0.15	0.04
2053	-44	0.11	0.06	0.09
2054	-45	0.47	0.65	0.56
2055	-46	0.63	0.33	0.48
2056	-47	0.61	0.08	0.34
2057	-48	0.77	0.44	0.61
2058	-49	0.2	-0.15	0.03
2059	-50	0.85	0.6	0.73
2060	-51	1.03	0.88	0.95
2061	-52	1.32	1.22	1.27
2062	-53	0.76	0.92	0.84
2063	-54	0.52	0.71	0.62
2064	-55	0.28	0.44	0.36
2065	-56	0.43	0.69	0.56
2066	-57	0.69	0.13	0.41
2067	-58	0.62	0.2	0.41
2068	-59	-0.13	0.71	0.29

2069	-60	0.54	0.33	0.43
2070	-61	0.66	0.43	0.55
2071	-62	0.74	0.36	0.55
2072	-63	0.71	0.73	0.72
2073	-64	0.96	0.59	0.78
2074	-65	1.26	0.77	1.01
2075	-66	0.83	0.17	0.5
2076	-67	0.98	0.1	0.54
2077	-68	0.66	0.01	0.34
2078	-69	0.99	0.26	0.62
2079	-70	0.8	0.35	0.57
2080	-71	-0.43	0.26	-0.09
2081	-72	0.77	0.01	0.39
2082	-73	-0.24	-0.5	-0.37
2083	-74	1.21	0.01	0.61
2084	-75	1.01	-0.02	0.5
2085	-76	0.74	-0.18	0.28
2086	-77	0.31	-0.22	0.04
2087	-78	1.51	-0.37	0.57
2088	-79	0.69	1.2	0.94
2089	-80	1.07	0.36	0.71
2090	-81	0.52	0.65	0.58
2091	-82	0.77	0.5	0.64
2092	-83	0.66	0.62	0.64
2093	-84	0.5	-0.19	0.15
2094	-85	-0.05	0.94	0.44
2095	-86	0.44	-0.14	0.15
2096	-87	0.55	0.56	0.56
2097	-88	0.69	0.88	0.79
2098	-89	0.25	1.66	0.96
2099	-90	0.9	0.56	0.73
2100	-91	0.74	0.04	0.39
2101	-92	0.58	0.62	0.6
2102	-93	1.54	0.85	1.19

2103	-94	1.07		1.26	1.16
2104	-95	0.82		2.36	1.59
2105	-96	0.82		2.24	1.53
2106	-97	0.14		2.85	1.5
2107	-98	0.79		2.36	1.58
2108	-99	0.96		2.16	1.56
2109	-100	0.99		1.75	1.37
2110	-101	0.99		1.35	1.17
2111	-102	0.06		1.46	0.76
2112	-103	0.66		1.34	1
2113	-104	-0.31		0.88	0.29
2114	-105	0.92		1.17	1.05
2115	-106	0.48		0.94	0.71
2116	-107	0		0.97	0.49
2117	-108	0.1		0.36	0.23
2118	-109	0.51		0.3	0.41
2119	-110	-0.17		1.4	0.61
2120	-111	0.61		1.9	1.25
2121	-112	0.31		1.63	0.97
2122	-113	0.23		4.41	2.32
2123	-114	0.34		4.71	2.52
2124	-115	0.44		4.36	2.4
2125	-116	0.63		0.82	0.73
2126	-117	0.17			0.17
2127	-118	-0.08			-0.08
2128	-119	2.85			2.85
2129	-120	1.75	--		1.75
2130	-121	0.88	--		0.88
2131	-122	0.06	--		0.06
2132	-123	1.97	--		1.97
2133	-124	0.44	--		0.44
2134	-125	-0.05	--		-0.05
2135	-126	-0.16	--		-0.16
2136	-127	0.25	--		0.25

2137	-128	0.39	--	0.39
2138	-129	-0.13	--	-0.13
2139	-130	-0.19	--	-0.19
2140	-131	0.14	--	0.14
2141	-132	-0.38	--	-0.38
2142	-133	-0.11	--	-0.11
2143	-134	-0.19	--	-0.19
2144	-135	-0.13	--	-0.13
2145	-136	0.63	--	0.63
2146	-137	-0.27	--	-0.27
2147	-138	0.28	--	0.28
2148	-139	0.85	--	0.85
2149	-140	0.2	--	0.2
2150	-141	0.26	--	0.26
2151	-142	0.11	--	0.11
2152	-143	0.28	--	0.28
2153	-144	-0.08	--	-0.08
2154	-145	-0.22	--	-0.22
2155	-146	0.34	--	0.34
2156	-147	-0.3	--	-0.3
2157	-148	0.23	--	0.23
2158	-149	0.77	--	0.77
2159	-150	0.34	--	0.34
2160	-151	0.11	--	0.11
2161	-152	-0.11	--	-0.11
2162	-153	0.36	--	0.36
2163	-154	-0.46	--	-0.46
2164	-155	0.17	--	0.17
2165	-156	0.14	--	0.14
2166	-157	0.93	--	0.93
2167	-158	0.61	--	0.61
2168	-159	0.5	--	0.5
2169	-160	-0.08	--	-0.08
2170	-161	-0.1	--	-0.1

2171	-162	0	--	0
2172	-163	0.17	--	0.17
2173	-164	0.17	--	0.17
2174	-165	0.09	--	0.09
2175	-166	1.32	--	1.32
2176	-167	1.04	--	1.04
2177	-168	0.69	--	0.69
2178	-169	0.69	--	0.69
2179	-170	-0.35	--	-0.35
2180	-171	0.42	--	0.42
2181	-172	-0.43	--	-0.43
2182	-173	-0.1	--	-0.1
2183	-174	0.31	--	0.31
2184	-175	-0.05	--	-0.05
2185	-176	0.52	--	0.52
2186	-177	0.39	--	0.39
2187	-178	0.42	--	0.42
2188	-179	0.69	--	0.69
2189	-180	0.74	--	0.74
2190	-181	-0.11	--	-0.11
2191	-182	0.31	--	0.31
2192	-183	0.2	--	0.2
2193	-184	0.14	--	0.14
2194	-185	-0.08	--	-0.08
2195	-186	0.93	--	0.93
2196	-187	0.22	--	0.22
2197	-188	0.64	--	0.64
2198	-189	0.28	--	0.28
2199	-190	0.11	--	0.11
2200	-191	0.32	--	0.32
2201	-192	0.14	--	0.14
2202	-193	0.11	--	0.11
2203	-194	-0.09	--	-0.09
2204	-195	0.21	--	0.21

2205	-196	0.06	--	0.06
2206	-197	0.43	--	0.43
2207	-198	0.37	--	0.37
2208	-199	0.84	--	0.84
2209	-200	0.47	--	0.47
2210	-201	0.77	--	0.77
2211	-202	0.69	--	0.69
2212	-203	-0.19	--	-0.19
2213	-204	0.2	--	0.2
2214	-205	0.36	--	0.36
2215	-206	-0.13	--	-0.13
2216	-207	0.25	--	0.25
2217	-208	-0.62	--	-0.62
2218	-209	0.61	--	0.61
2219	-210	0.5	--	0.5
2220	-211	1.07	--	1.07
2221	-212	0.93	--	0.93
2222	-213	0.47	--	0.47
2223	-214	0.96	--	0.96
2224	-215	1.07	--	1.07
2225	-216	0.93	--	0.93
2226	-217	1.26	--	1.26
2227	-218	0.03	--	0.03
2228	-219	0.22	--	0.22
2229	-220	0.37	--	0.37
2230	-221	0.44	--	0.44
2231	-222	0.55	--	0.55
2232	-223	-0.21	--	-0.21
2233	-224	0.77	--	0.77
2234	-225	0.14	--	0.14
2235	-226	0.55	--	0.55
2236	-227	0.39	--	0.39
2237	-228	0.17	--	0.17
2238	-229	-0.08	--	-0.08

2239	-230	0.14	--	0.14
2240	-231	1.26	--	1.26
2241	-232	0.09	--	0.09
2242	-233	-0.02	--	-0.02
2243	-234	-0.15	--	-0.15
2244	-235	-0.31	--	-0.31
2245	-236	-0.38	--	-0.38
2246	-237	-0.48	--	-0.48
2247	-238	0.43	--	0.43
2248	-239	0.61	--	0.61
2249	-240	-0.53	--	-0.53
2250	-241	0.03	--	0.03
2251	-242	0.14	--	0.14
2252	-243	0.36	--	0.36
2253	-244	0.24	--	0.24
2254	-245	0.13	--	0.13
2255	-246	0.14	--	0.14
2256	-247	-0.18	--	-0.18
2257	-248	-0.15	--	-0.15
2258	-249	-0.51	--	-0.51
2259	-250	-0.17	--	-0.17
2260	-251	-0.37	--	-0.37
2261	-252	-0.25	--	-0.25
2262	-253	-0.42	--	-0.42
2263	-254	-0.09	--	-0.09
2264	-255	-0.48	--	-0.48
2265	-256	-0.48	--	-0.48
2266	-257	-0.21	--	-0.21
2267	-258	-0.69	--	-0.69
2268	-259	-0.43	--	-0.43
2269	-260	-0.65	--	-0.65
2270	-261	-0.66	--	-0.66
2271	-262	0	--	0
2272	-263	-0.23	--	-0.23

2273	-264	-0.76	--	-0.76
2274	-265	-0.62	--	-0.62
2275	-266	-0.01	--	-0.01
2276	-267	0.27	--	0.27
2277	-268	0.93	--	0.93
2278	-269	-0.16	--	-0.16
2279	-270	0.28	--	0.28
2280	-271	0.08	--	0.08
2281	-272	0.58	--	0.58
2282	-273	0.17	--	0.17
2283	-274	0.25	--	0.25
2284	-275	-0.43	--	-0.43
2285	-276	0.47	--	0.47
2286	-277	-0.05	--	-0.05
2287	-278	0.03	--	0.03
2288	-279	0.17	--	0.17
2289	-280	0	--	0
2290	-281	0.93	--	0.93
2291	-282	0.88	--	0.88
2292	-283	0.23	--	0.23
2293	-284	0.53	--	0.53
2294	-285	-0.03	--	-0.03
2295	-286	-0.24	--	-0.24
2296	-287	0.25	--	0.25
2297	-288	0.11	--	0.11
2298	-289	-0.16	--	-0.16
2299	-290	0.42	--	0.42
2300	-291	0.91	--	0.91
2301	-292	0.36	--	0.36
2302	-293	-0.43	--	-0.43
2303	-294	0.36	--	0.36
2304	-295	0.22	--	0.22
2305	-296	0.96	--	0.96
2306	-297	1.02	--	1.02

2307	-298	0.47	--	0.47
2308	-299	-0.24	--	-0.24
2309	-300	0.03	--	0.03
2310	-301	0.85	--	0.85
2311	-302	0.25	--	0.25
2312	-303	-0.02	--	-0.02
2313	-304	0.5	--	0.5
2314	-305	0.06	--	0.06
2315	-306	1.24	--	1.24
2316	-307	1.15	--	1.15
2317	-308	1.09	--	1.09
2318	-309	1.02	--	1.02
2319	-310	2.22	--	2.22
2320	-311	0.91	--	0.91
2321	-312	1.07	--	1.07
2322	-313	2	--	2
2323	-314	0.91	--	0.91
2324	-315	1.78	--	1.78
2325	-316	0.42	--	0.42
2326	-317	3.01	--	3.01
2327	-318	0.03	--	0.03
2328	-319	1.56	--	1.56
2329	-320	0.31	--	0.31
2330	-321	0.58	--	0.58
2331	-322	1.02	--	1.02
2332	-323	1.21	--	1.21
2333	-324	1.7	--	1.7
2334	-325	0.93	--	0.93
2335	-326	0.91	--	0.91
2336	-327	1.91	--	1.91
2337	-328	2.05	--	2.05
2338	-329	0.93	--	0.93
2339	-330	0.63	--	0.63
2340	-331	1.32	--	1.32

2341	-332	0.74	--		0.74
2342	-333	1.59	--		1.59
2343	-334	2.36	--		2.36
2344	-335	2.49	--		2.49
2345	-336	3.37	--		3.37
2346	-337	2.38	--		2.38
2347	-338	0.31	--		0.31
2348	-339	0.64	--		0.64
2349	-340	0.41	--		0.41
2350	-341	1.04	--		1.04
2351	-342	0.53	--		0.53
2352	-343	0.6	--		0.6
2353	-344	1.18			1.18
2354	-345	1.89			1.89
2355	-346	0.79			0.79
2356	-347	1.78		-1.23	0.27
2357	-348	1.59		-1.31	0.14
2358	-349	2.85		-1.33	0.76
2359	-350	1.4		-1.24	0.08
2360	-351	1.15		-1.05	0.05
2361	-352	1.4		-1.27	0.06
2362	-353	0.66		-1.26	-0.3
2363	-354	0.91		-0.81	0.05
2364	-355	0.34		-1.21	-0.44
2365	-356	0.69		-1.16	-0.24
2366	-357	0.47		-1.01	-0.27
2367	-358	1.16		-0.94	0.11
2368	-359	0.52		-0.91	-0.19
2369	-360	1.1		-1.12	-0.01
2370	-361	0.55		-1	-0.22
2371	-362	0.58		-1.09	-0.25
2372	-363	-0.02		-1.21	-0.62
2373	-364	0.55		-1.19	-0.32
2374	-365	0.11		-1.15	-0.52

2375	-366	0.47	-1.1	-0.32
2376	-367	0.01	-1.04	-0.52
2377	-368	0.11	-1.16	-0.53
2378	-369	0.15	-0.83	-0.34
2379	-370	0	-0.76	-0.38
2380	-371	-0.26	-1.28	-0.77
2381	-372	-0.44	-1.02	-0.73
2382	-373	-0.11	-1.01	-0.56
2383	-374	-0.16	-0.7	-0.43
2384	-375	-0.16	-0.76	-0.46
2385	-376	-0.19	-0.85	-0.52
2386	-377	-0.24	-1.05	-0.65
2387	-378	0.49	-1.28	-0.4
2388	-379	0.22	-1.27	-0.53
2389	-380	0.12	-1.3	-0.59
2390	-381	-0.14	-1.35	-0.74
2391	-382	-0.16	-1.25	-0.71
2392	-383	-0.53	-1.02	-0.78
2393	-384	-0.19	-0.69	-0.44
2394	-385	-0.06	-0.97	-0.52
2395	-386	0	-1.01	-0.5
2396	-387	-0.11	-0.88	-0.5
2397	-388	-0.07	-0.72	-0.39
2398	-389	0.3	-0.45	-0.08
2399	-390	-0.03	0.47	0.22
2400	-391	-0.71	-0.51	-0.61
2401	-392	-0.33	-0.16	-0.25
2402	-393	-0.46	1.11	0.33
2403	-394	0.13	0.24	0.19
2404	-395	0.2	0.27	0.23
2405	-396	-0.49	0.54	0.02
2406	-397	-0.27	0.07	-0.1
2407	-398	-0.23	0.16	-0.04
2408	-399	-0.03	-0.02	-0.03

2409	-400	0.16	-0.14	0.01
2410	-401	-0.38	0.16	-0.11
2411	-402	-0.44	0.07	-0.18
2412	-403	-0.21	0.27	0.03
2413	-404	-0.52	0.24	-0.14
2414	-405	-0.12	0.27	0.08
2415	-406	-0.13	-0.1	-0.12
2416	-407	0.16	0.12	0.14
2417	-408	0.55	-0.01	0.27
2418	-409	0.53	0.07	0.3
2419	-410	0.27	0.07	0.17
2420	-411	0.27	0.27	0.27
2421	-412	0.26	0.01	0.13
2422	-413	0.56	0.04	0.3
2423	-414	0.68	-0.57	0.05
2424	-415	0.3	-0.16	0.07
2425	-416	0.08	-0.08	0
2426	-417	0.96	0.04	0.5
2427	-418	-0.51	-0.16	-0.34
2428	-419	-0.51	0.33	-0.09
2429	-420	-0.38	-0.48	-0.43
2430	-421	0.11	0.01	0.06
2431	-422	0.06	0.77	0.41
2432	-423	-0.21	0.5	0.15
2433	-424	-0.51	1	0.24
2434	-425	-0.1	0.65	0.27
2435	-426	-0.27	0.1	-0.09
2436	-427	-0.59	1.41	0.41
2437	-428	-0.31	1.11	0.4
2438	-429	-0.5	-0.16	-0.33
2439	-430	-0.27	-0.54	-0.41
2440	-431	0.13	-0.68	-0.27
2441	-432	0.24	-0.63	-0.2
2442	-433	0.08	1.37	0.73

2443	-434	0.08	1.09	0.58
2444	-435	0.35	1.26	0.8
2445	-436	0.77	0.04	0.4
2446	-437	0.82	-0.85	-0.01
2447	-438	0.14	-1	-0.43
2448	-439	0.58	-1.03	-0.23
2449	-440	0.58	-1.1	-0.26
2450	-441	1.07	0.39	0.73
2451	-442	0.25	1.05	0.65
2452	-443	1.04	0.71	0.88
2453	-444	0.08	-0.07	0.01
2454	-445	0.28	0.73	0.51
2455	-446	0.77	1.66	1.21
2456	-447	0.72	1.14	0.93
2457	-448	0.5	1.89	1.19
2458	-449	0.06	0.65	0.35
2459	-450	1.56	2.16	1.86
2460	-451	-0.35	1.09	0.37
2461	-452	-0.24	3.49	1.63
2462	-453	1.62	3.14	2.38
2463	-454	3.26	1.17	2.21
2464	-455	4.08	3.37	3.72
2465	-456	4.81	3.09	3.95
2466	-457	6.78	2.33	4.55
2467	-458	3.01	4.77	3.89
2468	-459	3.01	4.6	3.8
2469	-460	3.23	2.33	2.78
2470	-461	4.32	3.72	4.02
2471	-462	4.59	3.67	4.13
2472	-463	4.76	4.19	4.47
2473	-464	2.33	4.83	3.58
2474	-465	2.33	3.78	3.05
2475	-466	3.42	5.11	4.27
2476	-467	1.73	7.26	4.49

2477	-468	5.14	4.24	4.69
2478	-469	6.13	7.26	6.69
2479	-470	0.11	4.53	2.32
2480	-471	0.88	3.55	2.21
2481	-472	0.28	2.45	1.36
2482	-473	0.63	1.93	1.28
2483	-474	1.01	1.63	1.32
2484	-475	0.11	1.17	0.64
2485	-476	0.28	2.68	1.48
2486	-477	0.17	2.85	1.51
2487	-478	2.43	1.87	2.15
2488	-479	2.55	1.69	2.12
2489	-480	1.15	0.94	1.05
2490	-481	4.35	1.28	2.81
2491	-482	1.16	1.58	1.37
2492	-483	0.17	2.33	1.25
2493	-484	-0.54	2.45	0.95
2494	-485	2.22	2.16	2.19
2495	-486	-0.16	1.75	0.8
2496	-487	1.34	1.69	1.52
2497	-488	0.66	1.98	1.32
2498	-489	2.14	1.95	2.05
2499	-490	0.28	1.46	0.87
2500	-491	2.14	1.98	2.06
2501	-492	1.27	1.92	1.59
2502	-493	2.09	1.35	1.72
2503	-494	0.77	2.21	1.49
2504	-495	0.33	2.19	1.26
2505	-496	0.9	1.34	1.12
2506	-497	1.45	1.26	1.35
2507	-498	0.28	1.86	1.07
2508	-499	-0.02	0.36	0.17
2509	-500	1.7	1.23	1.46
2510	-501	-0.08	0.62	0.27

2511	-502	0.06	2.53	1.3
2512	-503	-0.13	0.94	0.4
2513	-504	0.11	1.09	0.6
2514	-505	-0.21	0.48	0.13
2515	-506	-0.24	0.74	0.25
2516	-507	-0.38	1.2	0.41
2517	-508	-0.4	2.65	1.12
2518	-509	-0.54	1.4	0.43
2519	-510	-0.08	0.43	0.18
2520	-511	-0.11	-0.28	-0.19
2521	-512	-0.76	0.9	0.07
2522	-513	-0.54	0.58	0.02
2523	-514	-0.11	0.85	0.37
2524	-515	-0.46	0.36	-0.05
2525	-516	0	0.79	0.4
2526	-517	-0.46	0.66	0.1
2527	-518	-0.24	1.21	0.49
2528	-519	-0.24	0.51	0.13
2529	-520	-0.6	0.85	0.12
2530	-521	-0.41	0.31	-0.05
2531	-522	-0.54	0.27	-0.14
2532	-523	-0.19	-0.3	-0.25
2533	-524	-0.33	-0.46	-0.39
2534	-525	-0.86	-0.37	-0.61
2535	-526	-0.73	-0.08	-0.4
2536	-527	-0.38	-0.25	-0.32
2537	-528	0.12	-0.22	-0.05
2538	-529	-0.47	-0.28	-0.38
2539	-530	-0.87	0.44	-0.21
2540	-531	-0.75	0.18	-0.29
2541	-532	-0.38	0.13	-0.13
2542	-533	-0.56	-0.19	-0.38
2543	-534	-1	-0.15	-0.57
2544	-535	-0.6	0.14	-0.23

2545	-536	-0.73	-0.36	-0.55
2546	-537	-0.95	-0.53	-0.74
2547	-538	-0.75	-0.45	-0.6
2548	-539	-0.37	-0.36	-0.36
2549	-540	-0.4	-0.55	-0.48
2550	-541	-0.67	-0.88	-0.78
2551	-542	-0.49	-0.55	-0.52
2552	-543	-0.24	-0.41	-0.33
2553	-544	-0.38	0.25	-0.06
2554	-545	-0.52	-0.56	-0.54
2555	-546	-0.78	-0.75	-0.77
2556	-547	-0.29	-0.56	-0.43
2557	-548	-0.44	-0.61	-0.53
2558	-549	-0.67	-0.7	-0.69
2559	-550	-0.78	-0.62	-0.7
2560	-551	-0.51	-0.61	-0.56
2561	-552	-0.51	-0.85	-0.68
2562	-553	-0.24	-0.78	-0.51
2563	-554	-0.45	-0.77	-0.61
2564	-555	-1.02	-0.89	-0.95
2565	-556	-0.51	-0.52	-0.52
2566	-557	-0.56	-1.12	-0.84
2567	-558	-0.95	-0.63	-0.79
2568	-559	-0.56	-0.55	-0.56
2569	-560	-0.64	-0.25	-0.45
2570	-561	-0.4	-0.55	-0.48
2571	-562	-0.71	-0.67	-0.69
2572	-563	-0.58	-0.31	-0.44
2573	-564	-0.93	0.07	-0.43
2574	-565	-0.91	-0.18	-0.55
2575	-566	-0.91	-0.91	-0.91
2576	-567	-0.93	-0.99	-0.96
2577	-568	-0.92	-0.69	-0.81
2578	-569	-0.91	-0.77	-0.84

2579	-570	-1.15	-0.88	-1.02
2580	-571	-0.99	-0.92	-0.95
2581	-572	-0.48	-1.01	-0.75
2582	-573	-0.62	-0.89	-0.76
2583	-574	-0.67	-0.94	-0.81
2584	-575	-0.79	-0.96	-0.87
2585	-576	-0.58	-0.94	-0.76
2586	-577	-0.69	-0.71	-0.7
2587	-578	-0.72	-0.6	-0.66
2588	-579	-0.7	-0.67	-0.69
2589	-580	-0.92	-0.85	-0.88
2590	-581	-0.59	-0.58	-0.59
2591	-582	-0.81	-0.92	-0.86
2592	-583	-0.84	-0.73	-0.78
2593	-584	-0.91	-0.62	-0.76
2594	-585	-0.79	-0.57	-0.68
2595	-586	-0.39	-0.79	-0.59
2596	-587	-0.93	-0.87	-0.9
2597	-588	-0.5	-0.55	-0.53
2598	-589	-0.57	-0.64	-0.61
2599	-590	-1.03	-0.87	-0.95
2600	-591	-0.94	-0.85	-0.89
2601	-592	-0.15	-0.97	-0.56
2602	-593	-0.28	-0.78	-0.53
2603	-594	-0.94	-1.03	-0.98
2604	-595	-0.78	-0.53	-0.65
2605	-596	-0.51	-0.7	-0.61
2606	-597	-0.16	-0.81	-0.49
2607	-598	-0.78	-0.64	-0.71
2608	-599	-0.28	-0.63	-0.46
2609	-600	-0.54	-0.93	-0.74
2610	-601	-0.54	-0.64	-0.59
2611	-602	-0.72	-0.62	-0.67
2612	-603	-0.85	-0.84	-0.84

2613	-604	-0.14	-0.82	-0.48
2614	-605	-0.04	-0.78	-0.41
2615	-606	-0.36	-0.48	-0.42
2616	-607	-0.12	-0.49	-0.31
2617	-608	-0.54	-0.65	-0.59
2618	-609	-0.29	-0.79	-0.54
2619	-610	-0.52	-0.88	-0.7
2620	-611	-0.43	-0.73	-0.58
2621	-612	-0.65	-1.1	-0.88
2622	-613	-0.44	-0.71	-0.58
2623	-614	-0.42	-0.94	-0.68
2624	-615	-0.56	-0.77	-0.66
2625	-616	-0.37	-0.44	-0.4
2626	-617	-0.2	-0.81	-0.51
2627	-618	-0.19	-0.81	-0.5
2628	-619	0.13	-0.62	-0.24
2629	-620	-0.26	-0.36	-0.31
2630	-621	-0.18	-0.7	-0.44
2631	-622	-0.47	-0.2	-0.33
2632	-623	1.24	-0.14	0.55
2633	-624	-0.02	0.35	0.17
2634	-625	-0.49	-0.34	-0.42
2635	-626	-0.06	0.17	0.06
2636	-627	0.07	0.02	0.04
2637	-628	0	0.94	0.47
2638	-629	-0.92	-0.87	-0.89
2639	-630	-0.36	-0.89	-0.62
2640	-631	-0.36	-1.01	-0.68
2641	-632	-0.95	-0.98	-0.96
2642	-633	-0.23	-0.33	-0.28
2643	-634	-0.39	-0.89	-0.64
2644	-635	-1.01	-1.03	-1.02
2645	-636	-0.14	-0.81	-0.48
2646	-637	0.01	-0.97	-0.48

2647	-638	-0.44	-1.04	-0.74
2648	-639	0.35	-0.72	-0.19
2649	-640	0.01	-0.95	-0.47
2650	-641	-0.21	-0.93	-0.57
2651	-642	-0.7	-1.16	-0.93
2652	-643	-0.38	-0.62	-0.5
2653	-644	-0.51	0.08	-0.22
2654	-645	-0.64	-0.89	-0.77
2655	-646	-0.46	-0.42	-0.44
2656	-647	-0.69	0.04	-0.32
2657	-648	-0.49	-0.4	-0.45
2658	-649	-0.54	-0.65	-0.6
2659	-650	-0.38	-0.16	-0.27
2660	-651	-0.83	0.38	-0.22
2661	-652	-0.78	0.48	-0.15
2662	-653	-0.43	0.79	0.18
2663	-654	-0.39	1.06	0.33
2664	-655	-0.61	-0.15	-0.38
2665	-656	-0.26	-0.15	-0.2
2666	-657	-0.07	-0.25	-0.16
2667	-658	-0.42	0.56	0.07
2668	-659	-0.82	1	0.09
2669	-660	-0.81	0.67	-0.07
2670	-661	-0.98	0.36	-0.31
2671	-662	-1.03	0.59	-0.22
2672	-663	-1.01	0.47	-0.27
2673	-664	-0.71	0.74	0.01
2674	-665	-0.62	0.88	0.13
2675	-666	-0.94	0.85	-0.05
2676	-667	-0.11	0.16	0.03
2677	-668	-0.45	0.59	0.07
2678	-669	0.01	0.73	0.37
2679	-670	-0.1	1.41	0.65
2680	-671	-0.28	1.43	0.57

2681	-672	-0.05	1.03	0.49
2682	-673	-0.11	-0.02	-0.06
2683	-674	-0.59	0.59	0
2684	-675	1.15	-0.02	0.56
2685	-676	0.44	-0.05	0.2
2686	-677	1.02	0.16	0.59
2687	-678	-0.32	0.3	-0.01
2688	-679	-0.19	0.3	0.05
2689	-680	0.39	0.48	0.43
2690	-681	0.06	0.27	0.17
2691	-682	1.86	0.99	1.43
2692	-683	1.94	0.07	1.01
2693	-684	-0.13	0.56	0.22
2694	-685	0.52	-0.75	-0.11
2695	-686	1.18	-0.25	0.46
2696	-687	1.51	0.39	0.95
2697	-688	2.16	-0.31	0.92
2698	-689	0.53	-0.05	0.24
2699	-690	1.07	-0.48	0.29
2700	-691	2.08	-0.57	0.76
2701	-692	2.71	-0.37	1.17
2702	-693	2.68	0.33	1.51
2703	-694	1.92	-0.02	0.95
2704	-695	3.93	0.36	2.15
2705	-696	1.13	-0.07	0.53
2706	-697	1.15	0.15	0.65
2707	-698	1.07	0.07	0.57
2708	-699	0.31	0.44	0.38
2709	-700	1.4	0.36	0.88
2710	-701	0.82	-0.35	0.24
2711	-702	0.03	0.66	0.35
2712	-703	0.17	0.73	0.45
2713	-704	1.78	0.79	1.29
2714	-705	2	0.22	1.11

2715	-706	2.63	0.71	1.67
2716	-707	0.36	-0.05	0.15
2717	-708	0.55	0.14	0.34
2718	-709	0.66	0.03	0.35
2719	-710	2.52	0.09	1.31
2720	-711	2.11	-0.01	1.05
2721	-712	1.26	0.04	0.65
2722	-713	1.02	-0.05	0.49
2723	-714	0.93	0.16	0.55
2724	-715	0.28	0.16	0.22
2725	-716	1.32	0.3	0.81
2726	-717	1.51	0.5	1.01
2727	-718	0.82	0.68	0.75
2728	-719	1.18	0.44	0.81
2729	-720	0.2	-0.45	-0.13
2730	-721	-0.4	0.18	-0.11
2731	-722	-0.1	-0.54	-0.32
2732	-723	1.37	-0.63	0.37
2733	-724	0.61	-0.54	0.04
2734	-725	0.66	-0.42	0.12
2735	-726	0.5	-0.48	0.01
2736	-727	1.02	0.16	0.59
2737	-728	0.39	-0.16	0.11
2738	-729	0.03	-0.22	-0.09
2739	-730	-0.11	-0.22	-0.16
2740	-731	0.88	-0.25	0.31
2741	-732	0.69	-0.37	0.16
2742	-733	0.61	-0.45	0.08
2743	-734	0.03	-0.51	-0.24
2744	-735	0.39	-0.37	0.01
2745	-736	0.61	-0.86	-0.13
2746	-737	0.23	-0.6	-0.19
2747	-738	-0.19	-0.71	-0.45
2748	-739	-0.35	-0.37	-0.36

2749	-740	0.23	-0.05	0.09
2750	-741	1.07	0.27	0.67
2751	-742	0.06	-0.25	-0.09
2752	-743	0.55	-0.46	0.05
2753	-744	0.42	-0.6	-0.09
2754	-745	-0.13	-0.14	-0.13
2755	-746	0.55	-0.48	0.03
2756	-747	-0.08	-0.33	-0.21
2757	-748	-0.48	-0.56	-0.52
2758	-749	0	-0.46	-0.23
2759	-750	-0.05	-0.75	-0.4
2760	-751	-0.08	-0.76	-0.42
2761	-752	0.69	-0.67	0.01
2762	-753	-0.4	-0.5	-0.45
2763	-754	0.39	-0.46	-0.04
2764	-755	0.42	-0.57	-0.08
2765	-756	0.06	-0.48	-0.21
2766	-757	1.13	-0.8	0.16
2767	-758	0.17	-0.76	-0.29
2768	-759	3.01	-0.4	1.31
2769	-760	1.97	-0.49	0.74
2770	-761	0.88	-0.79	0.05
2771	-762	0.66	-0.3	0.18
2772	-763	0.25	-0.3	-0.02
2773	-764	1.45	-0.63	0.41
2774	-765	1.1	-0.74	0.18
2775	-766	1.83	-0.61	0.61
2776	-767	1.56	-0.78	0.39
2777	-768	0.52	-1.12	-0.3
2778	-769	-0.4	-1.13	-0.77
2779	-770	-0.65	-1.04	-0.85
2780	-771	-0.48	-0.78	-0.63
2781	-772	-0.84	-0.8	-0.82
2782	-773	-0.68	-0.65	-0.67

2783	-774	-0.57	-0.48	-0.53
2784	-775	-0.46	-1.04	-0.75
2785	-776	-0.24	-0.92	-0.58
2786	-777	0.34	-0.79	-0.23
2787	-778	1.23	-0.57	0.33
2788	-779	1.18	-0.75	0.21
2789	-780	-0.16	-0.76	-0.46
2790	-781	-0.43	-1.01	-0.72
2791	-782	-0.13	-1.04	-0.59
2792	-783	-0.32	-0.88	-0.6
2793	-784	-0.21	-0.88	-0.55
2794	-785	-0.57	-1	-0.78
2795	-786	-0.16	-0.94	-0.55
2796	-787	-0.35	-0.68	-0.51
2797	-788	0	-0.24	-0.12
2798	-789	-0.65	-0.75	-0.7
2799	-790	-0.4	-0.51	-0.46
2800	-791	-0.41	-0.37	-0.39
2801	-792	-0.37	-0.71	-0.54
2802	-793	-0.27	0.87	0.3
2803	-794	0.23	-0.34	-0.06
2804	-795	0.01		0.01
2805	-796	0.03		0.03
2806	-797	-0.21		-0.21
2807	-798	0.69		0.69
2808	-799	0.39		0.39
2809	-800	2.52		2.52
2810	-801	0.61		0.61
2811	-802	0.5		0.5
2812	-803	0.85		0.85
2813	-804	0.44		0.44
2814	-805	0.44		0.44
2815	-806	1.35		1.35
2816	-807	1.65		1.65

2817	-808	0.6	--	0.6
2818	-809	0.85	--	0.85
2819	-810	1.35	--	1.35
2820	-811	-0.08	--	-0.08
2821	-812	0.03	--	0.03
2822	-813	0.39	--	0.39
2823	-814	-0.32	--	-0.32
2824	-815	-0.1	--	-0.1
2825	-816	0.82	--	0.82
2826	-817	1.18	--	1.18
2827	-818	0.2	--	0.2
2828	-819	-0.13	--	-0.13
2829	-820	-0.76	--	-0.76
2830	-821	-0.66	--	-0.66
2831	-822	-0.75	--	-0.75
2832	-823	-0.58	--	-0.58
2833	-824	-0.74	--	-0.74
2834	-825	-0.37	--	-0.37
2835	-826	-0.43	--	-0.43
2836	-827	-0.13	--	-0.13
2837	-828	-0.46	--	-0.46
2838	-829	-0.55	--	-0.55
2839	-830	0.17	--	0.17
2840	-831	0.06	--	0.06
2841	-832	-0.46	--	-0.46
2842	-833	-0.84	--	-0.84
2843	-834	-0.76	--	-0.76
2844	-835	-0.49	--	-0.49
2845	-836	-0.73	--	-0.73
2846	-837	-0.35	--	-0.35
2847	-838	-0.08	--	-0.08
2848	-839	-0.46	--	-0.46
2849	-840	-0.27	--	-0.27
2850	-841	-0.11	--	-0.11

2851	-842	-0.16	--	-0.16
2852	-843	-0.57	--	-0.57
2853	-844	-0.24	--	-0.24
2854	-845	-0.79	--	-0.79
2855	-846	-0.16	--	-0.16
2856	-847	-0.24	--	-0.24
2857	-848	0.88	--	0.88
2858	-849	1.04	--	1.04
2859	-850	1.03	--	1.03
2860	-851	1.1	--	1.1
2861	-852	0.1	--	0.1
2862	-853	-0.04	--	-0.04
2863	-854	0.07	--	0.07
2864	-855	-0.24	--	-0.24
2865	-856	-0.29	--	-0.29
2866	-857	-0.11	--	-0.11
2867	-858	-0.14	--	-0.14
2868	-859	-0.51	--	-0.51
2869	-860	-0.35	--	-0.35
2870	-861	-0.24	--	-0.24
2871	-862	-0.27	--	-0.27
2872	-863	-0.43	--	-0.43
2873	-864	-0.24	--	-0.24
2874	-865	-0.29	--	-0.29
2875	-866	0.91	--	0.91
2876	-867	-0.22	--	-0.22
2877	-868	0.08	--	0.08
2878	-869	-0.34	--	-0.34
2879	-870	-0.57	--	-0.57
2880	-871	-0.88	--	-0.88
2881	-872	-0.43	--	-0.43
2882	-873	-0.78	--	-0.78
2883	-874	-0.93	--	-0.93
2884	-875	-0.81	--	-0.81

2885	-876	-0.69	--	-0.69
2886	-877	-0.93	--	-0.93
2887	-878	-0.59	--	-0.59
2888	-879	-0.25	--	-0.25
2889	-880	-0.32	--	-0.32
2890	-881	-0.64	--	-0.64
2891	-882	-1	--	-1
2892	-883	-0.43	--	-0.43
2893	-884	-0.19	--	-0.19
2894	-885	-0.45	--	-0.45
2895	-886	-0.52	--	-0.52
2896	-887	-0.62	--	-0.62
2897	-888	-0.79	--	-0.79
2898	-889	-0.67	--	-0.67
2899	-890	-0.68	--	-0.68
2900	-891	-0.62	--	-0.62
2901	-892	-0.19	--	-0.19
2902	-893	0.52	--	0.52
2903	-894	-0.82	--	-0.82
2904	-895	1.02	--	1.02
2905	-896	-0.05	--	-0.05
2906	-897	0.42	--	0.42
2907	-898	0.11	--	0.11
2908	-899	-0.11	--	-0.11
2909	-900	-0.12	--	-0.12
2910	-901	-0.18	--	-0.18
2911	-902	1.19	--	1.19
2912	-903	-0.24	--	-0.24
2913	-904	0.5		0.5
2914	-905	0.21		0.21
2915	-906	1.02		1.02
2916	-907	1.18		1.18
2917	-908	0.17		0.17
2918	-909	0.23		0.23

2919	-910	-0.4		-0.4
2920	-911	-0.29		-0.29
2921	-912	-0.73		-0.73
2922	-913	0.14		0.14
2923	-914	-0.54		-0.54
2924	-915	0.23	-0.97	-0.37
2925	-916	0.44	-0.69	-0.13
2926	-917	0.03	-1.02	-0.49
2927	-918	-0.05	-1.01	-0.53
2928	-919	0.14	-0.75	-0.3
2929	-920	-0.3	-0.5	-0.4
2930	-921	-0.11	-0.13	-0.12
2931	-922	0.28	-0.65	-0.18
2932	-923	-0.49	-0.34	-0.41
2933	-924	-0.95	-0.31	-0.63
2934	-925	-0.02	-0.54	-0.28
2935	-926	0.91	0.15	0.53
2936	-927	0.23	-0.39	-0.08
2937	-928	-0.65	-0.1	-0.38
2938	-929	0.25	-0.73	-0.24
2939	-930	-0.38	-0.76	-0.57
2940	-931	0	-0.6	-0.3
2941	-932	-0.27	-0.84	-0.55
2942	-933	0.47	-0.79	-0.16
2943	-934	-0.08	-1.11	-0.59
2944	-935	1.54	-1.05	0.24
2945	-936	0.71	-0.92	-0.1
2946	-937	1.04	-0.57	0.24
2947	-938	-0.02	-0.97	-0.5
2948	-939	0.91	-0.95	-0.02
2949	-940	0.68	-0.87	-0.1
2950	-941	0.02	-0.93	-0.45
2951	-942	2.11	-0.9	0.6
2952	-943	1.07	-0.86	0.11

2953	-944	0.36	-1.07	-0.35
2954	-945	-0.01	-0.95	-0.48
2955	-946	0.42	-0.87	-0.23
2956	-947	-0.64	-0.97	-0.81
2957	-948	0.21	-1.13	-0.46
2958	-949	0.06	-0.84	-0.39
2959	-950	0.03	-0.67	-0.32
2960	-951	0.17	-0.89	-0.36
2961	-952	0.11	-0.65	-0.27
2962	-953	0.18	-0.54	-0.18
2963	-954	1.37	0.5	0.94
2964	-955	2.23	0.25	1.24
2965	-956	1.37	-0.72	0.32
2966	-957	0.87	-0.56	0.15
2967	-958	-0.08	-0.75	-0.41
2968	-959	0.66	-0.46	0.1
2969	-960	0.87	-0.75	0.06
2970	-961	1.28	-0.85	0.21
2971	-962	0.41	-0.47	-0.03
2972	-963	0.1	-0.37	-0.13
2973	-964	0.48	-0.64	-0.08
2974	-965	0.78	-0.64	0.07
2975	-966	1.52	-1.05	0.23
2976	-967	0.04	-0.77	-0.36
2977	-968	2.22	-0.82	0.7
2978	-969	2.22	-0.52	0.85
2979	-970	1.55	-0.26	0.64
2980	-971	3.68	-0.17	1.75
2981	-972	3.18	-0.44	1.37
2982	-973	0.14	-0.38	-0.12
2983	-974	0.69	-0.18	0.25
2984	-975	0.85	-0.77	0.04
2985	-976	1.32	-0.22	0.55
2986	-977	0.72	-0.79	-0.03

2987	-978	-0.62	-0.62	-0.62
2988	-979	1.32	-0.39	0.46
2989	-980	0.88	-0.79	0.05
2990	-981	1.65	-0.25	0.7
2991	-982	-0.19	0.31	0.06
2992	-983	1.37	-0.41	0.48
2993	-984	0.63	-0.76	-0.06
2994	-985	0.58	-0.69	-0.06
2995	-986	0.14	-0.69	-0.27
2996	-987	0.5	-0.56	-0.03
2997	-988	-0.62	-0.49	-0.56
2998	-989	-0.21	-0.16	-0.19
2999	-990	0.5	-0.05	0.23
3000	-991	0.14	-0.37	-0.11
